# Supplementary material for: An investigation of irreproducibility in maximum likelihood phylogenetic inference
Source: Nat Commun. 2020 Nov 30;11:6096. doi: 10.1038/s41467-020-20005-6 (PMC7705714; doi:10.1038/s41467-020-20005-6)
Supplement: Supplementary file 1 — Supplementary Information [file 41467_2020_20005_MOESM1_ESM.pdf]

Supplementary Information for

An investigation of irreproducibility in maximum likelihood phylogenetic inference

Correspondence to: xingxingshen@zju.edu.cn (XXS), antonis.rokas@vanderbilt.edu (AR)

This PDF includes:

Supplementary Text
  Note 1
  Note 2
Supplementary Figures 1-15
Supplementary References

Supplementary Text

Summary of 15 phylogenomic datasets examined in this study.

Dataset	No. Taxa	No Loci	Sampling Method	Data Type	Study Reference
Animal: Bees	190	753	UCE	DNA	Blaimer et al. Evolution, 2018 [1]
Animal: Birds	200	259	AHE	DNA	Prum et al. Nature, 2015 [2]
Animal: Butterflies	207	352	AHE	DNA	Espeland et al. Current Biology, 2018 [3]
Animal: Lizards	29	1361	Exon-Capture	DNA	Blom et al. Syst Biol, 2017 [4]
Animal: Marine fishes	120	1001	UCE	DNA	Alfaro et al. 2018, Nat. Ecol. Evol.[5]
Animal: Rodents	37	1245	Exon-Capture	DNA	Roycroft et al. Syst Biol, 2019 [6]
Plant: Cardueae	85	570	UCE	DNA	Herrando-Moraira et al. Mol Phyloge Evol, 2018 [7]
Plant: Caryophyllales	95	1122	Transcriptome	AA	Yang et al. Mol Biol Evol, 2015 [8]
Plant: Green plants	1178	410	Transcriptome	AA	1KP Initiative 2019, Nature [9]
Plant: Jaltomata	15	6431	Transcriptome	DNA	Wu et al. Mol Ecol, 2018 [10]
Plant: Protea	65	498	AHE	DNA	Mitchell et al. American Journal of Botany, 2017 [11]
Fungi: Aspergillaceae	93	1668	Genome	DNA	Steenwyk et al. mBio, 2019 [12]
Fungi: Budding yeasts	343	2408	Genome	AA	Shen et al. 2018, Cell [13]
Fungi: Hanseniaspora	29	1033	Genome	AA	Steenwyk et al. PloS Biol, 2019 [14]
Fungi: Rhizoplaca	31	303	Genome	DNA	Leavitt et al., Sci Rep, 2016 [15]

UCE: Ultraconserved Element, AHE: Anchored Hybrid Enriched, DNA: deoxyribonucleic acid, AA: amino acid.

Note 1. Commands, parameter settings, and job submission files used in gene tree inference

For each of the 19,414 gene alignments from 15 phylogenomic studies in animals, plants, and fungi, we executed two replicates (Run1 and Run2) to test whether phylogenetic tree inferred by Run1 analysis is topologically identical to phylogenetic tree inferred by Run2 or not (Fig.1). The two replicates used exactly the same parameter settings including ML program, gene alignment, substitution model, random seed, number of threads, number of tree searches, and log-likelihood epsilon for optimization.

1. Gene tree inference on DNA gene alignments

For all DNA gene alignments, the GTR+G4+F substitution model was used.

Given a DNA gene alignment file named ***L1.fasta***, below are the commands and parameter settings used for inferring gene trees with 2 replicates in IQ-TREE multi-thread version 1.6.8 and in RAxML-NG multi-thread version 0.9.0, respectively.

IQ-TREE

```
iqtree -s L1.fasta -st DNA -m GTR+G4+F -seed 4760742 -nt 2 --runs 20
-me 0.0001 -pre iqtree_L1_run1
iqtree -s L1.fasta -st DNA -m GTR+G4+F -seed 4760742 -nt 2 --runs 20
-me 0.0001 -pre iqtree_L1_run2
```

RAxML-NG

```
raxml-ng --msa L1.fasta --search -msa-format FASTA --model GTR+G4+F
-seed 967956542 --threads 2 --tree pars{10},rand{10} --lh-epsilon
0.0001 --prefix raxml-ng_L1_run1
raxml-ng --msa L1.fasta --search -msa-format FASTA --model GTR+G4+F
-seed 967956542 --threads 2 --tree pars{10},rand{10} --lh-epsilon
0.0001 --prefix raxml-ng_L1_run2
```

2. Gene tree inference on amino acid gene alignments

For amino acid sequence alignments, the best-fitting substitution model reported in original study was used (all substitution models are available on the figshare depository). For genes in the green plant dataset (410 genes and 1,178 taxa) and the budding yeast dataset (2,408 genes and 343 taxa), we set the number of tree searches to two (IQ-TREE with "--runs 2" option; RAxML-NG with "--tree pars{1},rand{1}" option) because of computetime limitations (three days) on the supercomputing cluster.

Given an amino acid alignment file named ***ORTHOMCL1000.fasta***, below are the commands and parameter settings used for inferring gene trees with 2 replicates using IQ-TREE multi-thread version 1.6.8 and RAxML-NG multi-thread version 0.9.0, respectively.

IQ-TREE

```
iqtree -s ORTHOMCL1000.fasta -st AA -m LG+G4 -seed 529418945 -nt 2 -
-runs 20 -me 0.0001 -pre iqtree_ORTHOMCL1000_run1
iqtree -s ORTHOMCL1000.fasta -st AA -m LG+G4 -seed 529418945 -nt 2 -
-runs 20 -me 0.0001 -pre iqtree_ORTHOMCL1000_run2
```

RAxML-NG

```
raxml-ng --msa ORTHOMCL1000.fasta --search -msa-format FASTA --model
LG+G4 -seed 652954101 --threads 2 --tree pars{10},rand{10} --lh-
epsilon 0.0001 --prefix raxml-ng_ORTHOMCL1000_run1
raxml-ng --msa ORTHOMCL1000.fasta --search -msa-format FASTA --model
LG+G4 -seed 652954101 --threads 2 --tree pars{10},rand{10} --lh-
epsilon 0.0001 --prefix raxml-ng_ORTHOMCL1000_run2
```

3. Assessment of reproducibility of single-gene phylogenies inferred by Run1 and Run2

For a given gene alignment ***L1***, its phylogenetic inference is reproducible if two gene trees inferred from two replicates (Run1 and Run2) are topologically identical (i.e., the normalized Robinson–Foulds tree distance = 0); in contrast, its phylogenetic inference is irreproducible if two gene trees inferred from two replicates (Run1 and Run2) are topologically different from each other (i.e., the normalized Robinson–Foulds tree distance > 0). The normalized Robinson–Foulds tree distance (normalized RFD) between two gene trees inferred from two replicates (Run1 and Run2) was calculated by the raxmlHPC-PTHREADS-SSE3 version 8.2.3[16].

```
raxmlHPC-PTHREADS-SSE3 -T 2 -f r -m GTRGAMMA -z
iqtree_L1_run12.trees -n iqtree_L1_run12_rf
raxmlHPC-PTHREADS-SSE3 -T 2 -f r -m GTRGAMMA -z raxml-
ng_L1_run12.trees -n raxml-ng_L1_run12_rf -z raxml-ng_L1_run12.trees
-n raxml-ng_L1_run12_rf
```

If two single-gene trees inferred from two replicates (Run1 and Run2) are topologically different from each other, we used the approximately unbiased (AU) test [17] to examine whether Run1 and Run2 yielded two significantly different single-gene trees (P-value $\leqslant$ 0.05). The AU test was implemented in IQ-TREE multi-thread version 1.6.8.

```
iqtree -nt 3 -st DNA -seed 123456 -me 0.0001 -s L1.fasta -m GTR+G4+F
-z iqtree_L1_run12.trees(or raxml-ng_L1_run12.trees) -zb 1000 -n 0 -
au -pre $output_AU
```

4. Assessment of reliability of each internal branch of single-gene phylogenies inferred by Run1 and Run2

Since running all 19,414 gene alignments from 15 phylogenomic datasets was computationally intractable, we sampled the first 100 genes from each dataset. For each of the 1,500 gene alignments from 15 phylogenomic studies in animals, plants, and fungi, we executed two replicates (Run1 and Run2) to evaluate the support of each internal branch using 1,000 ultrafast bootstrap replicates (the option "-bb 1000") and 100 standard bootstrap replicates (the option "--bs-trees 100") for IQ-TREE analysis and RAxML-NG, respectively.

DNA gene alignments

For all DNA gene alignments, the GTR+G4+F substitution model was used.

Given a DNA gene alignment file named ***L1.fasta***, below are the commands and parameter settings used for inferring gene trees with 2 replicates in IQ-TREE multi-thread version 1.6.8 and in RAxML-NG multi-thread version 0.9.0, respectively.

IQ-TREE

```
iqtree -s L1.fasta -st DNA -m GTR+G4+F -seed 4760742 -nt 2 --runs 10
```

```
-me 0.0001 -bb 1000 -pre iqtree_L1_run1
iqtree -s L1.fasta -st DNA -m GTR+G4+F -seed 4760742 -nt 2 --runs 10
-me 0.0001 -bb 1000 -pre iqtree_L1_run2
```

RAxML-NG

```
raxml-ng --msa L1.fasta --all -msa-format FASTA --model GTR+G4+F -
seed 967956542 --threads 2 --tree pars{5},rand{5} --lh-epsilon
0.0001 --bs-trees 100 --prefix raxml-ng_L1_run1
raxml-ng --msa L1.fasta --all -msa-format FASTA --model GTR+G4+F -
seed 967956542 --threads 2 --tree pars{5},rand{5} --lh-epsilon
0.0001 --bs-trees 100 --prefix raxml-ng_L1_run2
```

Amino acid gene alignments

For amino acid sequence alignments, the best-fitting substitution model reported in original study was used (all substitution models are available on the figshare depository).

Given an amino acid alignment file named ***ORTHOMCL1000.fasta***, below are the commands and parameter settings used for inferring gene trees with 2 replicates using IQ-TREE multi-thread version 1.6.8 and RAxML-NG multi-thread version 0.9.0, respectively.

IQ-TREE

```
iqtree -s ORTHOMCL1000.fasta -st AA -m LG+G4 -seed 529418945 -nt 2 -
-runs 10 -me 0.0001 -bb 1000 -pre iqtree_ORTHOMCL1000_run1
iqtree -s ORTHOMCL1000.fasta -st AA -m LG+G4 -seed 529418945 -nt 2 -
-runs 10 -me 0.0001 -bb 1000 -pre iqtree_ORTHOMCL1000_run2
```

RAxML-NG

```
raxml-ng --msa ORTHOMCL1000.fasta --all -msa-format FASTA --model
LG+G4 -seed 652954101 --threads 2 --tree pars{5},rand{5} --lh-
epsilon 0.0001 --bs-trees 100 --prefix raxml-ng_ORTHOMCL1000_run1
raxml-ng --msa ORTHOMCL1000.fasta --all -msa-format FASTA --model
LG+G4 -seed 652954101 --threads 2 --tree pars{5},rand{5} --lh-
epsilon 0.0001 --bs-trees 100 --prefix raxml-ng_ORTHOMCL1000_run2
```

Job submission on the CHTC supercomputing cluster (University of Wisconsin-Madison)

Execution of jobs on the CHTC cluster uses a software called HTCondor:

1) Two replicates (Run1 and Run2) submitted to two separate nodes

Given that there is a DNA gene alignment file named ***L1.fasta,*** below are the job submission files for running IQ-TREE and RAxML-NG.

IQ-TREE

```
universe = vanilla
log = file.log
error = file.err
output = file.out
should_transfer_files = Yes
when_to_transfer_output = ON_EXIT
request_cpus = 2
request_memory = 1GB
request_disk = 100MB
getenv = true
requirements = (OpSysMajorVer == 7)
executable = /home/nu_shenx3/tools/iqtree-1.6.8-Linux/bin/iqtree
transfer_input_files = ../data/L1.fasta
Arguments = -s L1.fasta -st DNA -m GTR+G4+F -seed 4760742 -nt 2 --runs 20 -me 0.0001 -pre iqtree_L1_run1
queue
executable = /home/nu_shenx3/tools/iqtree-1.6.8-Linux/bin/iqtree
transfer_input_files = ../data/L1.fasta
Arguments = -s L1.fasta -st DNA -m GTR+G4+F -seed 4760742 -nt 2 --runs 20 -me 0.0001 -pre iqtree_L1_run2
queue
```

RAxML-NG.

```
universe = vanilla
log = file.log
error = file.err
output = file.out
should_transfer_files = Yes
when_to_transfer_output = ON_EXIT
request_cpus = 2
request_memory = 1GB
request_disk = 100MB
getenv = true
requirements = (OpSysMajorVer == 7)
executable = /home/nu_shenx3/tools/raxml-ng-0.9.0/raxml-ng
transfer_input_files = ../data/L1.fasta
Arguments = --msa L1.fasta -msa-format FASTA --model GTR+G4+F -seed 967956542 --threads 2 --tree
pars{10},rand{10} --lh-epsilon 0.0001 --prefix raxml-ng_L1_run1
queue
executable = /home/nu_shenx3/tools/raxml-ng-0.9.0/raxml-ng
transfer_input_files = ../data/L1.fasta
Arguments = --msa L1.fasta -msa-format FASTA --model GTR+G4+F -seed 967956542 --threads 2 --tree
pars{10},rand{10} --lh-epsilon 0.0001 --prefix raxml-ng_L1_run2
queue
```

2) Two replicates (Run1 and Run2) submitted to a single node (two replicates ran one after the anther with same processor on a single node)

Given that there is a DNA gene alignment file named ***L1.fasta,*** below are the job submission files for running IQ-TREE and RAxML-NG.

IQ-TREE

```
universe = vanilla
log = file.log
error = file.err
output = file.out
should_transfer_files = Yes
when_to_transfer_output = ON_EXIT
request_cpus = 2
request_memory = 1GB
request_disk = 100MB
getenv = true
requirements = (OpSysMajorVer == 7)
executable = iqtree_L1.fasta_run12.sh
transfer_input_files = ../data/L1.fasta,/home/nu_shenx3/tools/iqtree-1.6.8-Linux/bin/iqtree
queue
```

iqtree_L1.fasta_run12.sh

```
#!/bin/bash
./iqtree -s L1.fasta -st DNA -m GTR+G4+F -seed 4760742 -nt 2 --runs 20 -me 0.0001 -pre iqtree_L1_run1
./iqtree -s L1.fasta -st DNA -m GTR+G4+F -seed 4760742 -nt 2 --runs 20 -me 0.0001 -pre iqtree_L1_run2
```

RAxML-NG.

```
universe = vanilla
log = file.log
error = file.err
output = file.out
should_transfer_files = Yes
when_to_transfer_output = ON_EXIT
request_cpus = 2
request_memory = 1GB
request_disk = 100MB
getenv = true
requirements = (OpSysMajorVer == 7)
executable = raxml-ng_L1.fasta_run12.sh
transfer_input_files = ../data/L1.fasta, /home/nu_shenx3/tools/raxml-ng-0.9.0/raxml-ng
queue
```

raxml-ng_L1.fasta_run12.sh

```
#!/bin/bash
./raxml-ng --msa L1.fasta -msa-format FASTA --model GTR+G4+F -seed 967956542 --threads 2 --tree
pars{10},rand{10} --lh-epsilon 0.0001 --prefix raxml-ng_L1_run1
./raxml-ng --msa L1.fasta -msa-format FASTA --model GTR+G4+F -seed 967956542 --threads 2 --tree
pars{10},rand{10} --lh-epsilon 0.0001 --prefix raxml-ng_L1_run2
```

Simulations

To the accuracy of gene tree estimation for irreproducible and reproducible genes, we first generated 15 balanced trees and 15 star trees, both with 64 taxa, each of which was scaled by branch length α (α = 0.001, 0.002, 0.003, 0.004, 0.005, 0.006, 0.007, 0.008, 0.009, 0.01, 0.02, 0.04, 0.06, 0.08, or 0.1) (Fig. 5a), respectively. Next, each reference species tree ***T_α.newick*** was used to generate 500 nucleotide gene alignments with randomly varying length ***L*** from 300 to 1,000 base pairs using Seq-Gen.v1.3.2 [18] under the GTR +G4 model, shape for the gamma rate heterogeneity =1, proportion of invariable Sites=0, and equal state frequency.

```
seq-gen -mGTR -a1 -g4 -i0 -f0.25,0.25,0.25,0.25 -lL -n1 -or -
z[Random seed number] < T_α.newick > T_α_genei.phy
```

T_α.newick is one of 30 64-taxon trees
L is randomized between 300 and 1,000
Random seed number: int (rand(10000000))+1 in custom perl;
i is from 1 to 500

A total number of simulated gene alignments is 15,000 (30 reference trees * 500 genes). Detailed values are given in the file entitled “Seq_Gen_run.bat” on the figshare repository.

Note 2. Suggestions for increasing the reproducibility of phylogenetic analyses

Our results showed that while the public availability of gene sequence alignment, program, substitution model, and number of independent tree searches are prerequisites for the reproducibility of phylogenies, they alone are not sufficient. We found that random starting seed number, number of threads, and processor type can also affect the reproducibility of ML phylogenies.

a) Random seed number can be specified in IQ-TREE[19] and RAxML-NG[20] with the "-seed" option and the "--seed" option, respectively.

b) Number of threads can be specified in IQ-TREE and RAxML-NG with the "-nt" option and the "--threads" option, respectively. Note that the use of three or more threads in the current version of IQ-TREE substantially increases the chance of topological irreproducibility and different log-likelihood scores when gene alignments with low numbers of parsimony-informative sites are analyzed[21,22] because of the different addition orders of the per-site log likelihoods (when three or more threads are used).

c) Processor settings in RAxML-NG can be specified for the kernel with the "--simd" option. To avoid the variation of kernels between processors, users can specify the use of a specific set of vector instructions (e.g., "--simd sse3") or set the analysis to be performed using non-vectorized kernels with the "--simd none" option. IQ-TREE does not provide an option for the specification of the kernel.

A more practical way may be the releasing of the log file of each analysis, which contains a record of the values of all these key parameters (e.g., alignment, program name, number of tree searches, substitution model, type of processor, number of threads, and random starting seed).

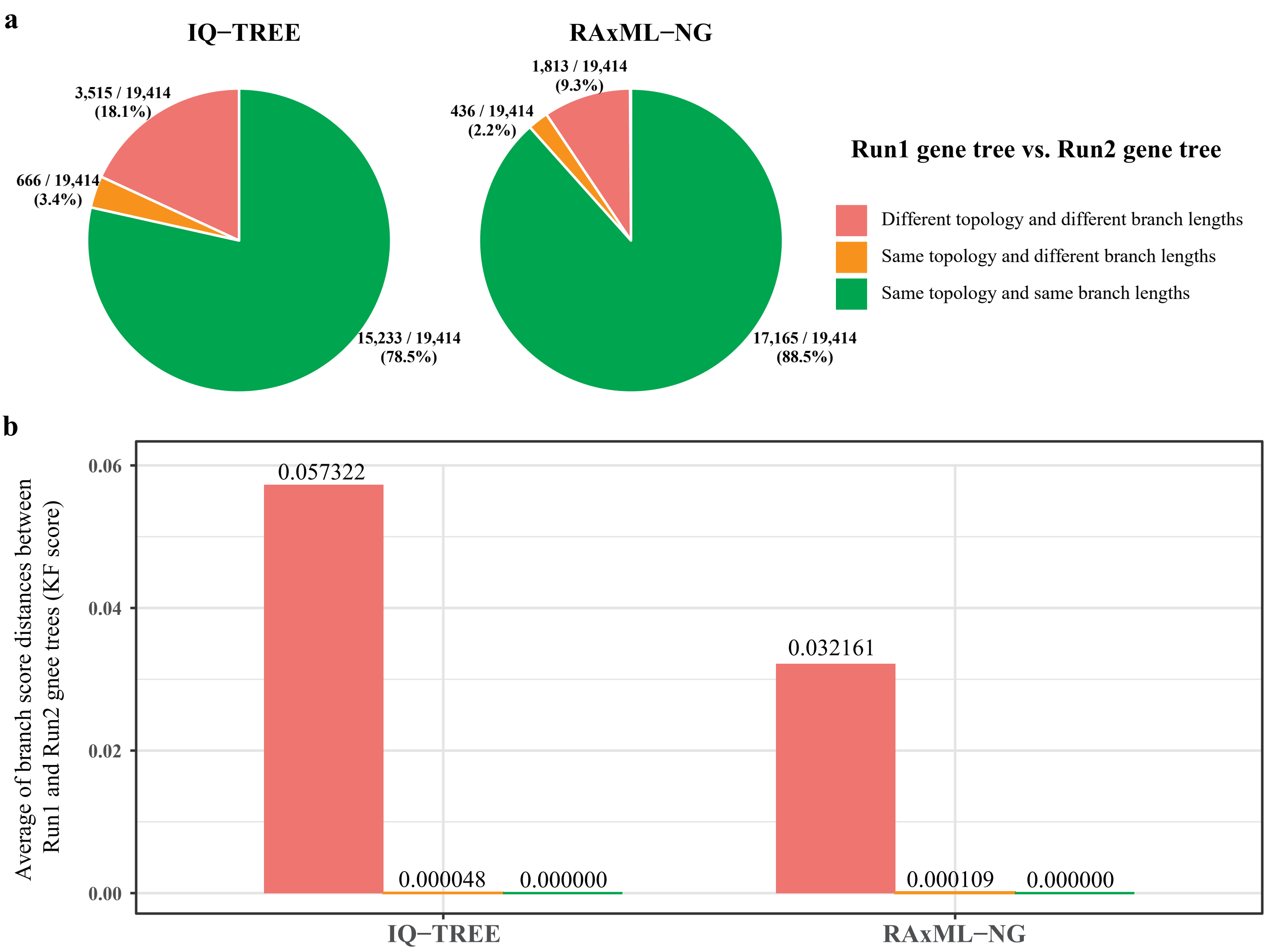

Supplementary Figure 1. Comparisons of topology and branch lengths between two gene trees inferred by two replicates (Run1 and Run2). To compare topology and branch lengths between two gene trees, normalized Robinson–Foulds tree distance (nRFD) and branch score distance of Kuhner and Felsenstein (KF) are computed with the R packages ape and phangorn, respectively. a) Using IQ-TREE, 3,515 / 19,414 (18.1%) gene alignments from 15 phylogenomic datasets yielded different topologies (nRFD > 0) and different branch lengths (KF >0) between two replicates (red), 666 (3.4%) yielded same topologies (nRFD = 0) and different branch lengths (KF > 0) (yellow), while the remaining 15,233 (78.5%) yielded same topologies (nRFD = 0) and different branch lengths (KF = 0) (green). Using RAxML-NG, 1,813 (9.3%) genes yielded different topologies (nRFD > 0) and different branch lengths (KF > 0)between two replicates (red), 436 (2.2%) yielded same topologies (nRFD = 0) and different branch lengths (KF >0) (yellow), while the remaining 17,165 (88.5%) yielded same topologies (nRFD = 0) and same branch lengths (KF = 0) (green). b) For each category, we calculated overall mean of the branch score distances. All 77,656 analyses (19,414 alignments * 2 replicates * 2 ML programs) were run using 2 threads per node on the Center for High Throughput Computing (CHTC) at the University of Wisconsin-Madison.

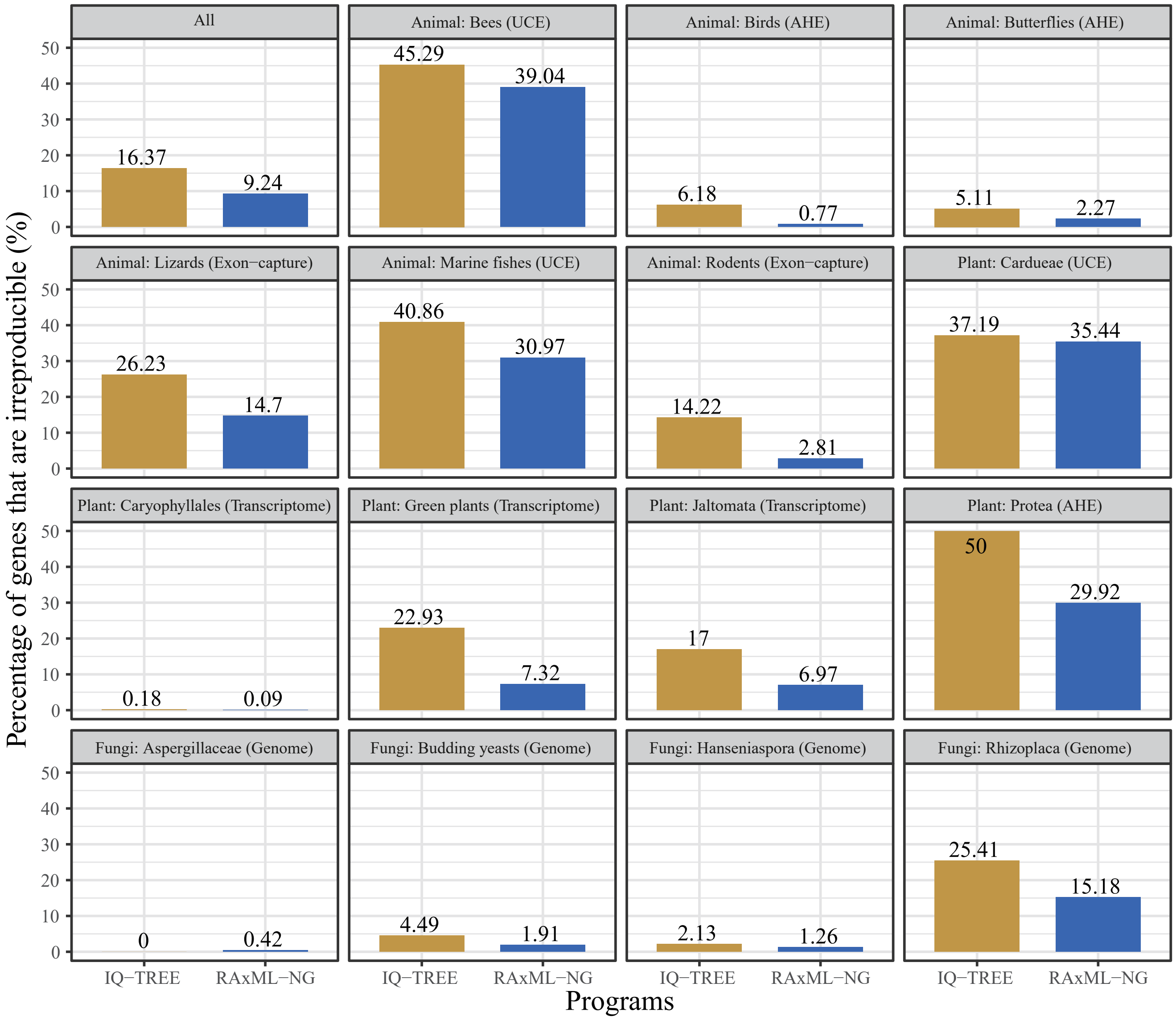

Supplementary Figure 2. A considerable number of genes in phylogenomic datasets produce irreproducible phylogenies on the ACCRE supercomputing cluster. The reproducibility of phylogenies of 19,414 genes from 15 phylogenomic datasets was assessed between two replicates (Run1 and Run2) that ran on two separate nodes on 2 CPU cores (threads) using IQ-TREE and RAxML-NG, respectively. Bar plots show the percentages of genes that are irreproducible when using IQ-TREE (in yellow) and RAxML-NG (in blue), respectively. The bar plot at the upper left is based on all 19,414 genes from 15 phylogenomic datasets (see Supplementary Data 1); it shows that the phylogenies of 3,178 / 19,414 genes (16.37%) and 1,793 / 19,414 genes (9.24%) are irreproducible between two replicates (Run1 and Run2) using IQ-TREE (in yellow) and RAxML-NG (in blue), respectively. The rest of the bar plots show the individual results for each of the 15 phylogenomic datasets. These datasets were constructed using five different but widely accepted gene sampling approaches (shown in parentheses): Ultraconserved Element (UCE) capture, Anchored Hybrid Enriched (AHE) capture, conserved exon capture, transcriptome sequencing, and whole genome sequencing. All 77,656 analyses (19,414 genes * 2 replicates * 2 ML programs) were run on the Advanced Computing Center for Research and Education (ACCRE) at Vanderbilt University. Detailed values are given in Supplementary Data 3.

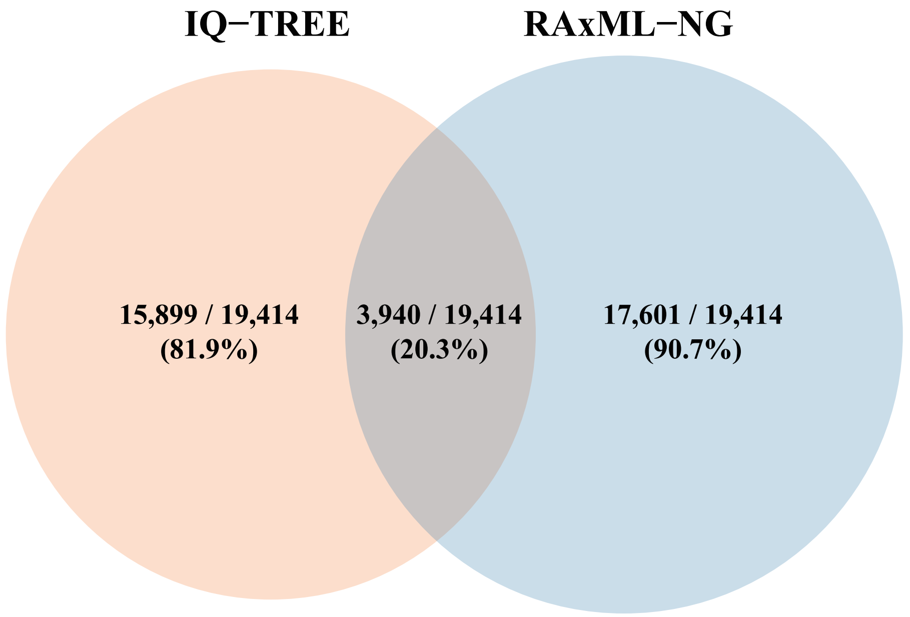

Supplementary Figure 3. A small number of genes in phylogenomic datasets produce reproducible phylogenies between IQ-TREE and RAxML-NG. 15,899/ 19,414 genes (81.9%) and 17,601 / 19,414 genes (90.7%) yielded topologically reproducible phylogenies between two replicates (Run1 and Run2) using IQ-TREE (in yellow) and RAxML-NG (in blue), respectively. However, the number of genes that yielded topologically reproducible phylogenies between IQ-TREE and RAxML-NG is 3,940 (20.3%). All 77,656 analyses (19,414 alignments * 2 replicates * 2 ML programs) were run using 2 threads per node on the Center for High Throughput Computing (CHTC) at the University of Wisconsin-Madison.

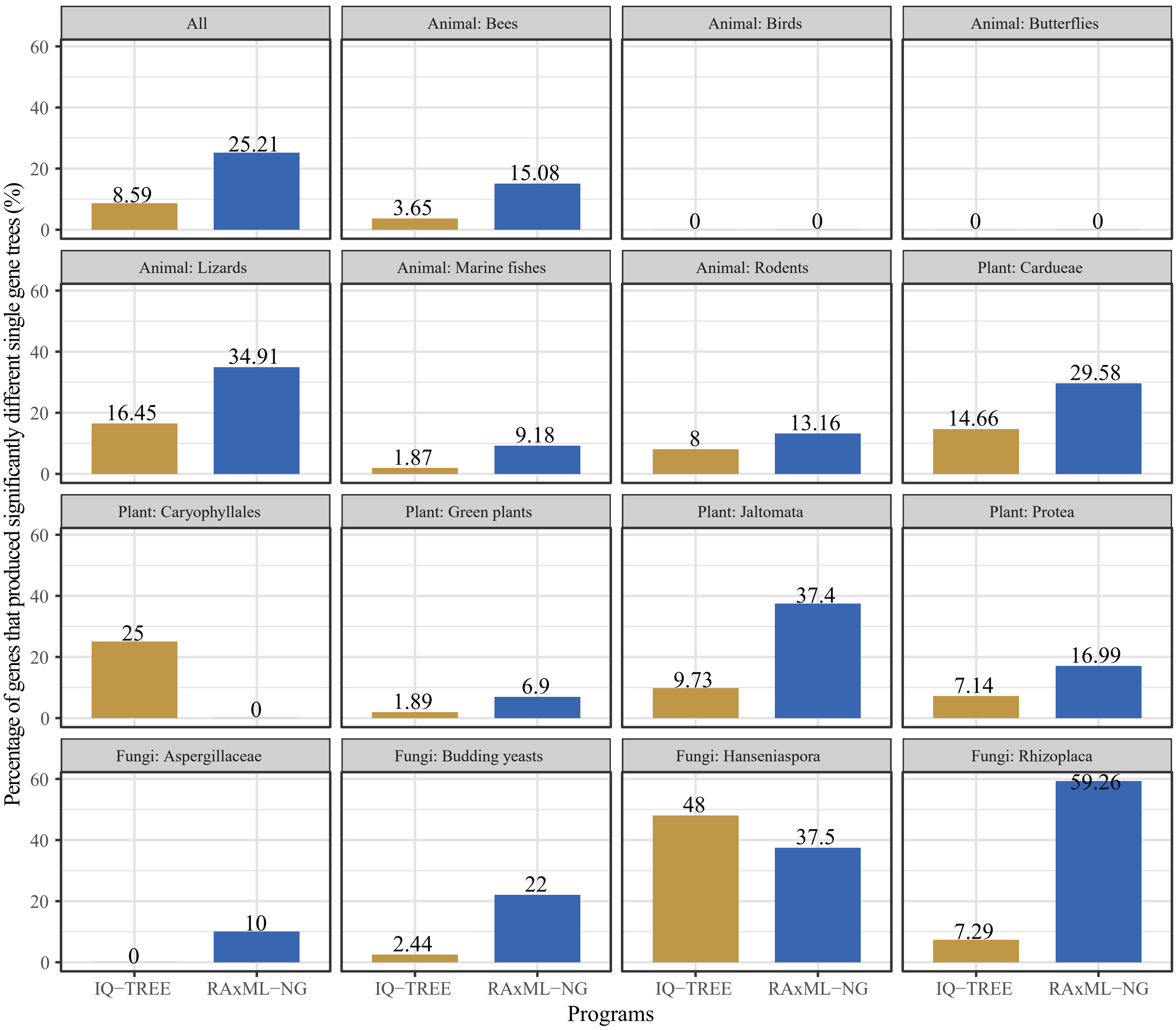

Supplementary Figure 4. Percentage of genes that that produced significantly irreproducible phylogenetic trees between two replicates in phylogenomic datasets on the CHTC supercomputing cluster. Bar plots show the percentages of genes produced significantly irreproducible phylogenetic trees (the two-sided AU test; *P*-value ≤ 0.05) between two replicates when using IQ-TREE (in yellow) and RAxML-NG (in blue), respectively. The bar plot at the upper left is based on all 19,414 genes from 15 phylogenomic datasets (see Supplementary Data 1); it shows the Run1 and Run2 topologies for 302 / 3,515 (8.59%) irreproducible single-gene ML phylogenies generated by IQ-TREE and 457 / 1,813 (25.2%) irreproducible phylogenies generated by RAxML were significantly different (the approximately unbiased (AU) test; *P*-value ≤ 0.05). The rest of the bar plots show the individual results for each of the 15 phylogenomic datasets.

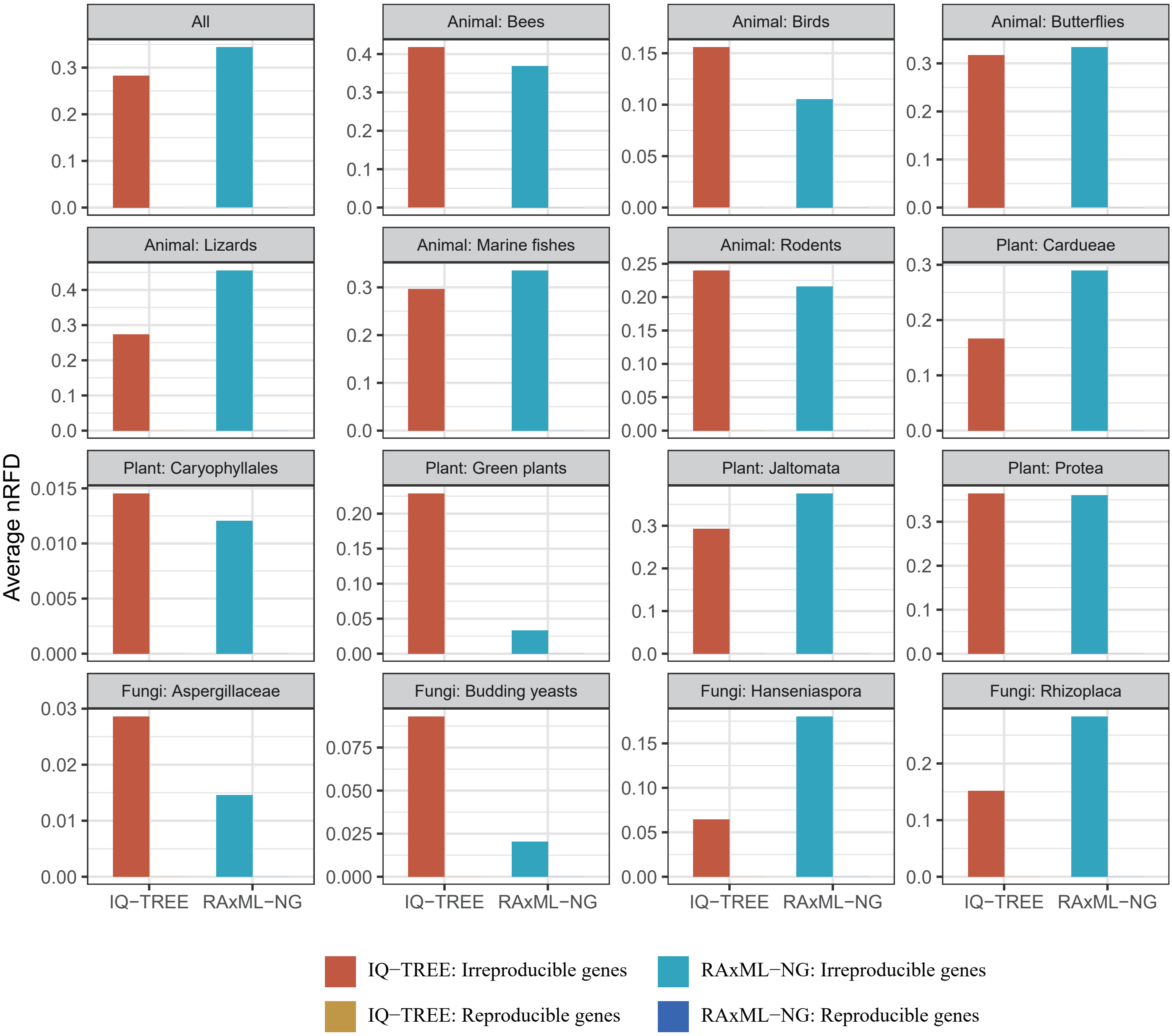

Supplementary Figure 5. Average tree distance between Run1 and Run2 replicates of 19,414 genes in 15 phylogenomic datasets. For each gene, the normalized Robinson–Foulds tree distance (nRFD) was estimated based on two gene trees inferred from the Run1 and Run2 replicates. Bar plot at upper left is based on all 19,414 genes from 15 phylogenomic datasets. All other bar plots show the results of each of the 15 phylogenomic datasets. Genes yielding irreproducible phylogenies are colored in red and genes yielding reproducible phylogenies are colored in yellow when using IQ-TREE; genes yielding irreproducible phylogenies are colored in green and genes yielding reproducible phylogenies are colored in blue when using RAxML-NG. Detailed values are given in Supplementary Data 2.

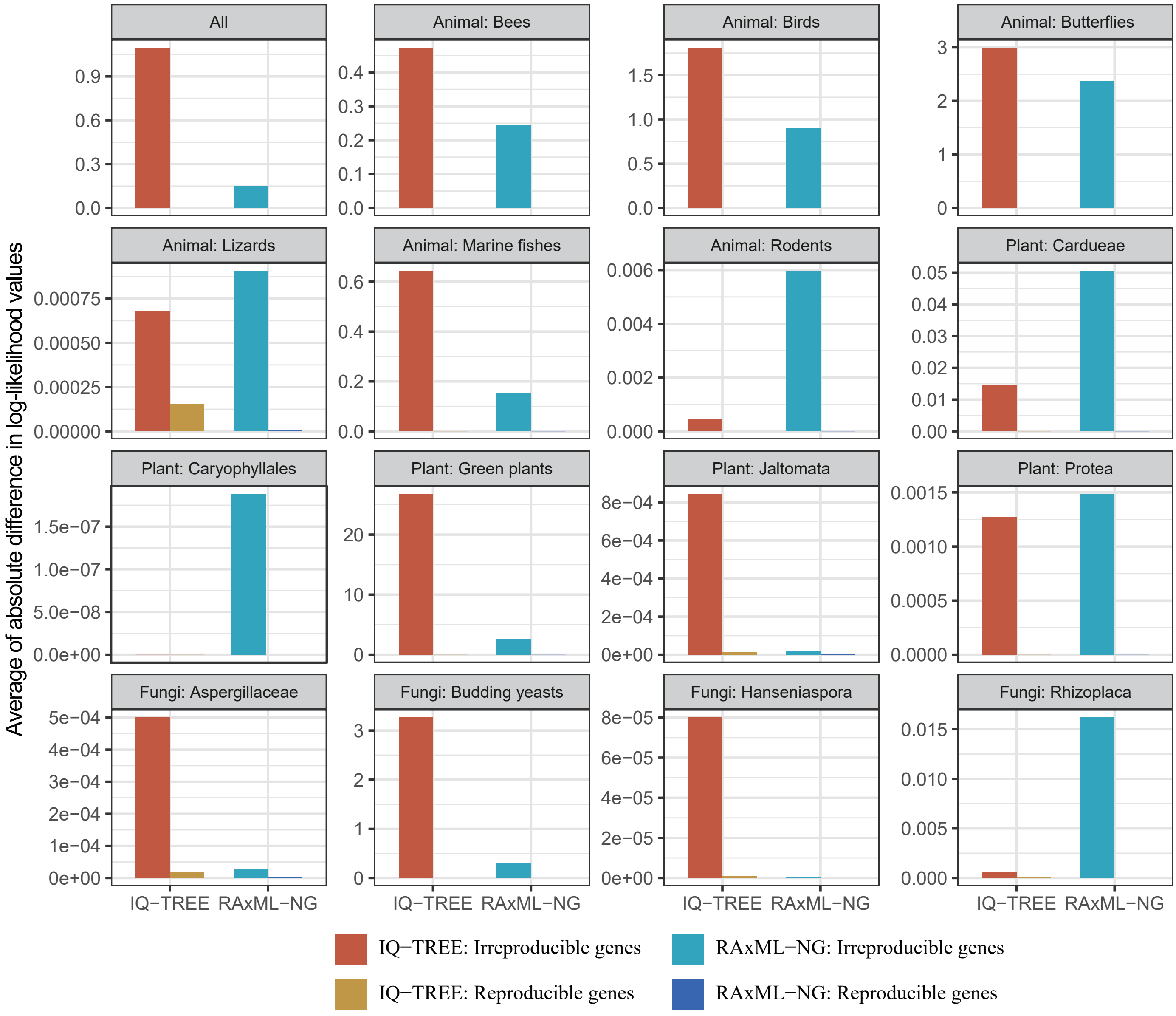

Supplementary Figure 6. Average absolute difference in log-likelihood values between Run1 and Run2 replicates of 19,414 genes in 15 phylogenomic datasets. For each gene, we calculated the absolute difference in log-likelihood values between Run1 and Run2 replicates. Bar plot at upper left is based on all 19,414 genes from 15 phylogenomic datasets. All other bar plots show the results for each of the 15 phylogenomic datasets. Genes yielding irreproducible phylogenies are colored in red and genes yielding reproducible phylogenies are colored in yellow when using IQ-TREE; genes yielding irreproducible phylogenies are colored in green and genes yielding reproducible phylogenies are colored in blue when using RAxML-NG. Detailed values are given in Supplementary Data 2.

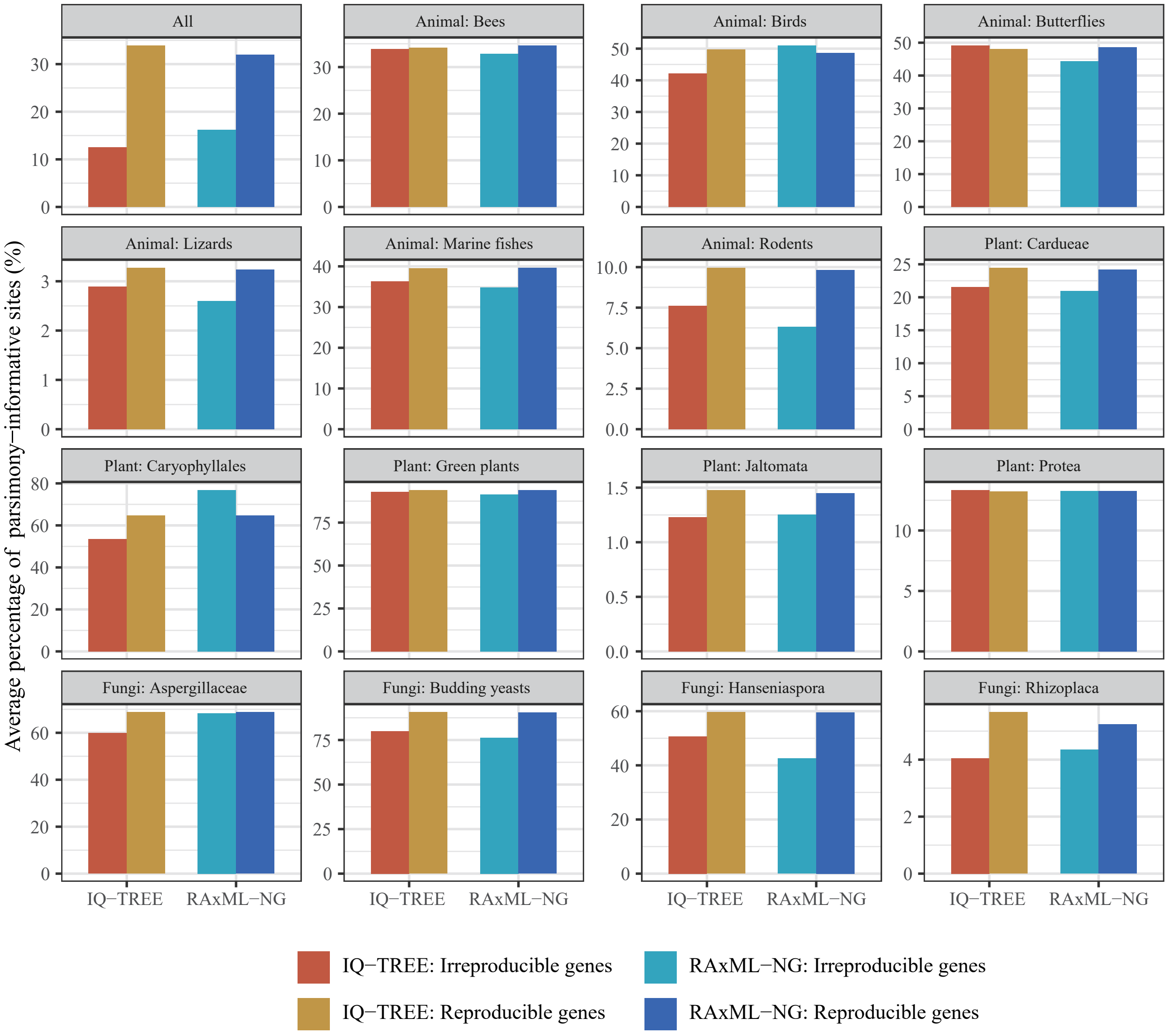

Supplementary Figure 7. Average percentage of parsimony-informative sites of 19,414 genes in 15 phylogenomic datasets Bar plot at upper left is based on all 19,414 genes from 15 phylogenomic datasets. All other bar plots show the results for each of the 15 phylogenomic datasets. Genes yielding irreproducible phylogenies are colored in red and genes yielding reproducible phylogenies are colored in yellow when using IQ-TREE; genes yielding irreproducible phylogenies are colored in green and genes yielding reproducible phylogenies are colored in blue when using RAxML-NG. Detailed values are given in Supplementary Data 2.

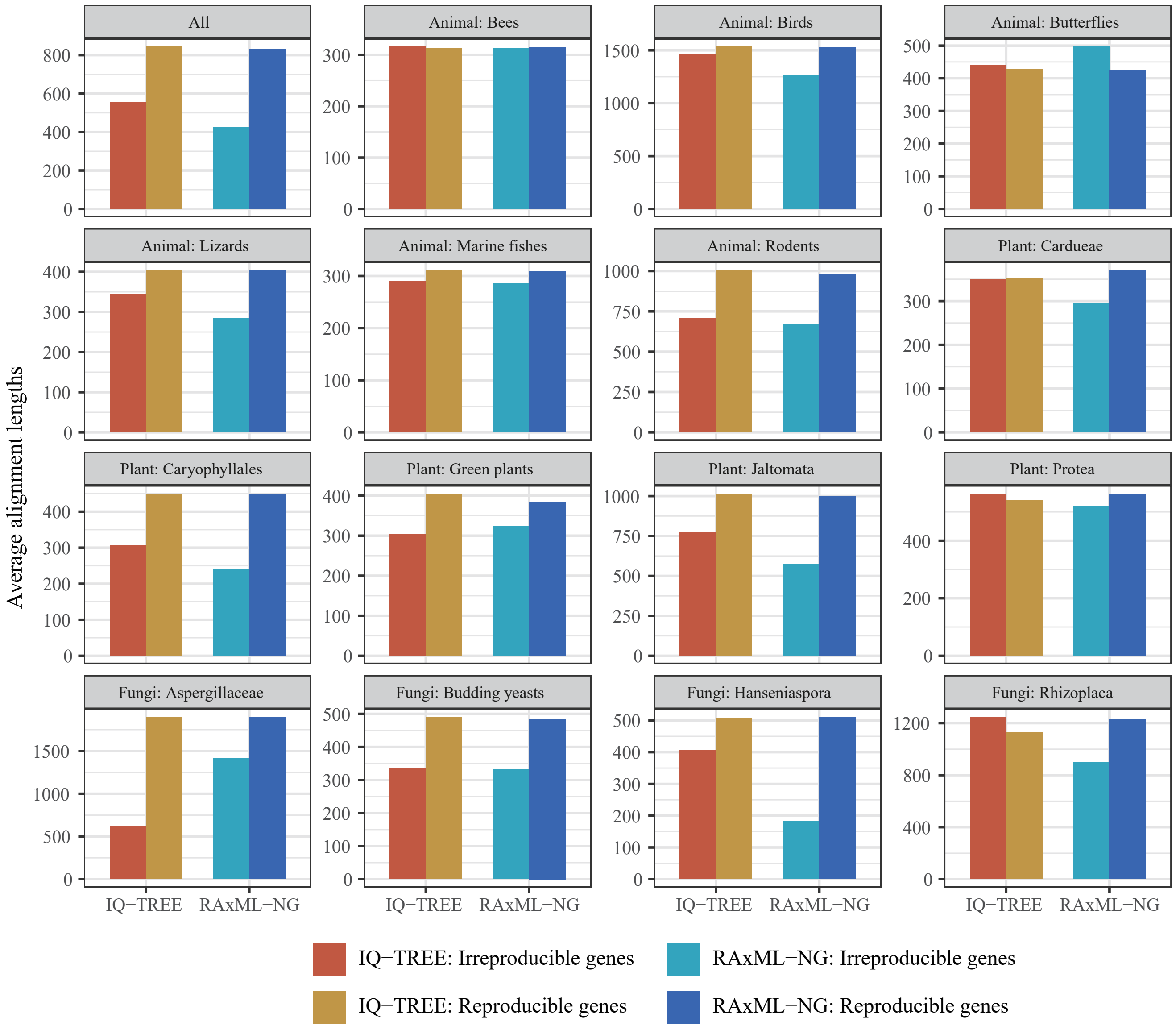

Supplementary Figure 8. Average alignment length in 15 phylogenomic datasets Bar plot at upper left is based on all 19,414 genes from 15 phylogenomic datasets. All other bar plots show the results for each of the 15 phylogenomic datasets. Genes yielding irreproducible phylogenies are colored in red and genes yielding reproducible phylogenies are colored in yellow when using IQ-TREE; genes yielding irreproducible phylogenies are colored in green and genes yielding reproducible phylogenies are colored in blue when using RAxML-NG. Detailed values are given in Supplementary Data 2.

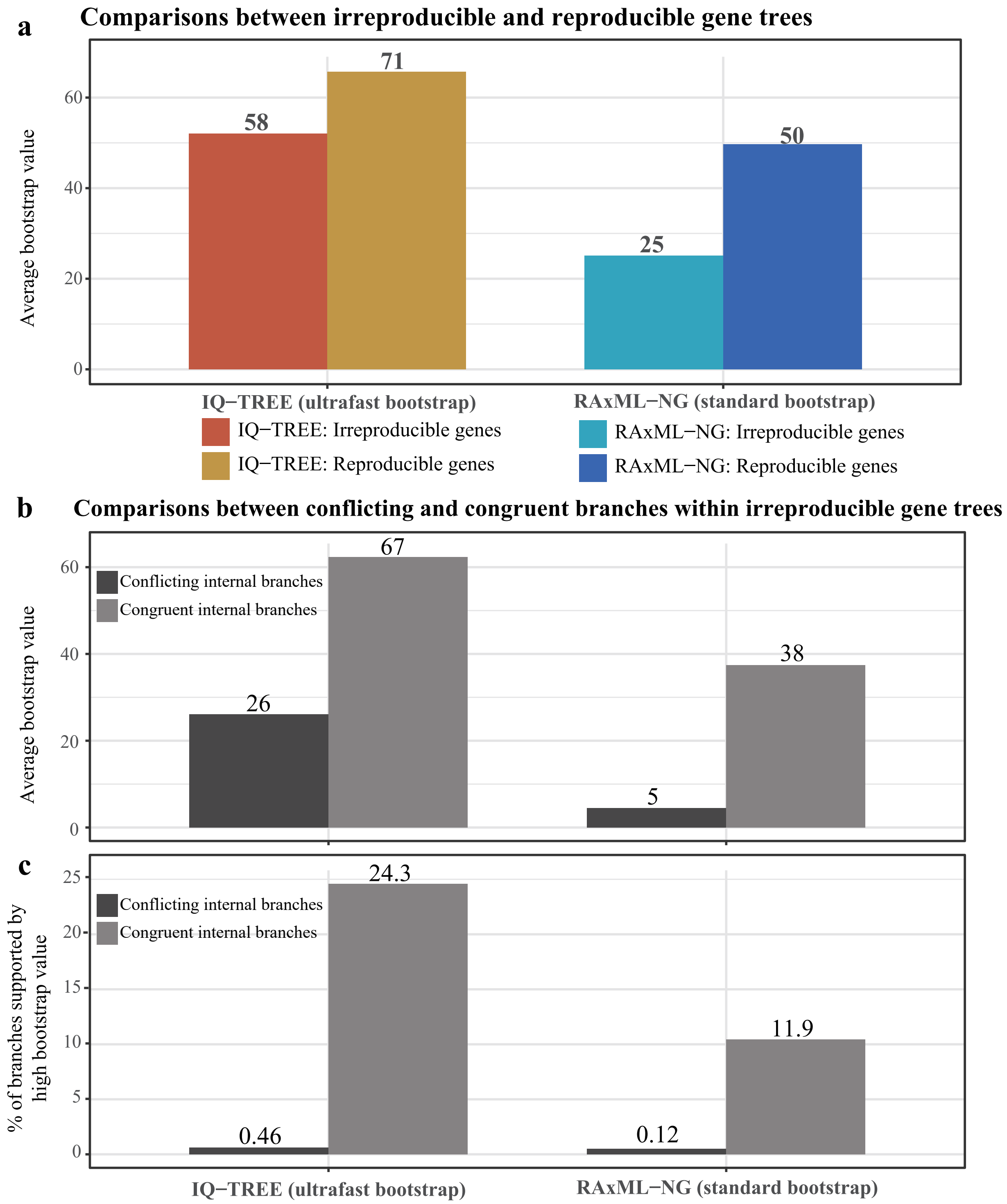

Supplementary Figure 9. Comparisons of bootstrap support values between genes that yield irreproducible phylogenies and reproducible ones. Since assessing bootstrap support for all gene trees is computationally intractable, we sampled the first 100 genes from each of 15 phylogenomic datasets. Using IQ-TREE, 410 / 1,500 (27.3%) gene alignments yielded irreproducible phylogenies between two replicates, while the remaining 1,090 / 1,500 (72.7%) yielded reproducible phylogenies. Using RAxML-NG, 183 / 1,500 (12.2 %) genes yielded irreproducible phylogenies between two replicates (green bars), while 1,317 / 1,500 (87.8%) yielded reproducible phylogenies. For the sets of reproducible and irreproducible genes in IQ-TREE and RAxML-NG analyses, (a) shows overall mean bootstrap values. For the irreproducible genes in IQ-TREE and RAxML-NG analyses, we assessed (b) bootstrap support values of conflicting internal branches and congruent internal branches within irreproducible genes (i.e., genes that yield two topologically different trees in Run1 and Run2); we also examined (c) percentage of internal branches strongly supported by rapid bootstrap value of ≥ 95 in IQ-TREE analyses and standard bootstrap value of ≥70 in RAxML-NG analyses. All 77,656 analyses (19,414 alignments * 2 replicates * 2 ML programs) were run using 2 threads per node on the Center for High Throughput Computing (CHTC) at the University of Wisconsin-Madison.

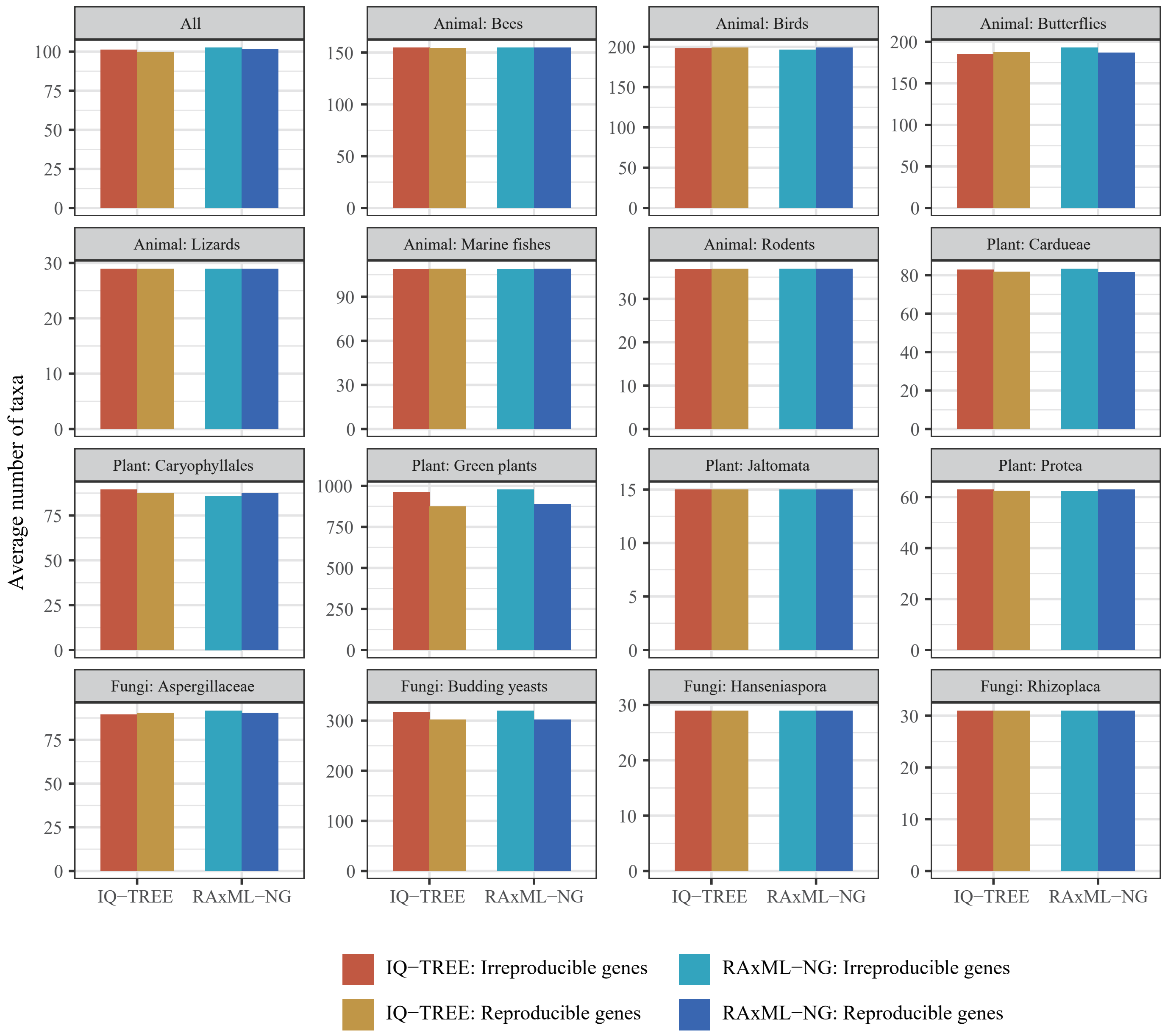

Supplementary Figure 10. Average number of taxa in 15 phylogenomic datasets Bar plot at upper left is based on all 19,414 genes from 15 phylogenomic datasets. All other bar plots show the results for each of the 15 phylogenomic datasets. Genes yielding irreproducible phylogenies are colored in red and genes yielding reproducible phylogenies are colored in yellow when using IQ-TREE; genes yielding irreproducible phylogenies are colored in green and genes yielding reproducible phylogenies are colored in blue when using RAxML-NG. Detailed values are given in Supplementary Data 2.

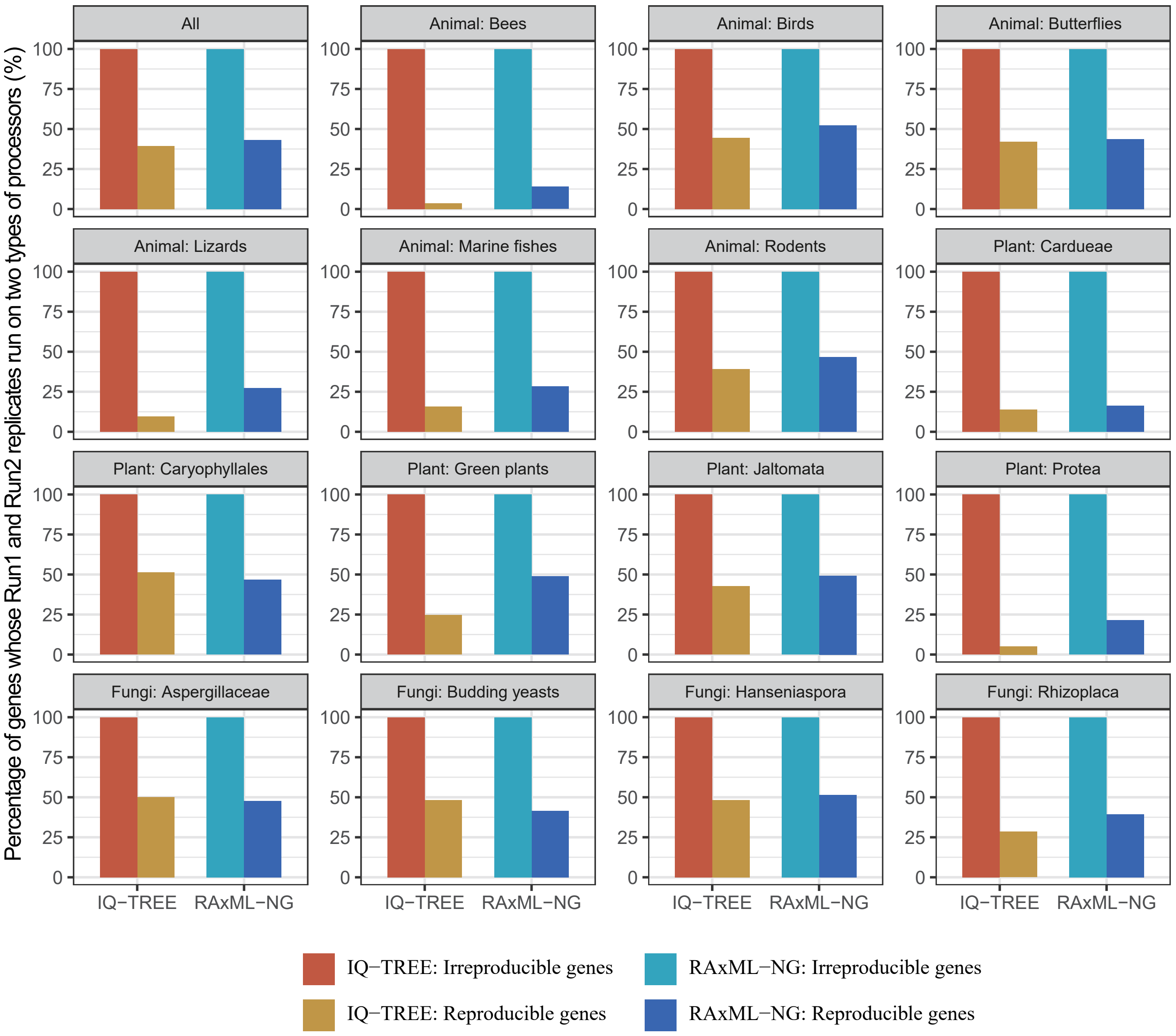

Supplementary Figure 11. Percentage of genes whose Run1 and Run2 replicates run on two types of processors. ML programs can automatically detect the optimal vector instructions. Two replicates (Run1 and Run2) for a given gene may be executed on two different types of processors in a supercomputing cluster. Bar plot at upper left is based on all 19,414 genes from 15 phylogenomic datasets. All other bar plots show the results for each of the 15 phylogenomic datasets. Genes yielding irreproducible phylogenies are colored in red and genes yielding reproducible phylogenies are colored in yellow when using IQ-TREE; genes yielding irreproducible phylogenies are colored in green and genes yielding reproducible phylogenies are colored in blue when using RAxML-NG. Detailed values are given in Supplementary Data 2.

Supplementary Figure 12. Coalescence-based ASTRAL trees for 15 phylogenomic datasets. For each of 15 datasets, we inferred four sets (iqtree_run1, iqtree_run2, raxml_ng_run1, and raxml_ng_run2) of individual gene trees using IQ-TREE and RAxML-NG and then built coalescent-based species trees with ASTRAL-III. The reliability of each internal branch was evaluated using local posterior probability (LPP). We visualized phylogenetic trees using the R package ggtree. Only support values smaller than 100% are shown for all species phylogenies (see command lines for inferring coalescence-based ASTRAL tree in the Supplementary Note 1). Red nodes indicate species relationships that differ between the species phylogeny inferred from Run1 trees and the species phylogeny inferred from Run2 trees. We found that 9 / 15 phylogenomic datasets produced topologically different coalescent-based species phylogenies from the Run1 and Run2 sets of individual gene trees, with the percentage of conflicting bipartitions ranging from 0.68% to 14.52%.

- ASTRAL tree (iqtree_run1_ASTRAL) at the **upper left** is the coalescence-based species phylogeny inferred from all of gene trees (run1) with IQ-TREE.
- ASTRAL tree (iqtree_run2_ASTRAL) at the **upper right** is the coalescence-based species phylogeny inferred from all of gene trees (run2) with IQ-TREE.
- ASTRAL tree (raxml_ng_run1_ASTRAL) at the **bottom left** is the coalescence-based species phylogeny inferred from all of gene trees (run1) with RAxML-NG
- ASTRAL tree (raxml_ng _run2_ASTRAL) at the **bottom right** is the coalescence-based species phylogeny inferred from all of gene trees (run2) with RAxML-NG.

To see how single gene trees affected the downstream analysis, we compared ASTRAL trees between run1 and run2 and colored the conflicting internal branches in red.

**Order**	**Dataset**	**Study**	**Program**	**Conflicting bipartitions (%)** [a]	**Highly conflicting bipartitions (%)** [b]
1	Animal: Bees	Bees	IQ-TREE	8.5561497	1.6042781
1	Animal: Bees	Bees	RAxML-NG	10.160428	3.4759358
2	Animal: Birds	Birds	IQ-TREE	1.5228426	0
2	Animal: Birds	Birds	RAxML-NG	1.5228426	0
3	Animal: Butterflies	Butterflys	IQ-TREE	4.4117647	1.2254902
3	Animal: Butterflies	Butterflys	RAxML-NG	1.9607843	0
4	Animal: Lizards	Lizards	IQ-TREE	3.8461538	0
4	Animal: Lizards	Lizards	RAxML-NG	0	0
5	Animal: Marine fishes	Marine_fishes	IQ-TREE	4.2735043	0
5	Animal: Marine fishes	Marine_fishes	RAxML-NG	6.8376068	0
6	Animal: Rodents	Rodents	IQ-TREE	0	0
6	Animal: Rodents	Rodents	RAxML-NG	0	0
7	Plant: Cardueae	Cardueae	IQ-TREE	4.8780488	1.2195122
7	Plant: Cardueae	Cardueae	RAxML-NG	3.6585366	0
8	Plant: Caryophyllales	Caryophyllales	IQ-TREE	0	0
8	Plant: Caryophyllales	Caryophyllales	RAxML-NG	0	0
9	Plant: Green plants	Green_plants	IQ-TREE	1.7879949	0.0425713
9	Plant: Green plants	Green_plants	RAxML-NG	0.6808511	0.1276596
10	Plant: Jaltomata	Jaltomata	IQ-TREE	0	0
10	Plant: Jaltomata	Jaltomata	RAxML-NG	0	0
11	Plant: Protea	Protea	IQ-TREE	14.516129	5.6451613
11	Plant: Protea	Protea	RAxML-NG	8.0645161	2.4193548
12	Fungi: Aspergillaceae	Aspergillaceae	IQ-TREE	0	0
12	Fungi: Aspergillaceae	Aspergillaceae	RAxML-NG	0	0
13	Fungi: Budding yeasts	Budding_yeasts	IQ-TREE	0	0
13	Fungi: Budding yeasts	Budding_yeasts	RAxML-NG	0	0
14	Fungi: Hanseniaspora	Hanseniaspora	IQ-TREE	0	0
14	Fungi: Hanseniaspora	Hanseniaspora	RAxML-NG	0	0
15	Fungi: Rhizoplaca	Rhizoplaca	IQ-TREE	10.714286	0
15	Fungi: Rhizoplaca	Rhizoplaca	RAxML-NG	3.5714286	0

[a] Percentage of conflicting bipartitions between run1 ASTRAL and run2 ASTRAL

[b] Percentage of highly conflicting bipartitions with LPP ≥ 90% between run1 ASTRAL and run2 ASTRAL

Bees_iqtree_run1_ASTRAL

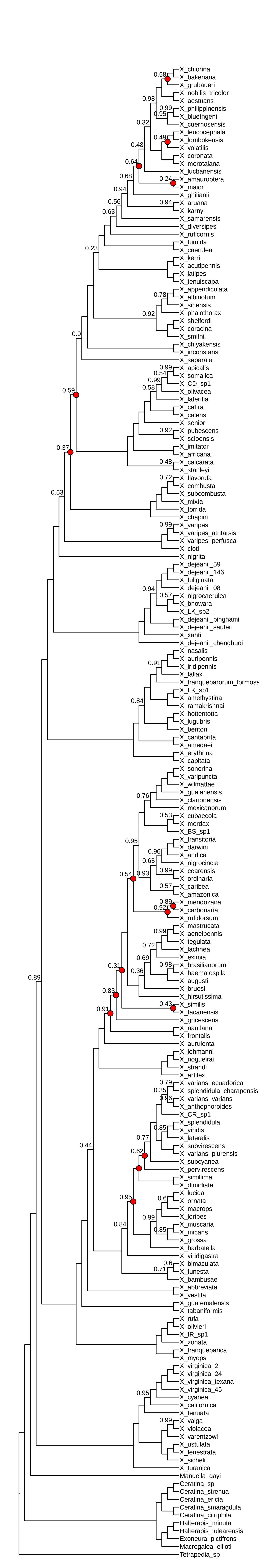

Bees_iqtree_run2_ASTRAL

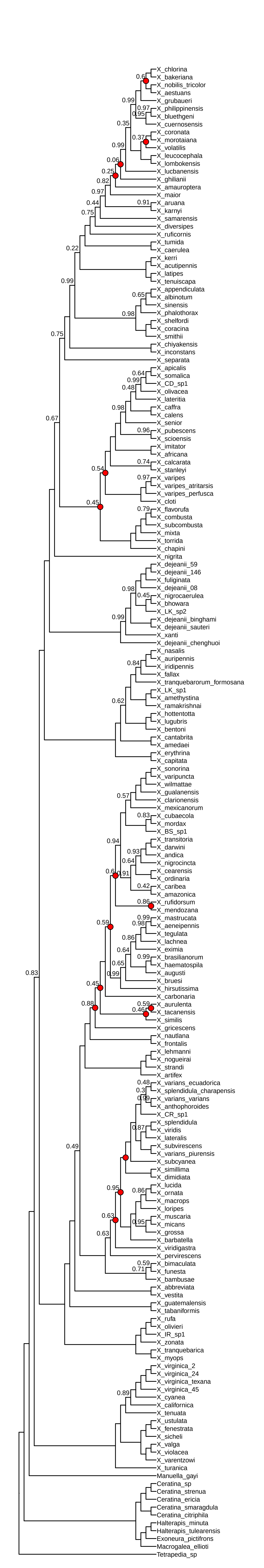

Bees_raxml_ng_run1_ASTRAL

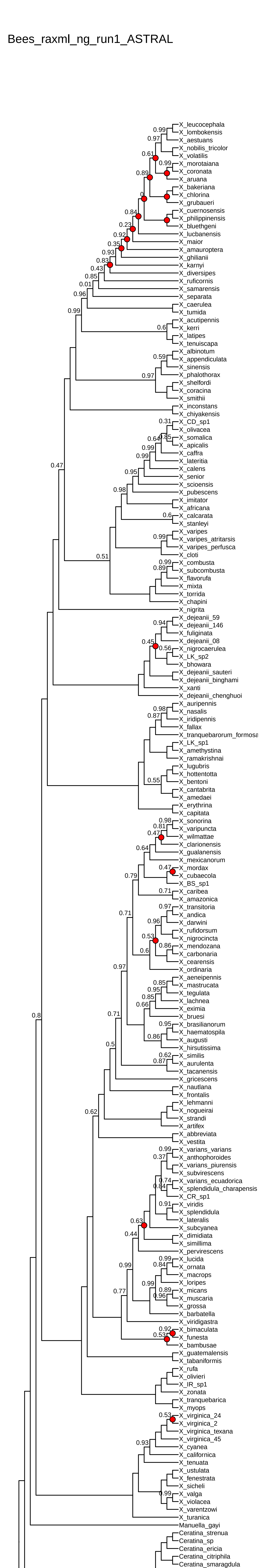

Bees_raxml_ng_run2_ASTRAL

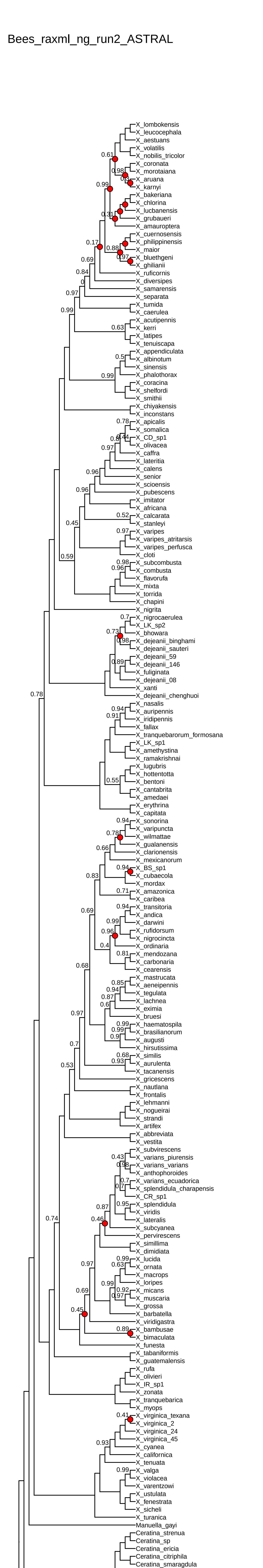

Birds_iqtree_run1_ASTRAL

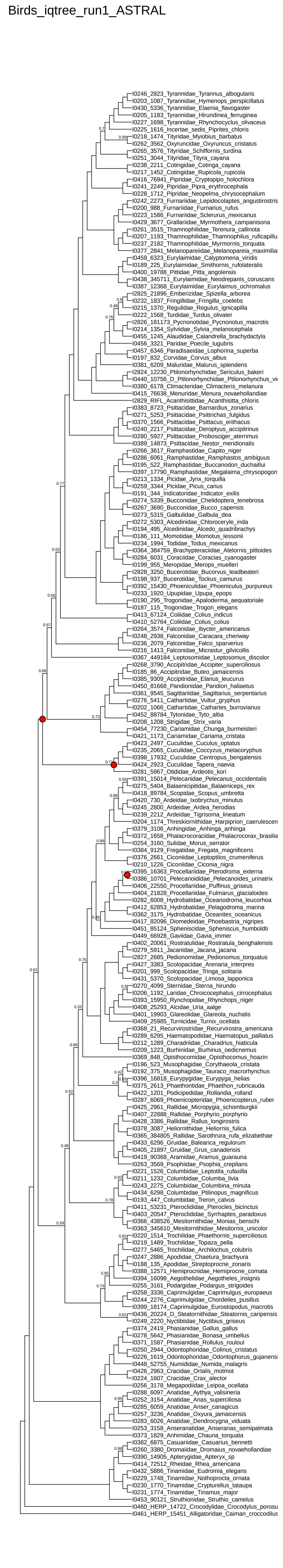

Birds_iqtree_run2_ASTRAL

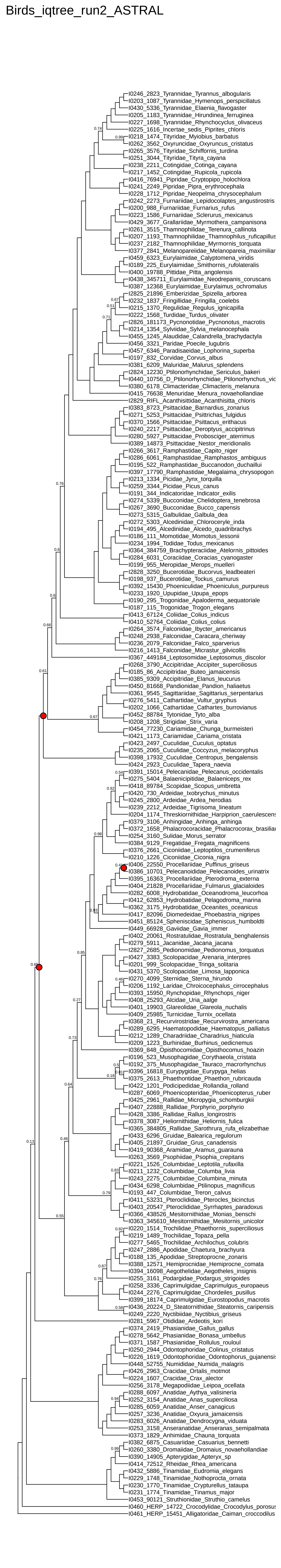

Birds_raxml_ng_run1_ASTRAL

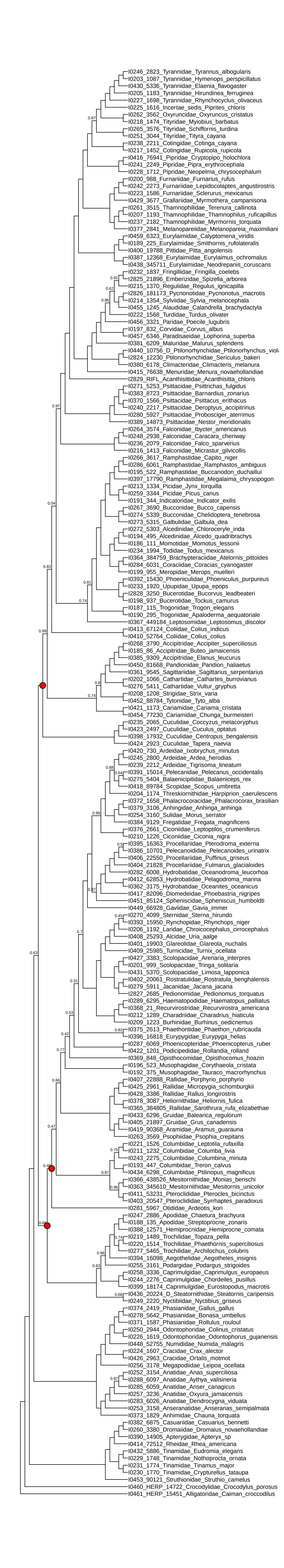

Birds_raxml_ng_run2_ASTRAL

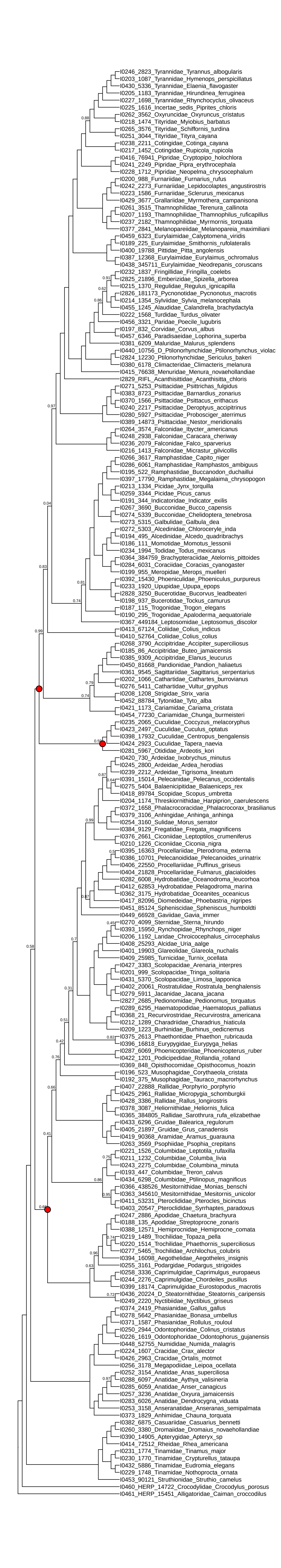

Butterflys_iqtree_run1_ASTRAL

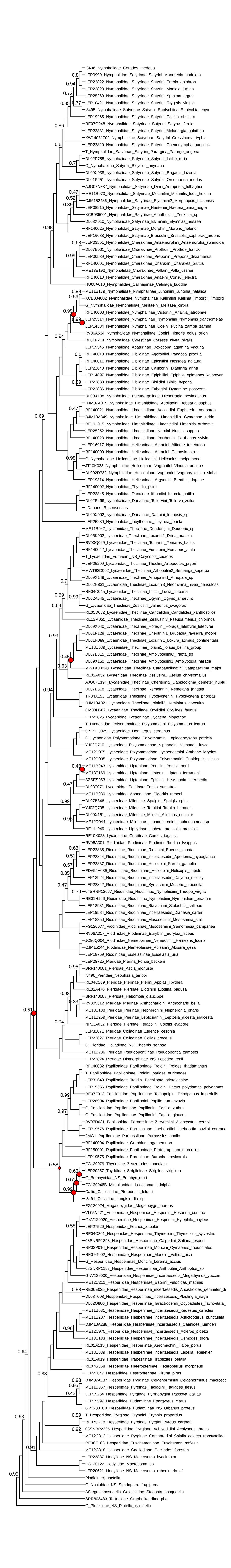

Butterflys_iqtree_run2_ASTRAL

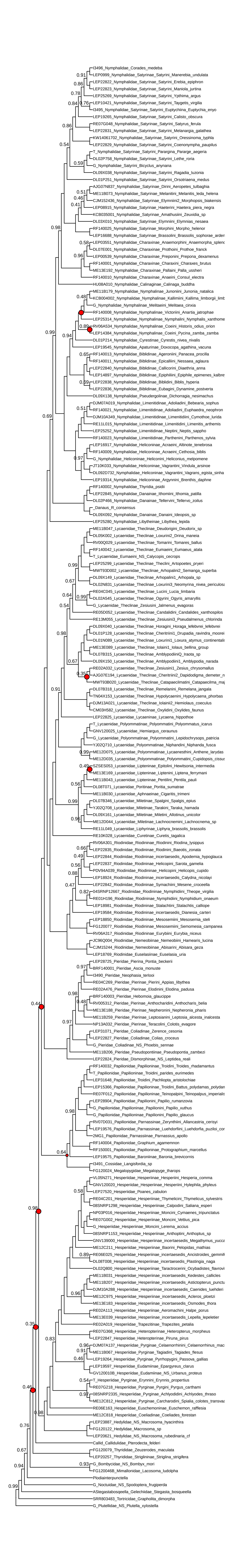

Butterflys_raxml_ng_run1_ASTRAL

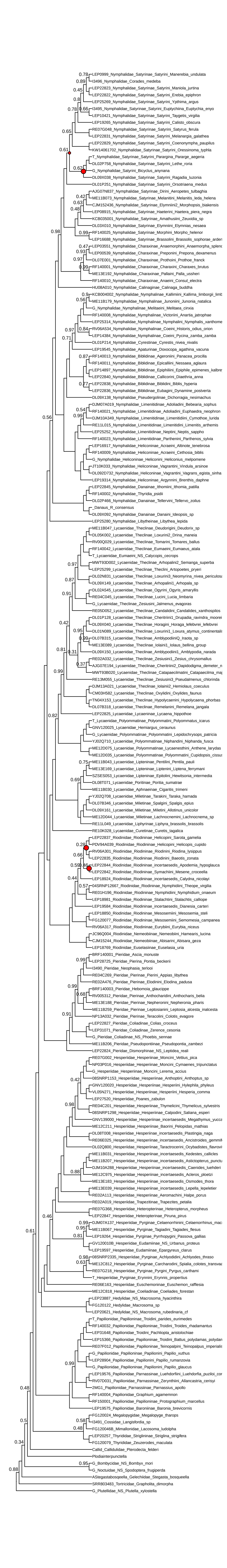

Butterflys_raxml_ng_run2_ASTRAL

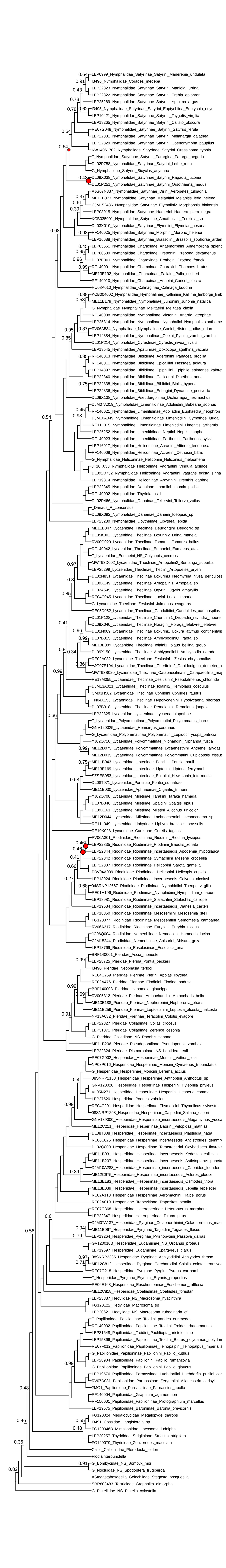

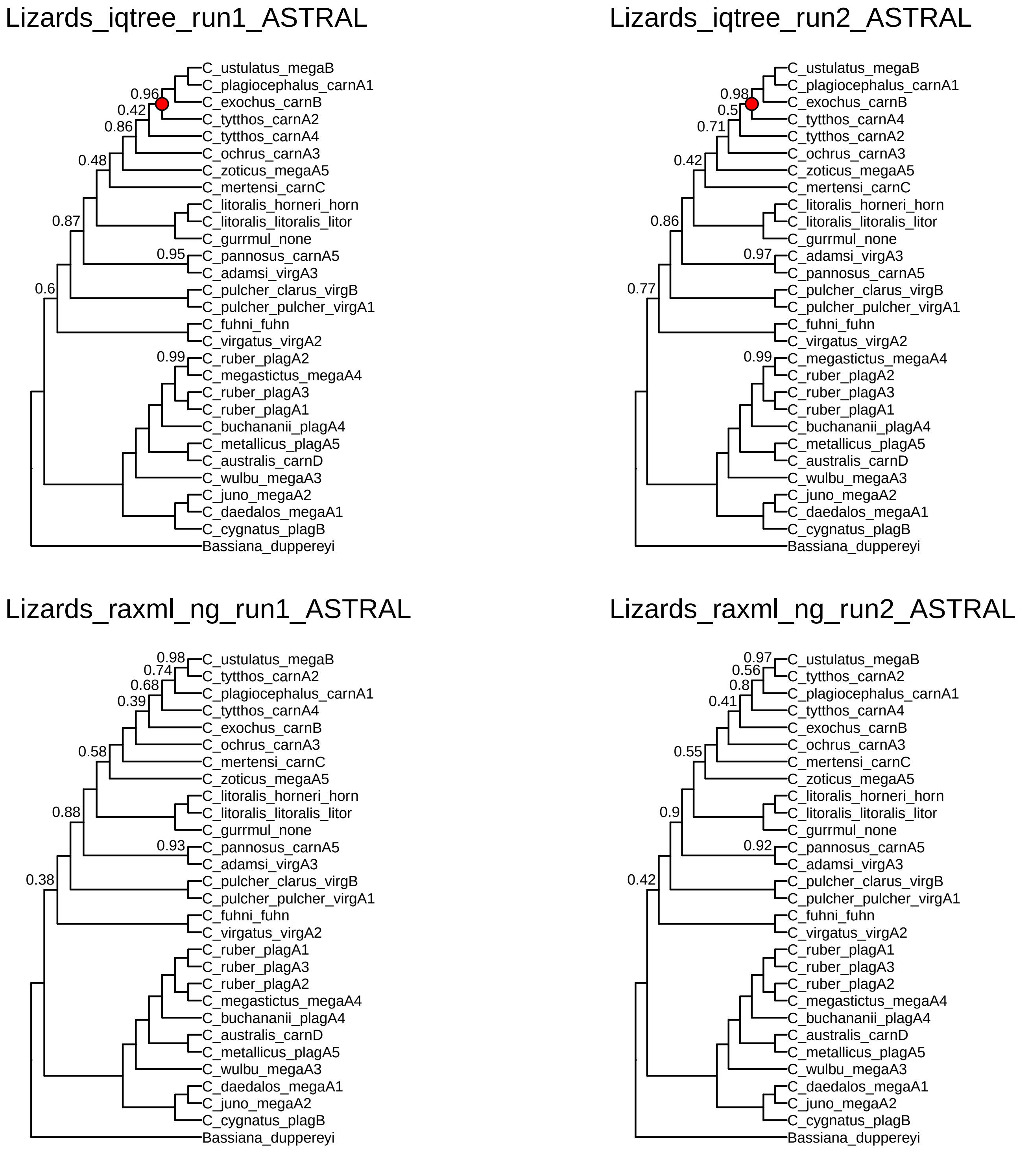
Lizards_iqtree_run1_ASTRAL
0.96
0.42
0.86
0.48
0.87
0.95
0.6
0.99
C_ustulatus_megaB
C_plagiocephalus_carnA1
C_exochus_carnB
C_tytthos_carnA2
C_tytthos_carnA4
C_ochrus_carnA3
C_zoticus_megaA5
C_mertensi_carnC
C_litoralis_horneri_horn
C_litoralis_litoralis_litor
C_gurrmul_none
C_pannosus_carnA5
C_adamsi_virgA3
C_pulcher_clarus_virgB
C_pulcher_pulcher_virgA1
C_fuhni_fuhn
C_virgatus_virgA2
C_ruber_plagA2
C_megastictus_megaA4
C_ruber_plagA3
C_ruber_plagA1
C_buchananii_plagA4
C_metallicus_plagA5
C_australis_carnD
C_wulbu_megaA3
C_juno_megaA2
C_daedalos_megaA1
C_cygnatus_plagB
Bassiana_duppereyi
Lizards_iqtree_run2_ASTRAL
0.98
0.5
0.71
0.42
0.86
0.97
0.77
0.99
C_ustulatus_megaB
C_plagiocephalus_carnA1
C_exochus_carnB
C_tytthos_carnA4
C_tytthos_carnA2
C_ochrus_carnA3
C_zoticus_megaA5
C_mertensi_carnC
C_litoralis_horneri_horn
C_litoralis_litoralis_litor
C_gurrmul_none
C_adamsi_virgA3
C_pannosus_carnA5
C_pulcher_clarus_virgB
C_pulcher_pulcher_virgA1
C_fuhni_fuhn
C_virgatus_virgA2
C_megastictus_megaA4
C_ruber_plagA2
C_ruber_plagA3
C_ruber_plagA1
C_buchananii_plagA4
C_metallicus_plagA5
C_australis_carnD
C_wulbu_megaA3
C_juno_megaA2
C_daedalos_megaA1
C_cygnatus_plagB
Bassiana_duppereyi
Lizards_raxml_ng_run1_ASTRAL
0.98
0.74
0.68
0.39
0.58
0.88
0.93
0.38
C_ustulatus_megaB
C_tytthos_carnA2
C_plagiocephalus_carnA1
C_tytthos_carnA4
C_exochus_carnB
C_ochrus_carnA3
C_mertensi_carnC
C_zoticus_megaA5
C_litoralis_horneri_horn
C_litoralis_litoralis_litor
C_gurrmul_none
C_pannosus_carnA5
C_adamsi_virgA3
C_pulcher_clarus_virgB
C_pulcher_pulcher_virgA1
C_fuhni_fuhn
C_virgatus_virgA2
C_ruber_plagA1
C_ruber_plagA3
C_ruber_plagA2
C_megastictus_megaA4
C_buchananii_plagA4
C_australis_carnD
C_metallicus_plagA5
C_wulbu_megaA3
C_daedalos_megaA1
C_juno_megaA2
C_cygnatus_plagB
Bassiana_duppereyi
Lizards_raxml_ng_run2_ASTRAL
0.97
0.56
0.8
0.41
0.55
0.9
0.92
0.42
C_ustulatus_megaB
C_tytthos_carnA2
C_plagiocephalus_carnA1
C_tytthos_carnA4
C_exochus_carnB
C_ochrus_carnA3
C_mertensi_carnC
C_zoticus_megaA5
C_litoralis_horneri_horn
C_litoralis_litoralis_litor
C_gurrmul_none
C_pannosus_carnA5
C_adamsi_virgA3
C_pulcher_clarus_virgB
C_pulcher_pulcher_virgA1
C_fuhni_fuhn
C_virgatus_virgA2
C_ruber_plagA1
C_ruber_plagA3
C_ruber_plagA2
C_megastictus_megaA4
C_buchananii_plagA4
C_australis_carnD
C_metallicus_plagA5
C_wulbu_megaA3
C_daedalos_megaA1
C_juno_megaA2
C_cygnatus_plagB
Bassiana_duppereyi

Marine_fishes_iqtree_run1_ASTRAL

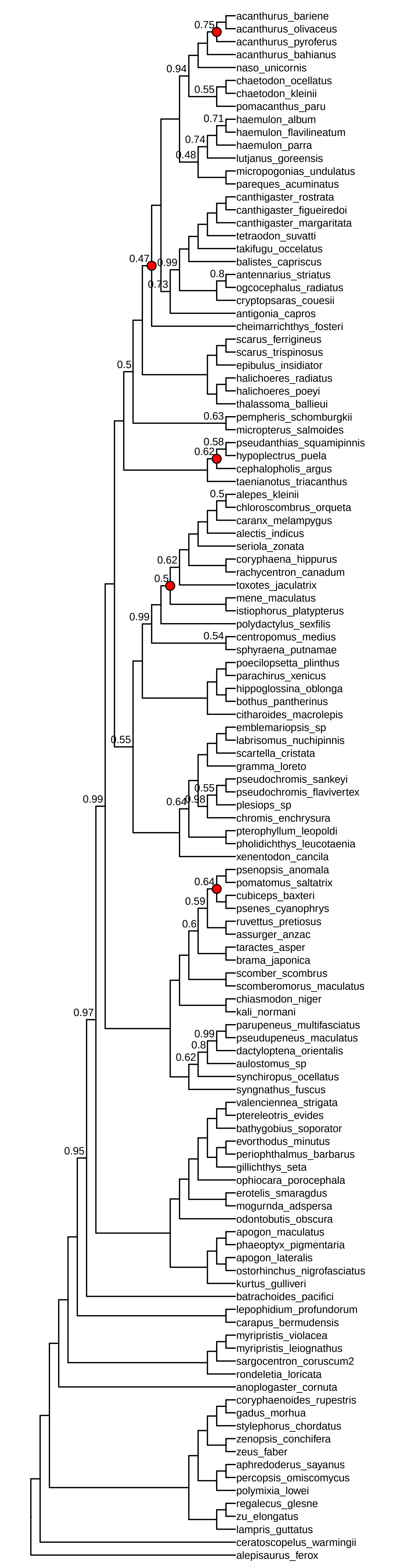

Marine_fishes_iqtree_run2_ASTRAL

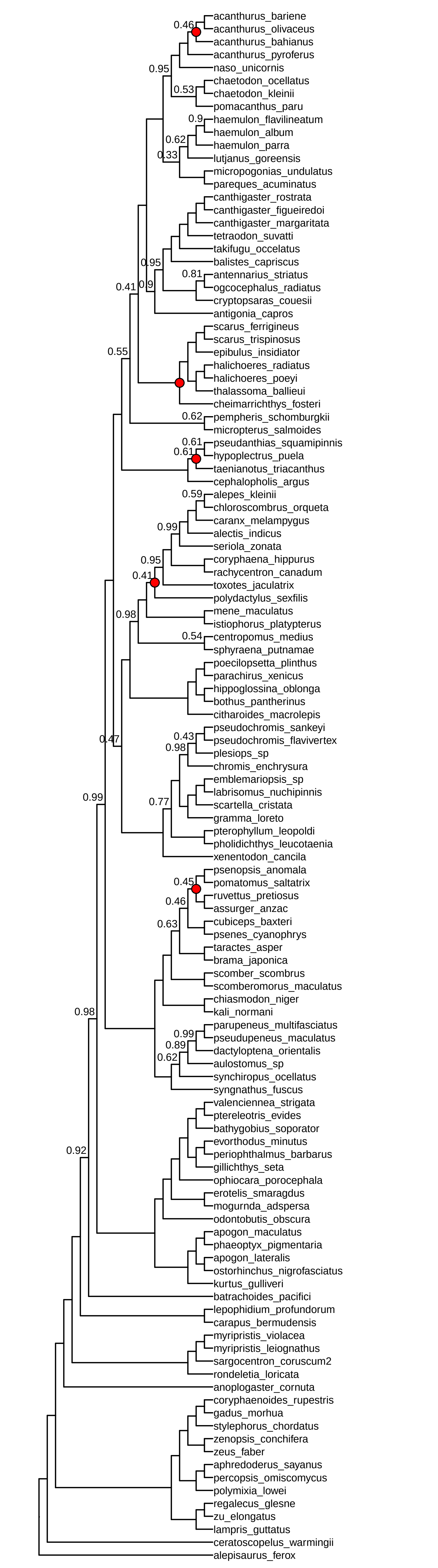

Marine_fishes_raxml_ng_run1_ASTRAL

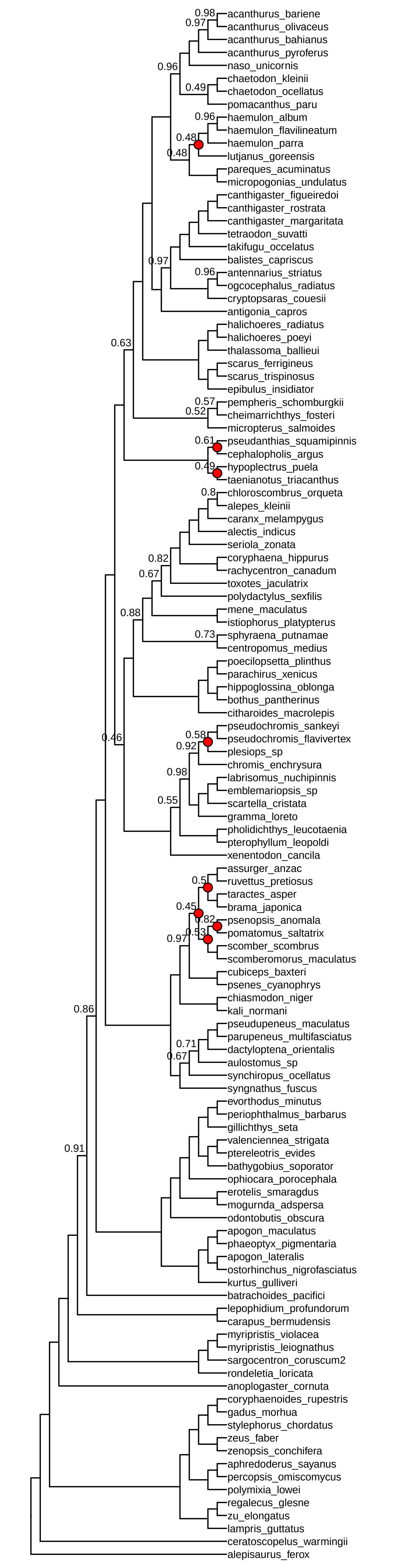

Marine_fishes_raxml_ng_run2_ASTRAL

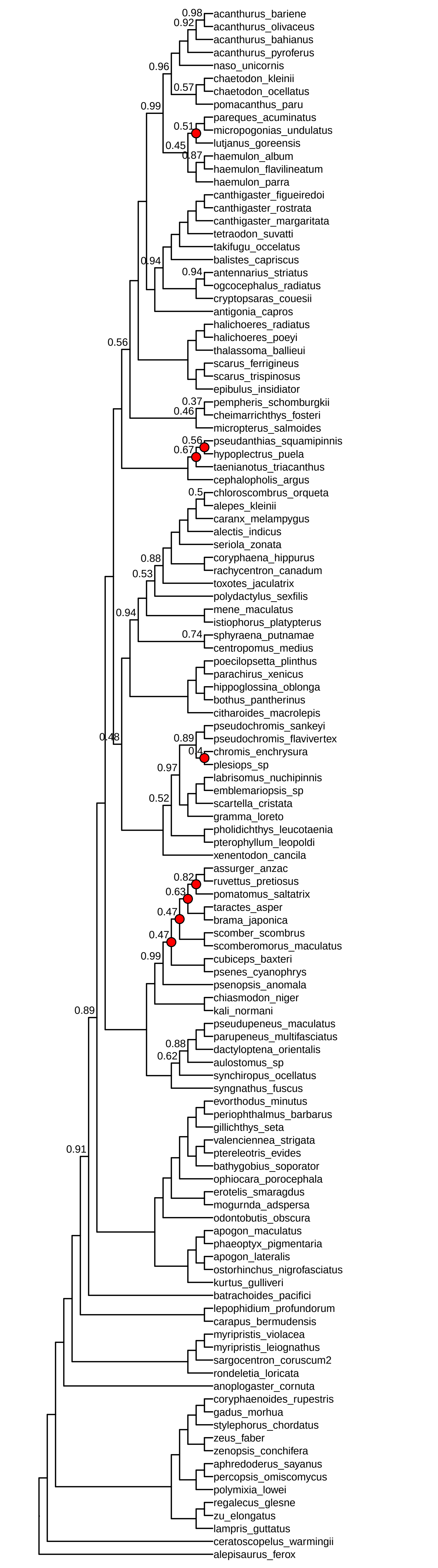

Rodents_iqtree_run1_ASTRAL

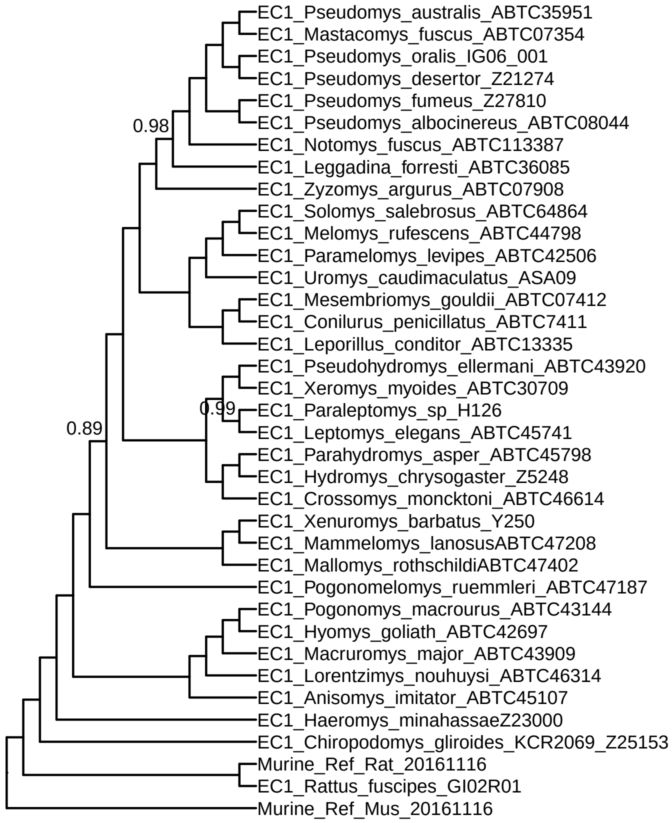

Rodents_iqtree_run2_ASTRAL

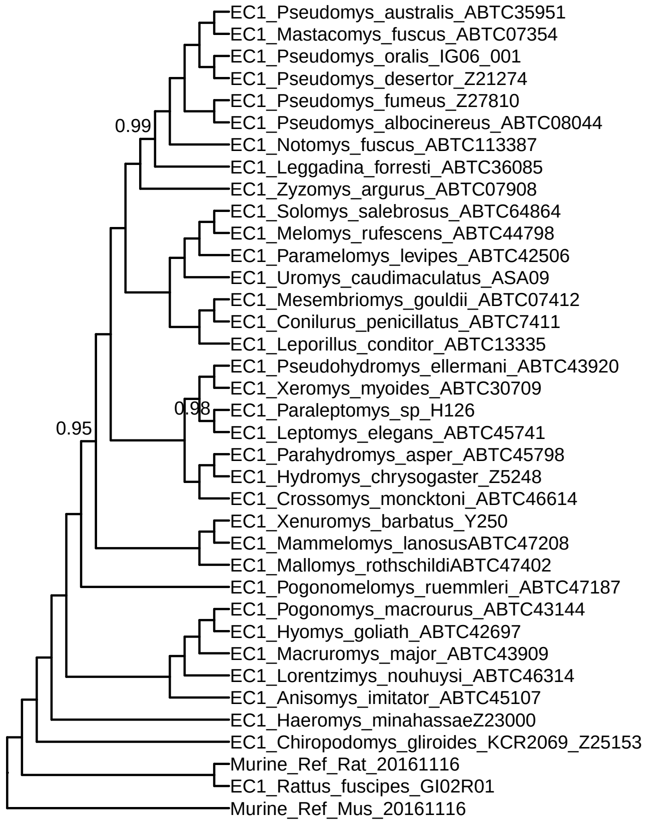

Rodents_raxml_ng_run1_ASTRAL

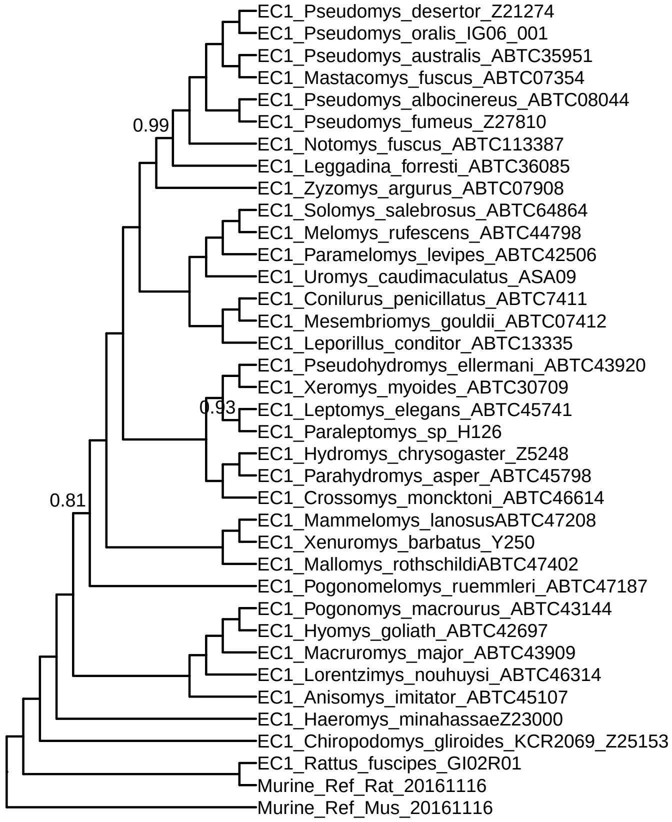

Rodents_raxml_ng_run2_ASTRAL

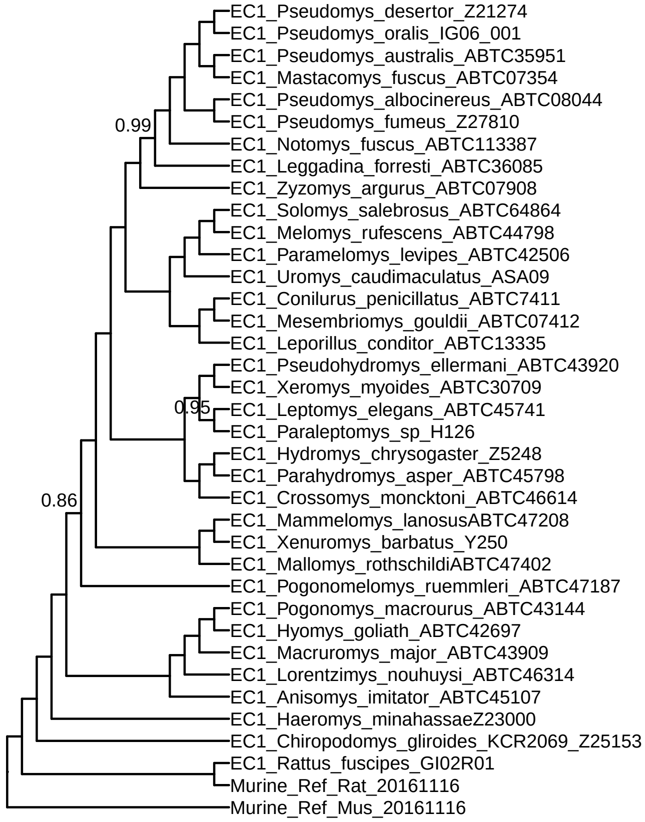

Cardueae_iqtree_run1_ASTRAL

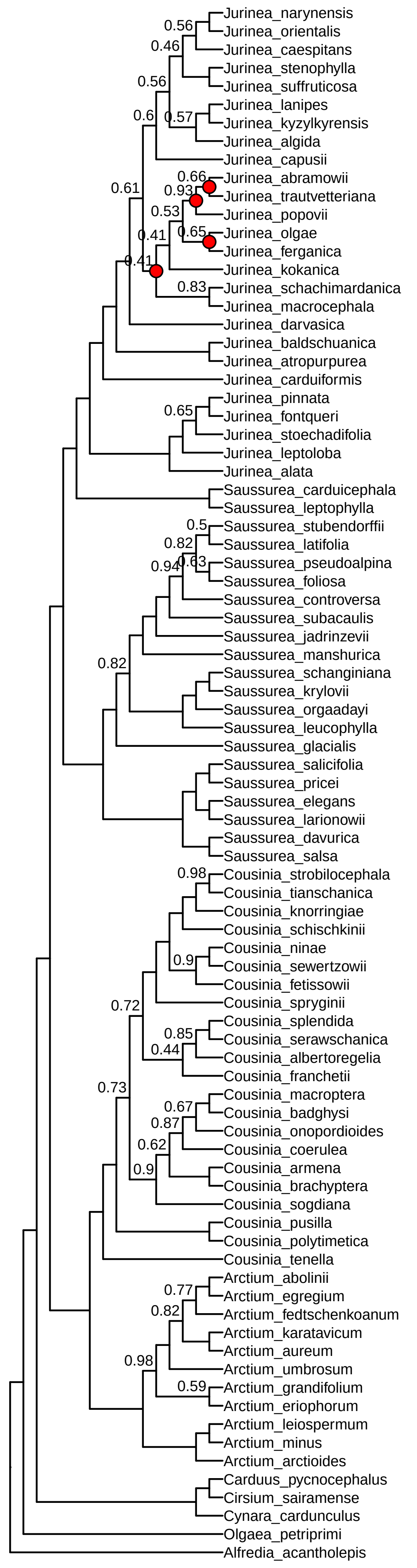

Cardueae_iqtree_run2_ASTRAL

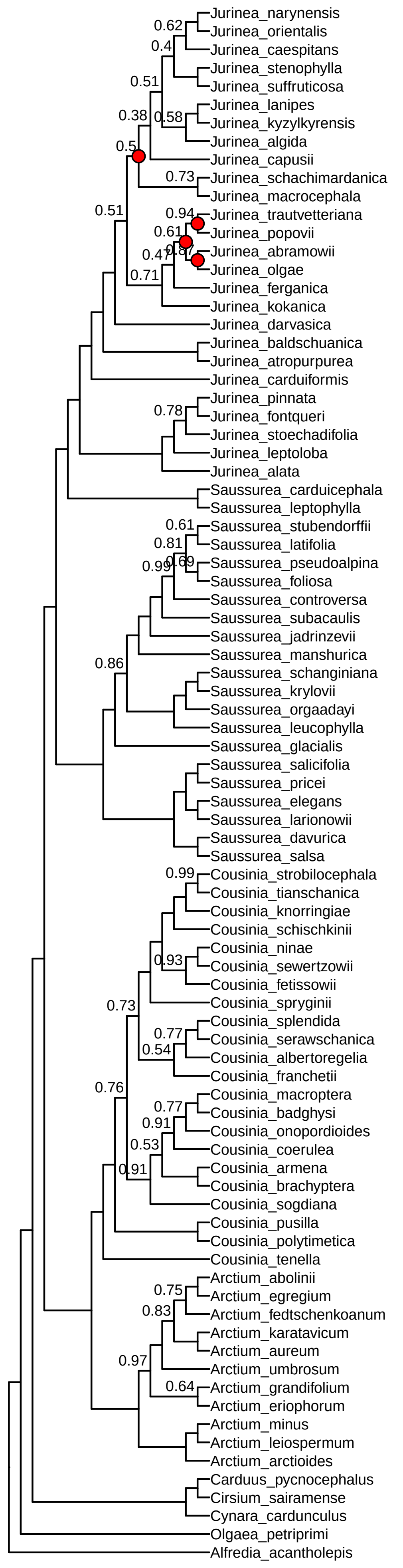

Cardueae_raxml_ng_run1_ASTRAL

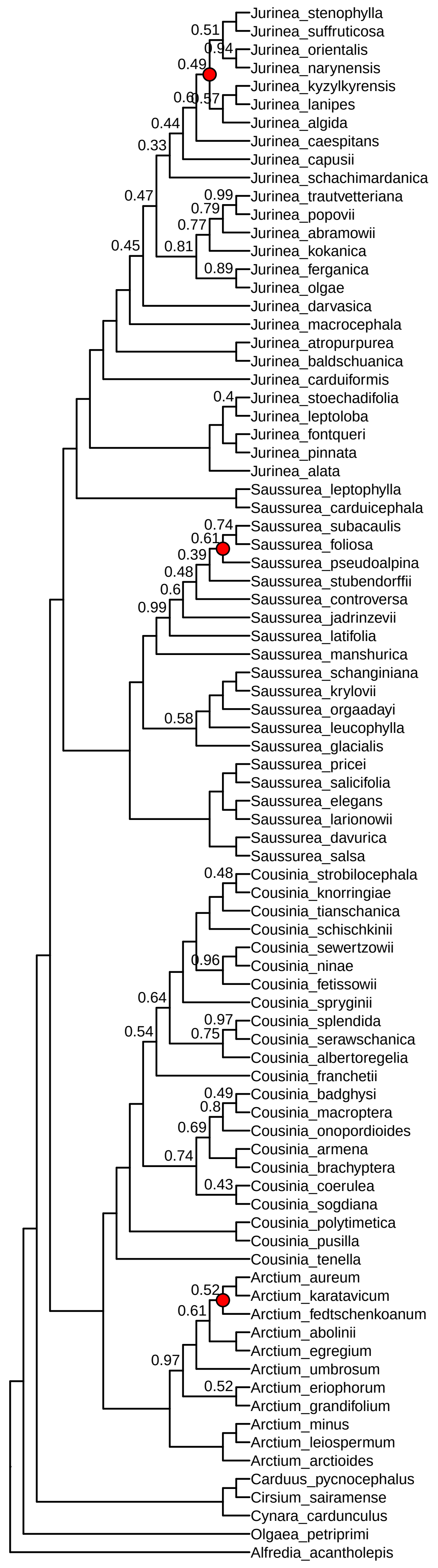

Cardueae_raxml_ng_run2_ASTRAL

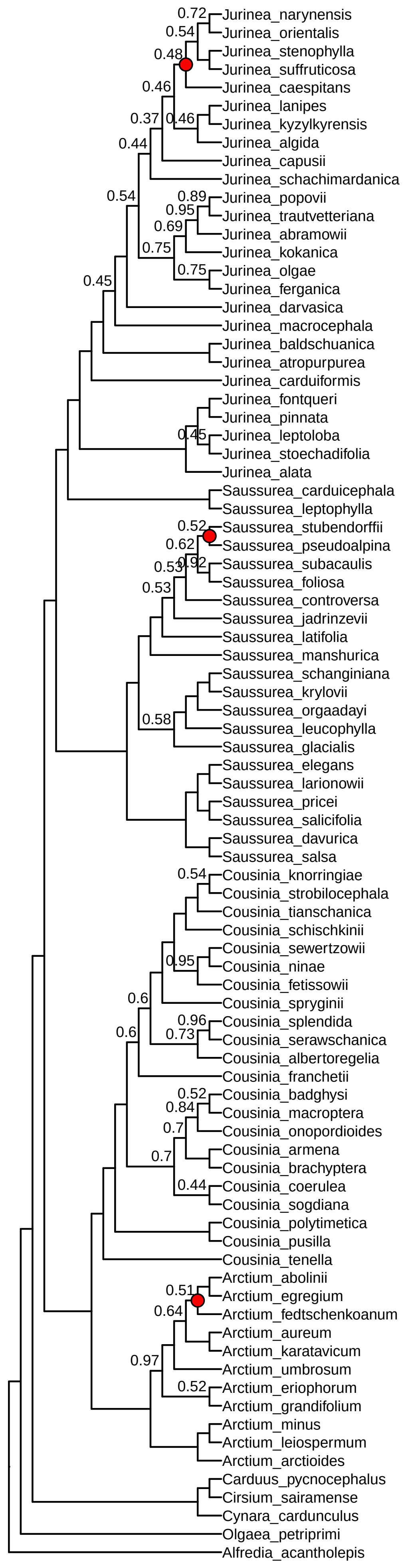

Caryophyllales_iqtree_run1_ASTRAL

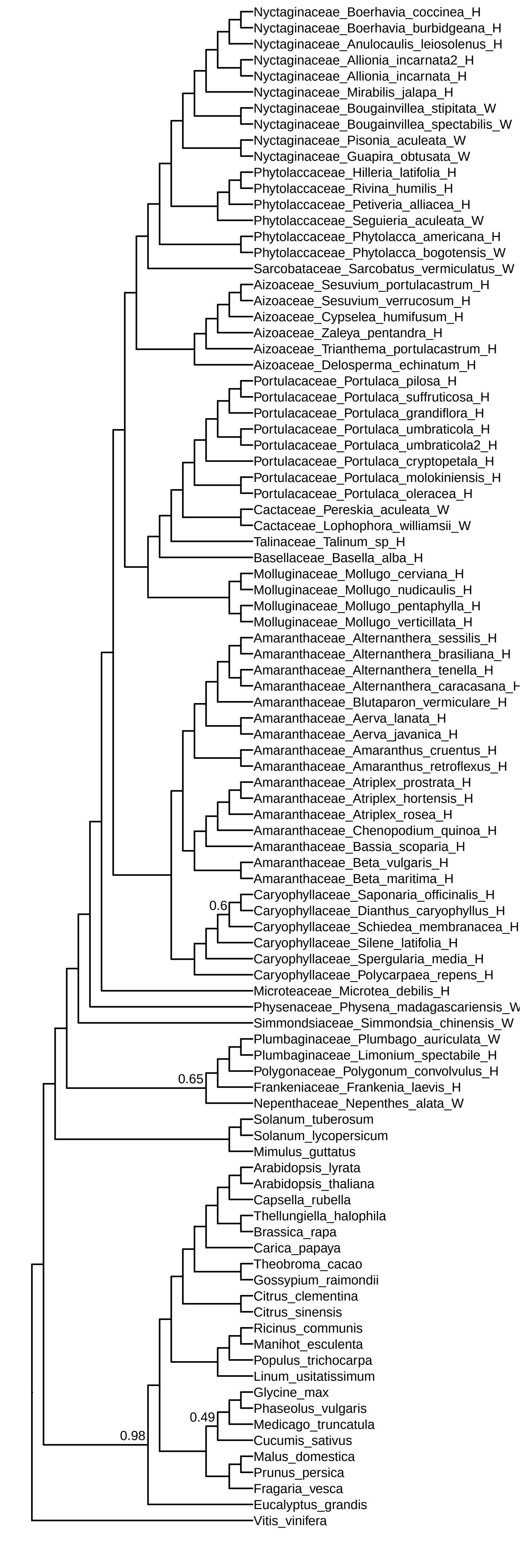

Caryophyllales_iqtree_run2_ASTRAL

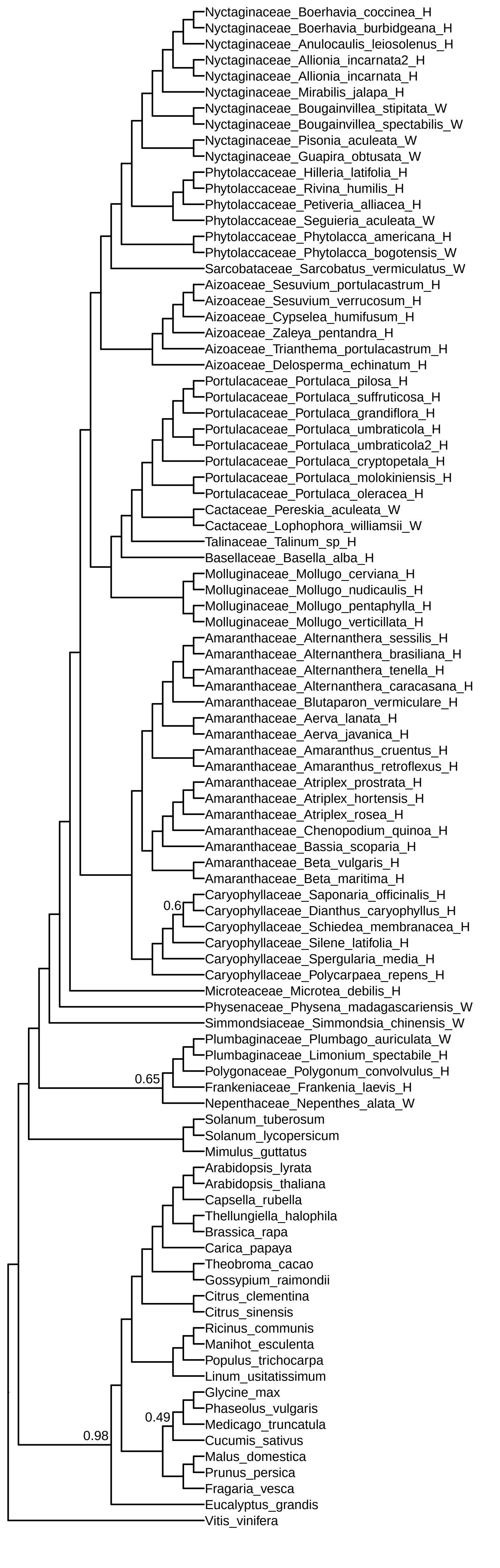

Caryophyllales_raxml_ng_run1_ASTRAL

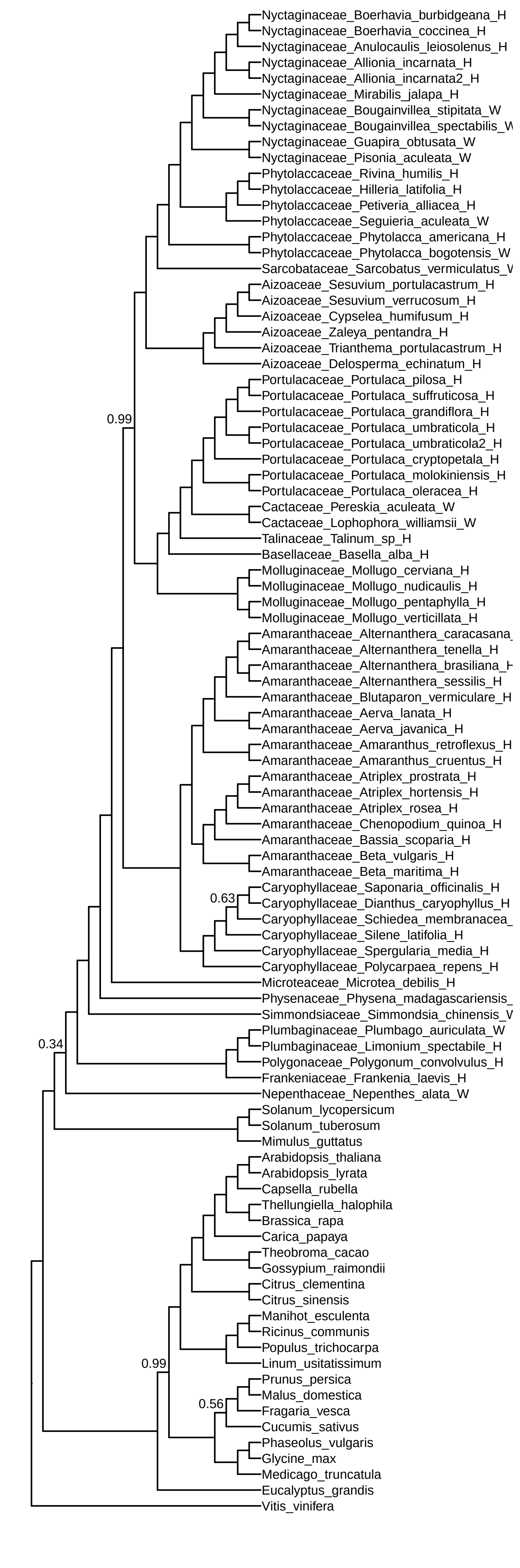

Caryophyllales_raxml_ng_run2_ASTRAL

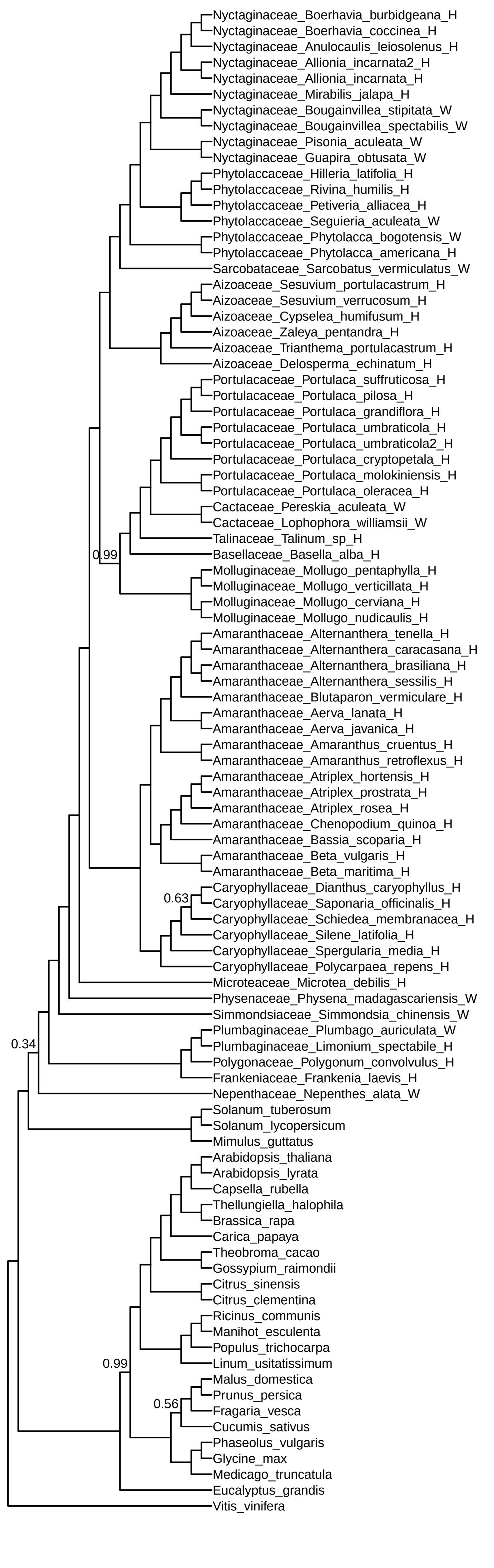

Green_plants_CHTC_2CPU_random_node_raxml_ng_run

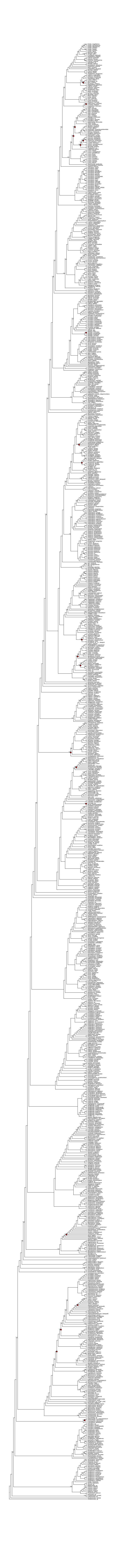

Green_plants_CHTC_2CPU_random_node_raxml_ng_run

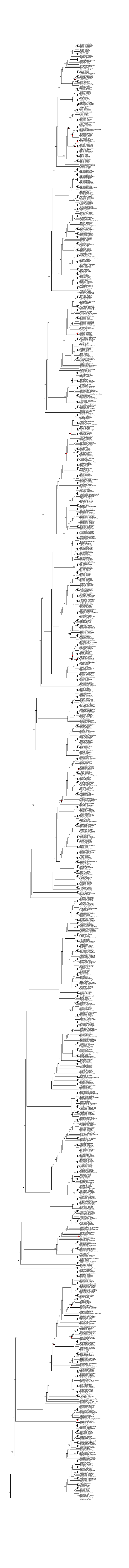

Green_plants_CHTC_2CPU_random_node_iqtree_run1

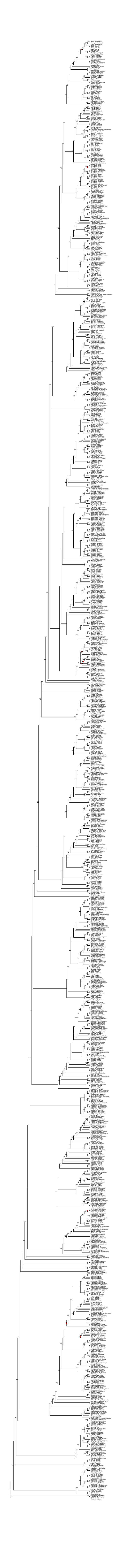

Green_plants_CHTC_2CPU_random_node_iqtree_run2

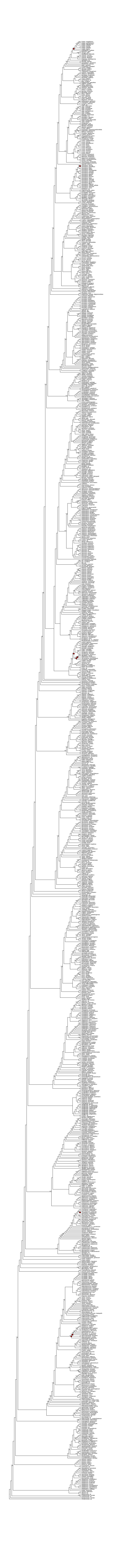

Jaltomata_iqtree_run1_ASTRAL

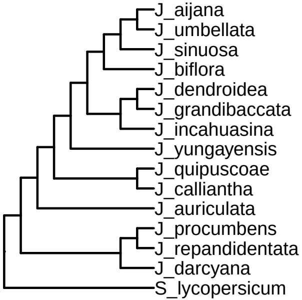

Jaltomata_iqtree_run2_ASTRAL

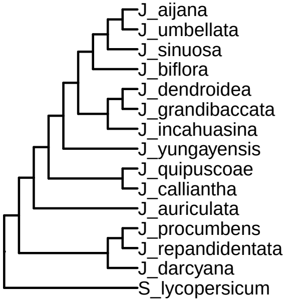

Jaltomata_raxml_ng_run1_ASTRAL

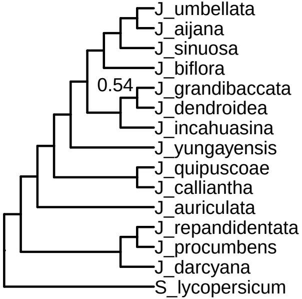

Jaltomata_raxml_ng_run2_ASTRAL

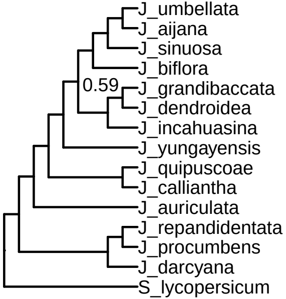

Protea_iqtree_run1_ASTRAL

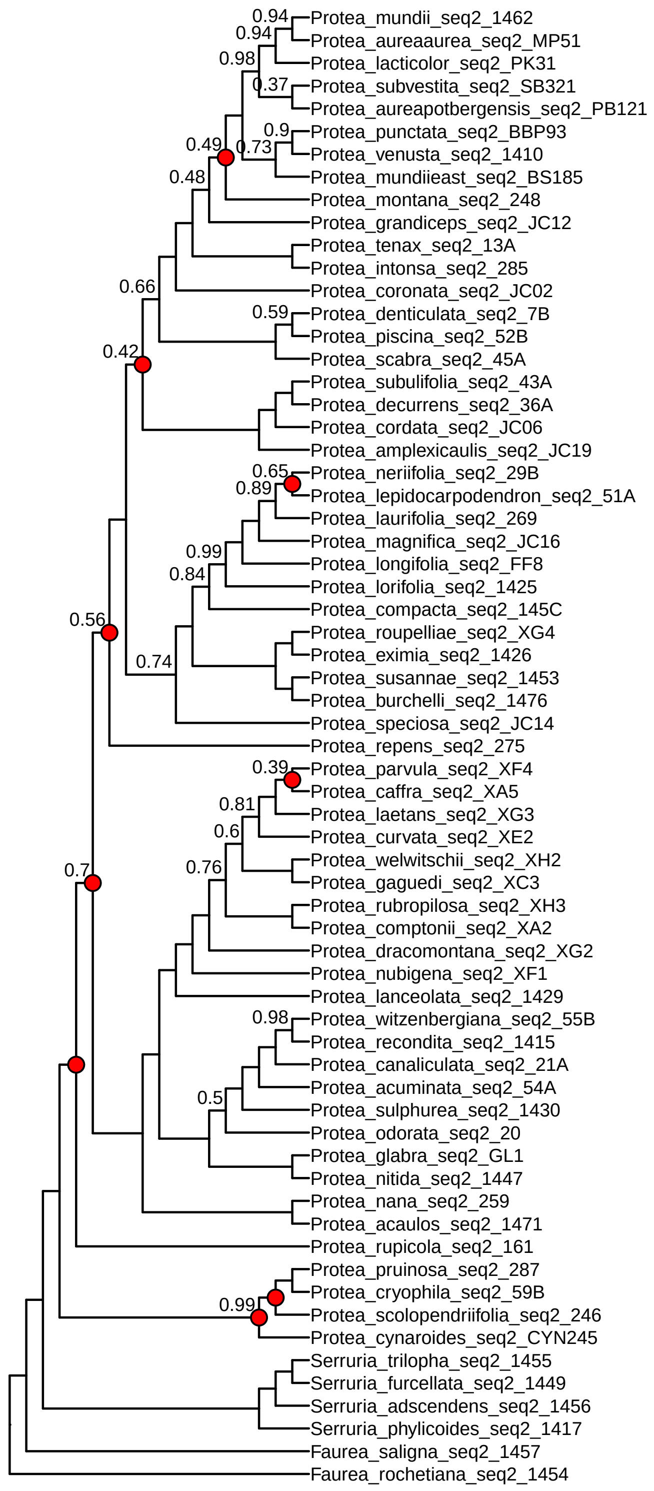

Protea_iqtree_run2_ASTRAL

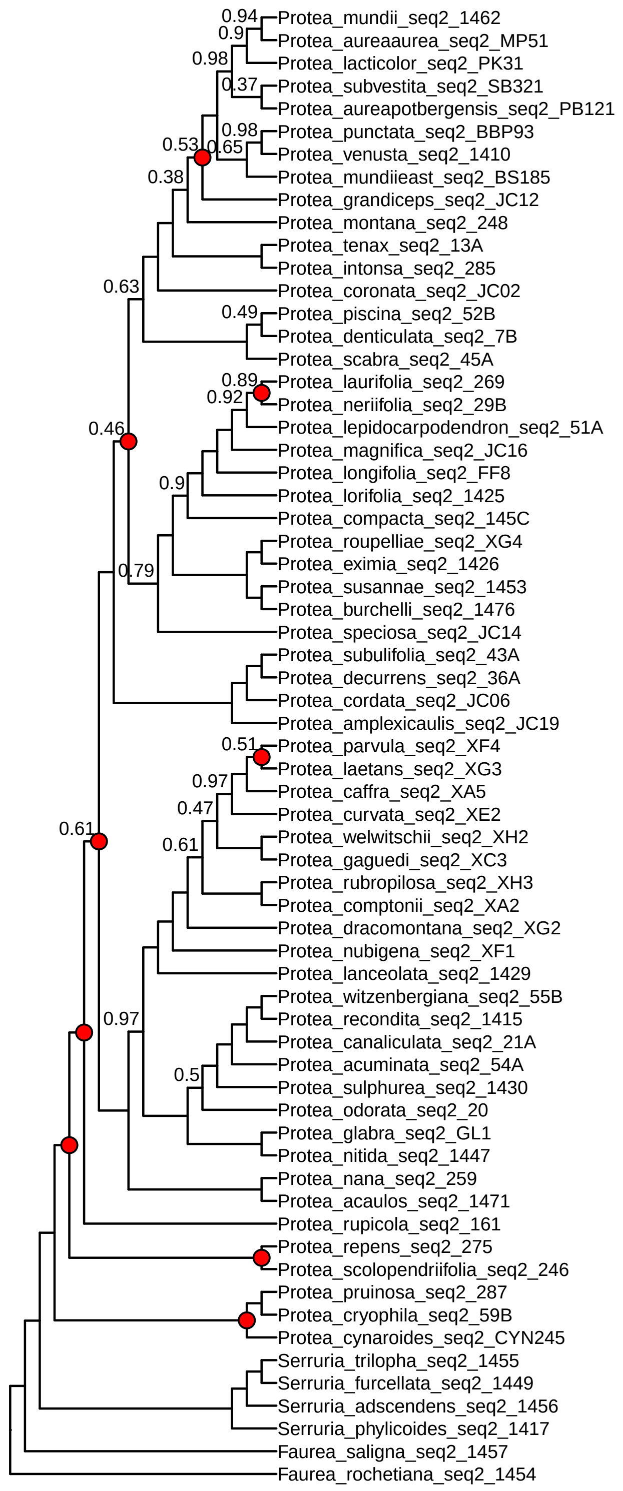

Protea_raxml_ng_run1_ASTRAL

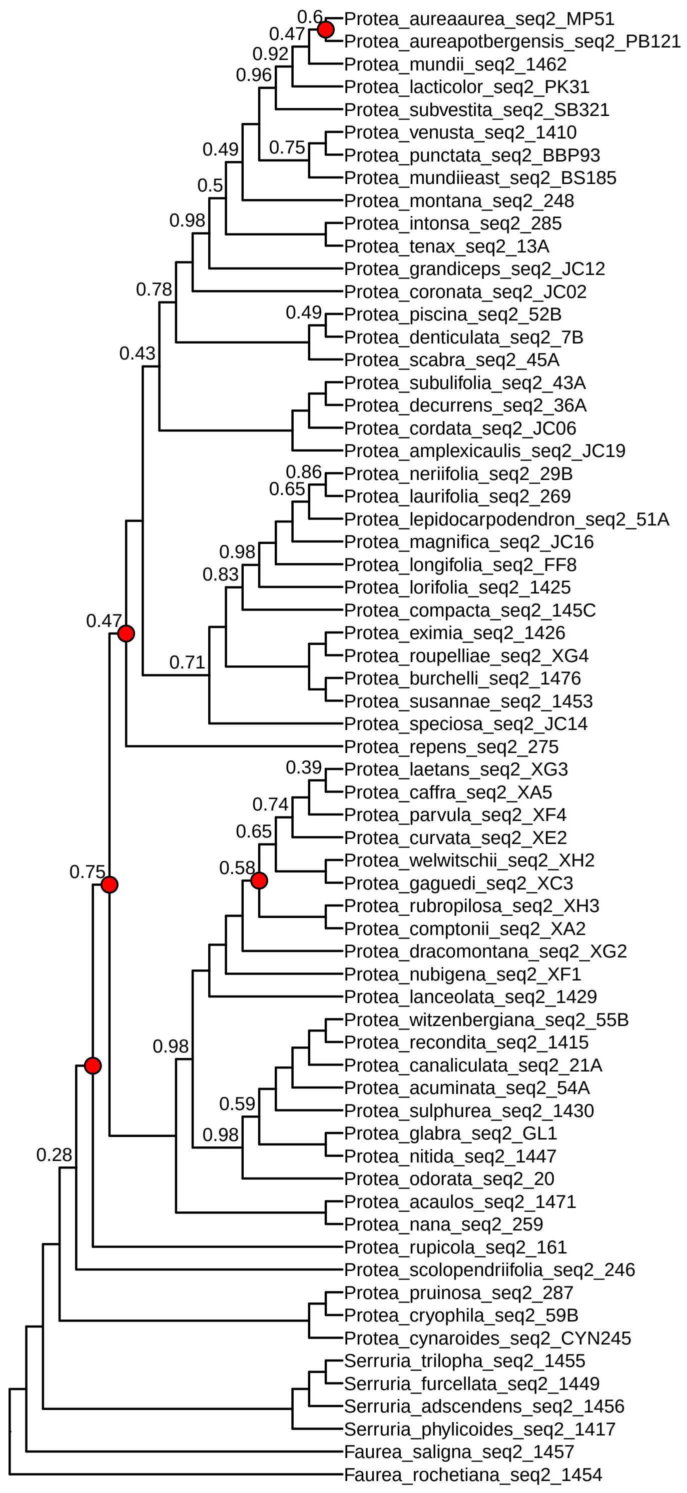

Protea_raxml_ng_run2_ASTRAL

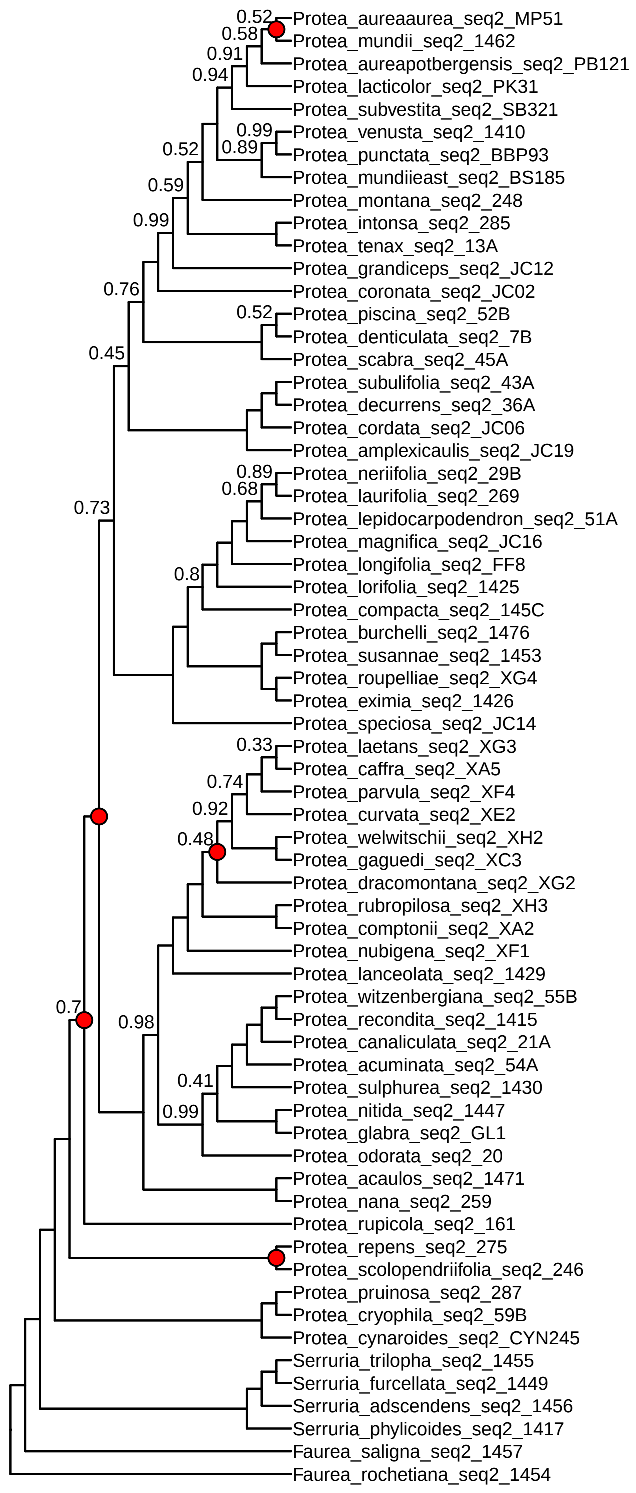

Aspergillaceae_iqtree_run1_ASTRAL

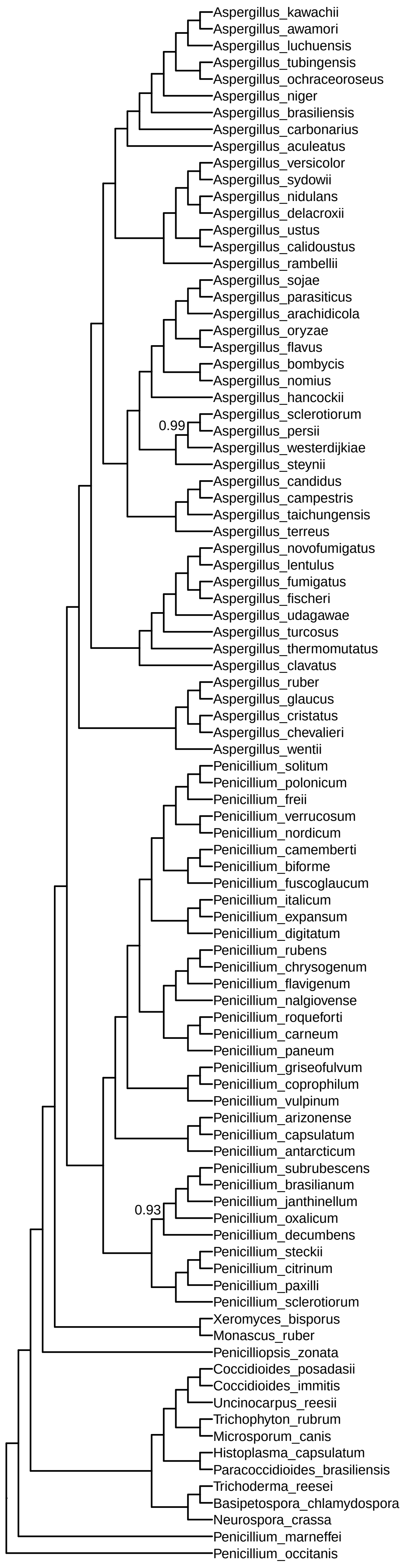

Aspergillaceae_iqtree_run2_ASTRAL

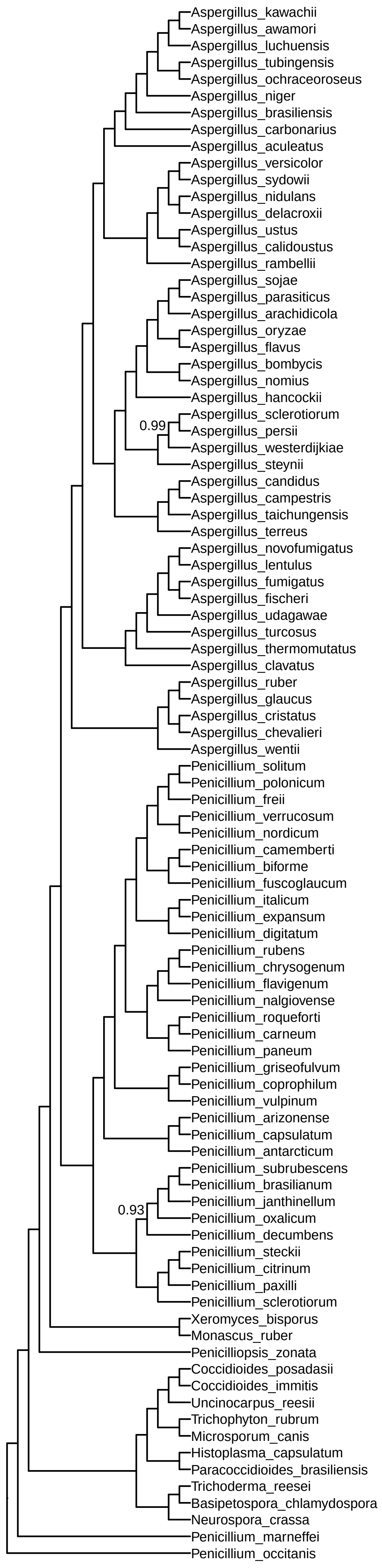

Aspergillaceae_raxml_ng_run1_ASTRAL

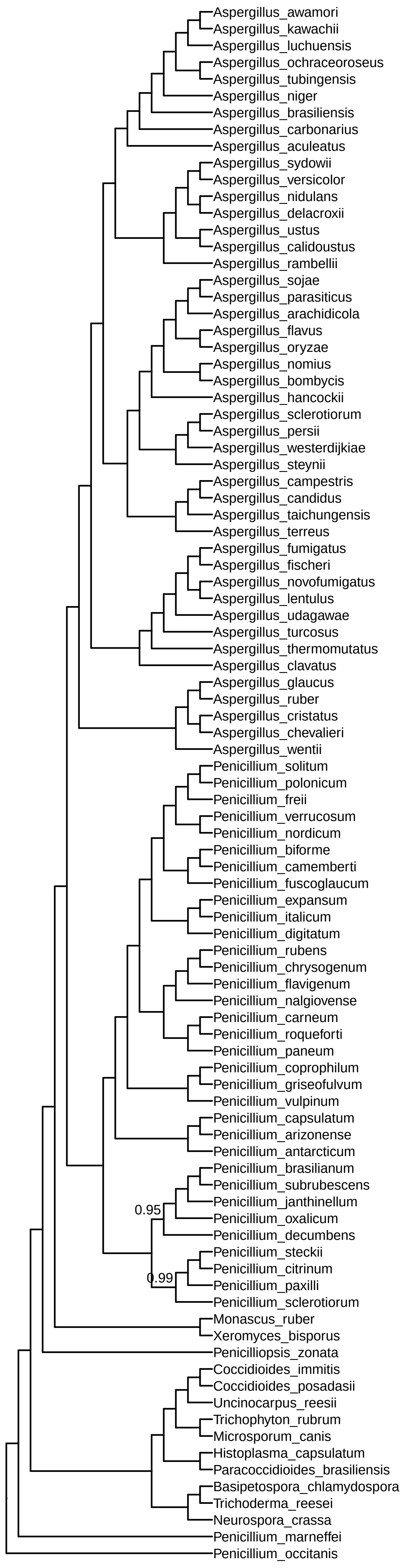

Aspergillaceae_raxml_ng_run2_ASTRAL

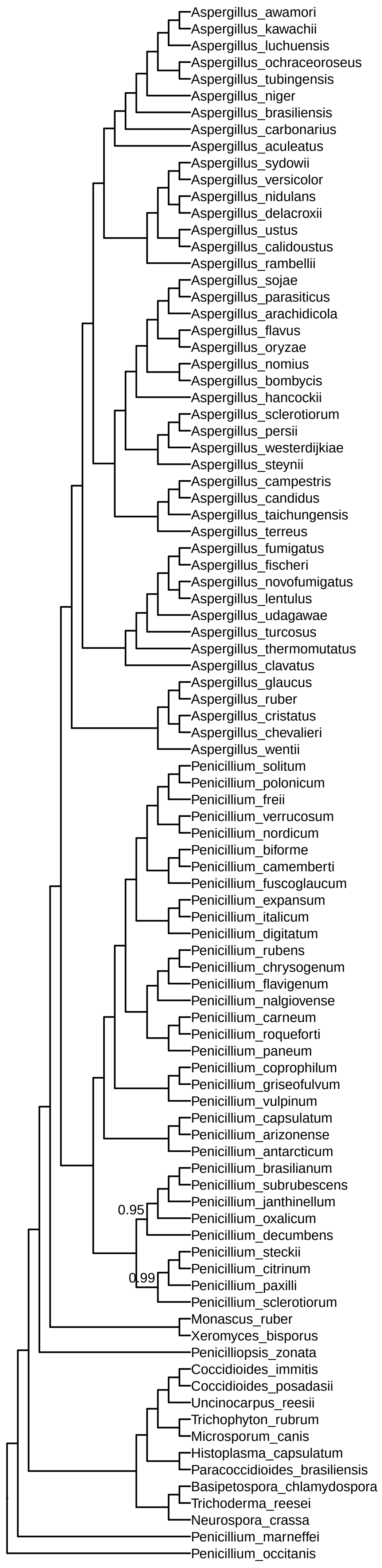

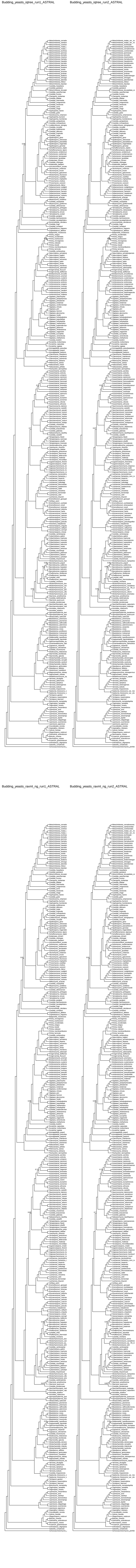
Budding_yeasts_iqtree_run1_ASTRAL
Budding_yeasts_iqtree_run2_ASTRAL
Budding_yeasts_raxml_ng_run1_ASTRAL
Budding_yeasts_raxml_ng_run2_ASTRAL

Hanseniaspora_iqtree_run1_ASTRAL

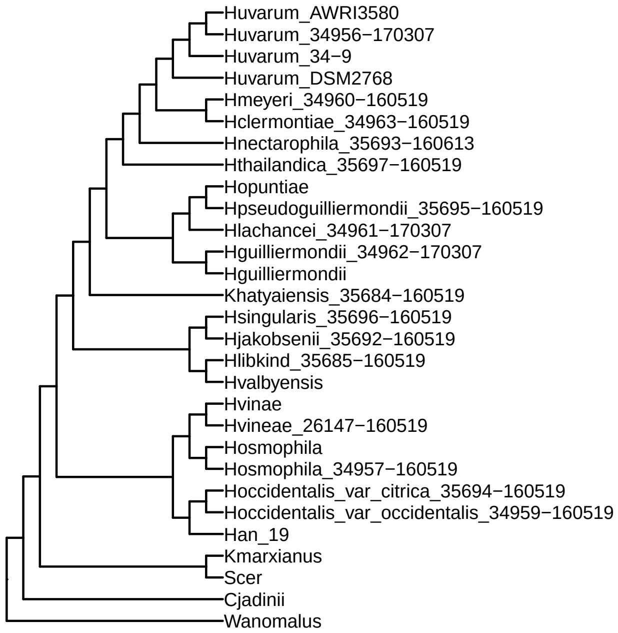

Hanseniaspora_iqtree_run2_ASTRAL

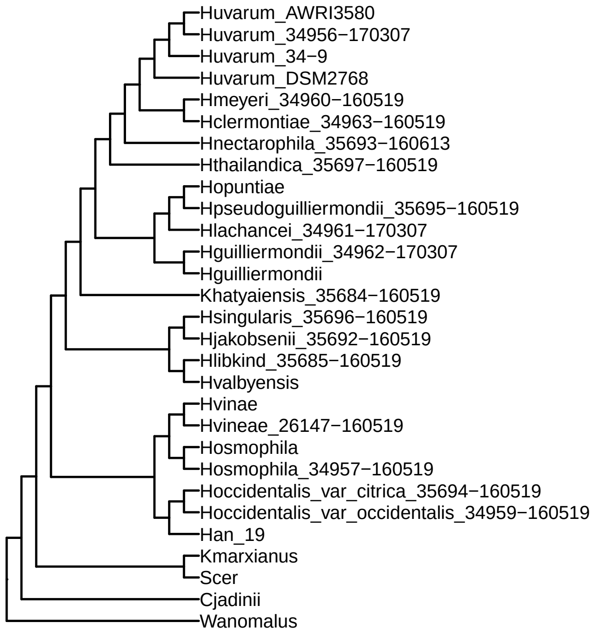

Hanseniaspora_raxml_ng_run1_ASTRAL

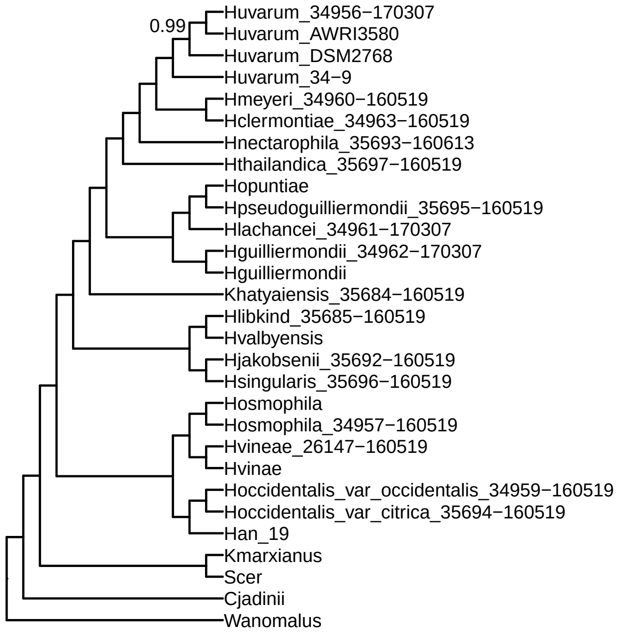

Hanseniaspora_raxml_ng_run2_ASTRAL

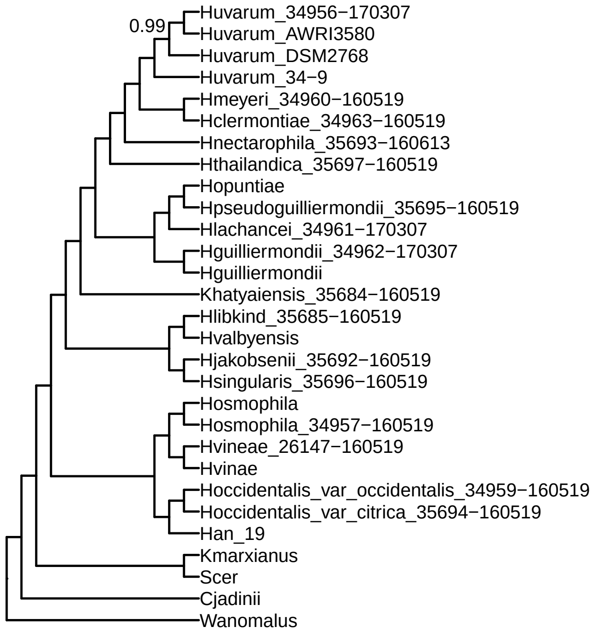

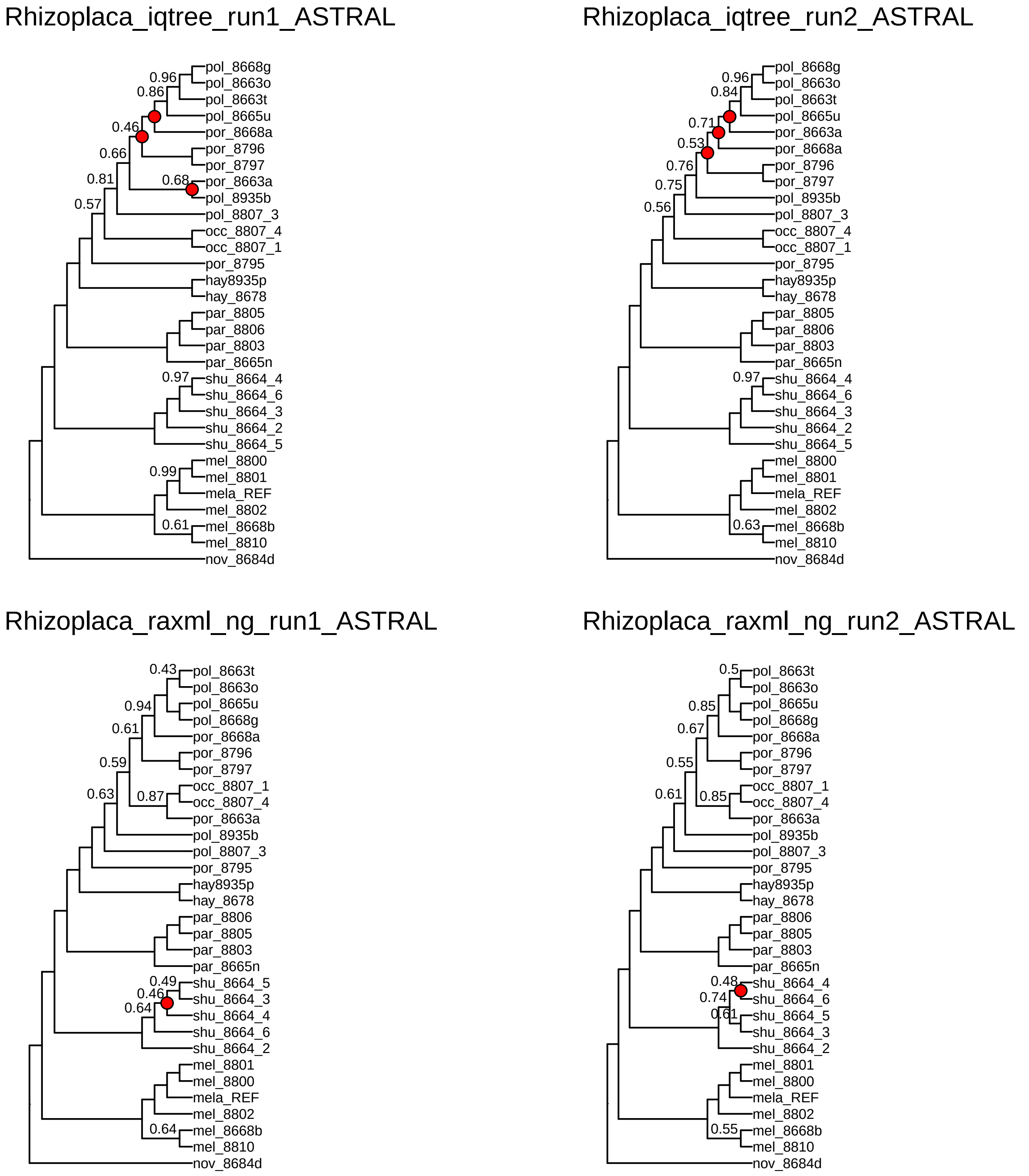

Rhizoplaca_iqtree_run1_ASTRAL
0.96
0.86
0.46
0.66
0.81
0.68
0.57
0.97
0.99
0.61
pol_8668g
pol_8663o
pol_8663t
pol_8665u
por_8668a
por_8796
por_8797
por_8663a
pol_8935b
pol_8807_3
occ_8807_4
occ_8807_1
por_8795
hay8935p
hay_8678
par_8805
par_8806
par_8803
par_8665n
shu_8664_4
shu_8664_6
shu_8664_3
shu_8664_2
shu_8664_5
mel_8800
mel_8801
mela_REF
mel_8802
mel_8668b
mel_8810
nov_8684d
Rhizoplaca_iqtree_run2_ASTRAL
0.96
0.84
0.71
0.53
0.76
0.75
0.56
0.97
0.63
pol_8668g
pol_8663o
pol_8663t
pol_8665u
por_8663a
por_8668a
por_8796
por_8797
pol_8935b
pol_8807_3
occ_8807_4
occ_8807_1
por_8795
hay8935p
hay_8678
par_8805
par_8806
par_8803
par_8665n
shu_8664_4
shu_8664_6
shu_8664_3
shu_8664_2
shu_8664_5
mel_8800
mel_8801
mela_REF
mel_8802
mel_8668b
mel_8810
nov_8684d
Rhizoplaca_raxml_ng_run1_ASTRAL
0.43
0.94
0.61
0.59
0.63
0.87
0.49
0.46
0.64
0.64
pol_8663t
pol_8663o
pol_8665u
pol_8668g
por_8668a
por_8796
por_8797
occ_8807_1
occ_8807_4
por_8663a
pol_8935b
pol_8807_3
por_8795
hay8935p
hay_8678
par_8806
par_8805
par_8803
par_8665n
shu_8664_5
shu_8664_3
shu_8664_4
shu_8664_6
shu_8664_2
mel_8801
mel_8800
mela_REF
mel_8802
mel_8668b
mel_8810
nov_8684d
Rhizoplaca_raxml_ng_run2_ASTRAL
0.5
0.85
0.67
0.55
0.61
0.85
0.48
0.74
0.61
0.55
pol_8663t
pol_8663o
pol_8665u
pol_8668g
por_8668a
por_8796
por_8797
occ_8807_1
occ_8807_4
por_8663a
pol_8935b
pol_8807_3
por_8795
hay8935p
hay_8678
par_8806
par_8805
par_8803
par_8665n
shu_8664_4
shu_8664_6
shu_8664_5
shu_8664_3
shu_8664_2
mel_8801
mel_8800
mela_REF
mel_8802
mel_8668b
mel_8810
nov_8684d

Supplementary Figure 13. Concatenation-based ML trees for 15 phylogenomic datasets. For each of 15 datasets, concatenation-based ML trees were inferred twice from the supermatrix using IQ-TREE and RAxML-NG with identical settings. We visualized phylogenetic trees using the R package ggtree. 15 / 15 phylogenomic datasets produced topologically identical concatenation-based ML species phylogenies when we inferred them twice independently from the same supermatrix using identical parameter settings in either IQ-TREE or RAxML-NG. All phylogenomic datasets analyzed by RAxML-NG yielded exactly the same concatenation-based ML species phylogeny, where the topology, branch length, and branch support are identical across Run1 and Run2. In contrast, only 4 / 15 (26%) phylogenomic datasets analyzed by IQ-TREE yielded exactly the same concatenation-based ML species phylogeny where the topology, branch length, and branch support are identical across Run1 and Run2.

Order	Dataset	Program	Topological difference (%) [a]	Branch distance [b]	Bipartitions with different support values (%) [c]
1	Animal: Bees	IQ-TREE	**0**	1.46E-07	2.7
1	Animal: Bees	RAxML-NG	**0**	**0**	**0**
2	Animal: Birds	IQ-TREE	**0**	4.09E-09	1.5
2	Animal: Birds	RAxML-NG	**0**	**0**	**0**
3	Animal: Butterflies	IQ-TREE	**0**	0.000283233	16.7
3	Animal: Butterflies	RAxML-NG	**0**	**0**	**0**
4	Animal: Lizards	IQ-TREE	**0**	1.49E-07	**0**
4	Animal: Lizards	RAxML-NG	**0**	**0**	**0**
5	Animal: Marine fishes	IQ-TREE	**0**	1.03E-08	0.9
5	Animal: Marine fishes	RAxML-NG	**0**	**0**	**0**
6	Animal: Rodents	IQ-TREE	**0**	2.83E-10	**0**
6	Animal: Rodents	RAxML-NG	**0**	**0**	**0**
7	Plant: Cardueae	IQ-TREE	**0**	2.45E-10	**0**
7	Plant: Cardueae	RAxML-NG	**0**	**0**	**0**
8	Plant: Caryophyllales	IQ-TREE	**0**	**0**	**0**
8	Plant: Caryophyllales	RAxML-NG	**0**	**0**	**0**
9	Plant: Green plants	IQ-TREE	**0**	**0**	NA
9	Plant: Green plants	RAxML-NG	**0**	**0**	**0**
10	Plant: Jaltomata	IQ-TREE	**0**	8.00E-10	**0**
10	Plant: Jaltomata	RAxML-NG	**0**	**0**	**0**
11	Plant: Protea	IQ-TREE	**0**	3.46E-10	**0**
11	Plant: Protea	RAxML-NG	**0**	**0**	**0**
12	Fungi: Aspergillaceae	IQ-TREE	**0**	1.48E-07	**0**
12	Fungi: Aspergillaceae	RAxML-NG	**0**	**0**	**0**
13	Fungi: Budding yeasts	IQ-TREE	**0**	**0**	**0**
13	Fungi: Budding yeasts	RAxML-NG	**0**	**0**	NA
14	Fungi: Hanseniaspora	IQ-TREE	**0**	**0**	**0**
14	Fungi: Hanseniaspora	RAxML-NG	**0**	**0**	**0**
15	Fungi: Rhizoplaca	IQ-TREE	**0**	3.32E-10	**0**
15	Fungi: Rhizoplaca	RAxML-NG	**0**	**0**	**0**

[a] Percentage of bipartitions (or internal branches) that differ between two inferred species trees was quantified the normalized Robinson–Foulds tree distance.
[b] Branch distance between two inferred species trees was computed by the branch score distance of Kuhner and Felsenstein with the R packages ape and phangorn.
[c] The percentage of bipartitions (or internal branches) that received different bootstrap support values between two inferred species trees that were topologically identical (i.e., Topological difference is 0).

Bee_iqtree_run1_Concatenation_ML

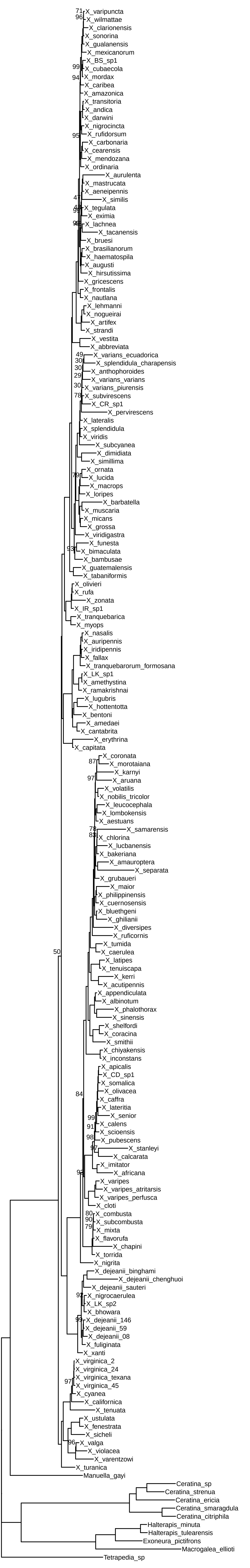

Bee_iqtree_run2_Concatenation_ML

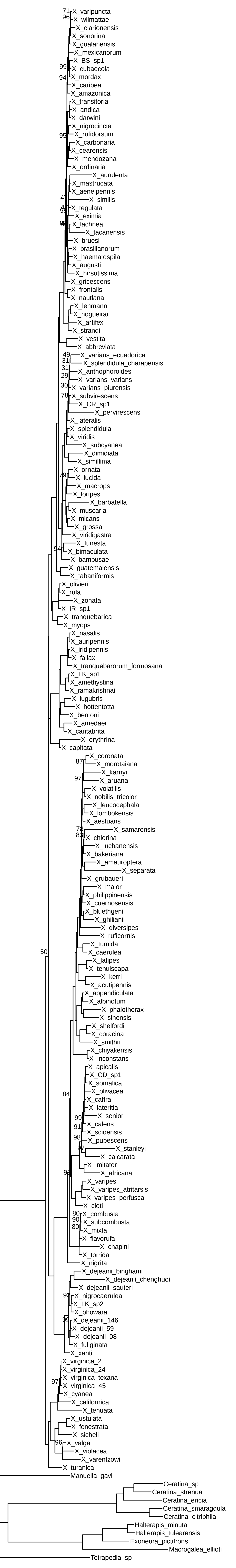

Bee_raxml_ng_run1_Concatenation_ML

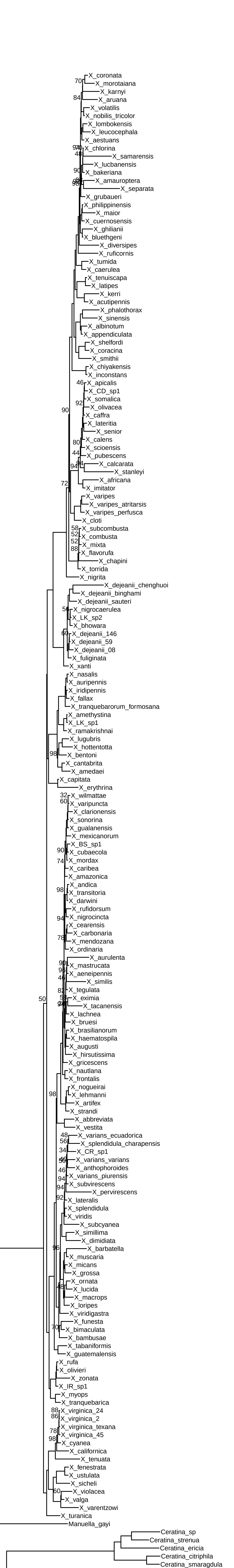

Bee_raxml_ng_run2_Concatenation_ML

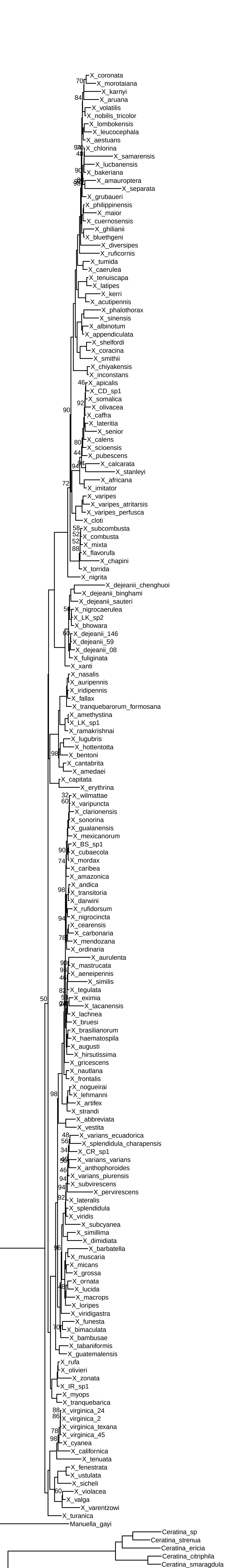

Birds_iqtree_run1_Concatenation_ML

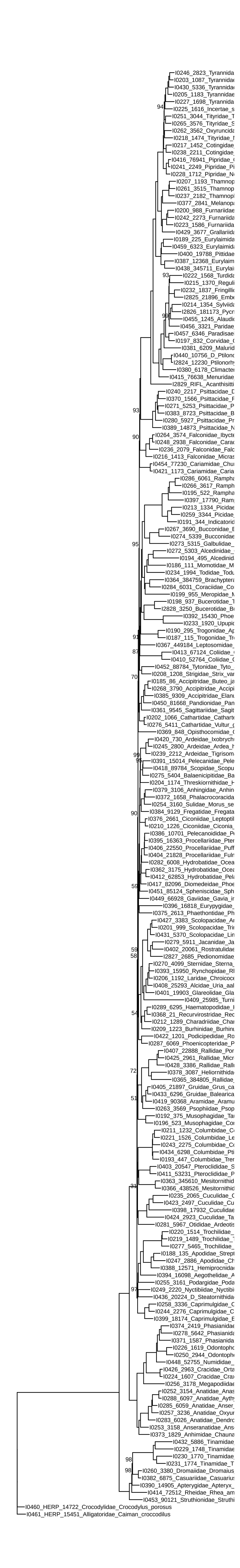

Birds_iqtree_run2_Concatenation_ML

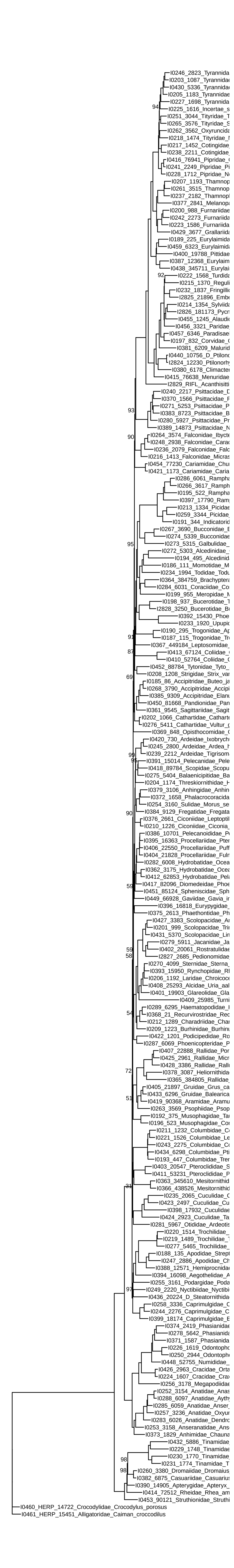

Birds_raxml_ng_run1_Concatenation_ML

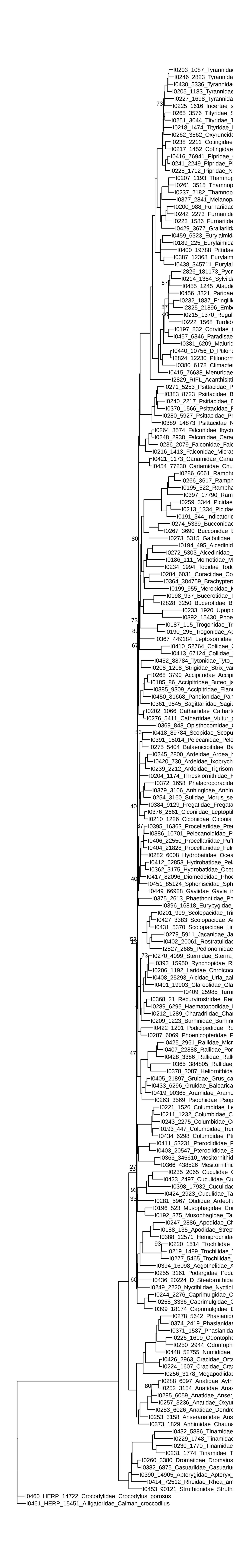

Birds_raxml_ng_run2_Concatenation_ML

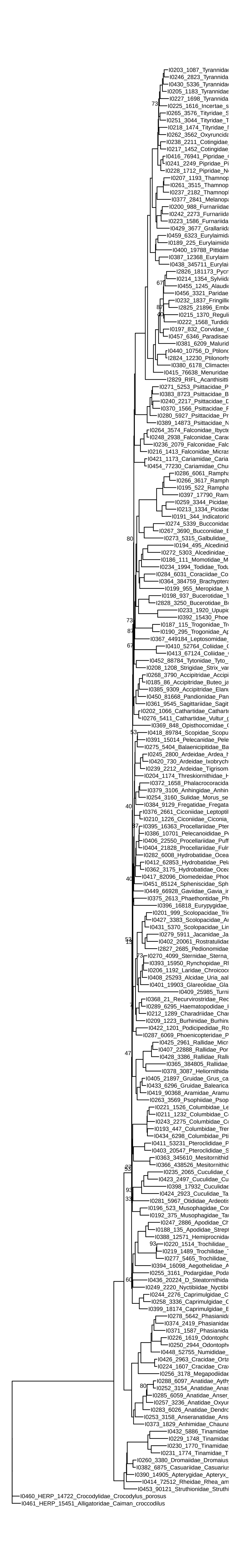

Butterfly_iqtree_run1_Concatenation_ML

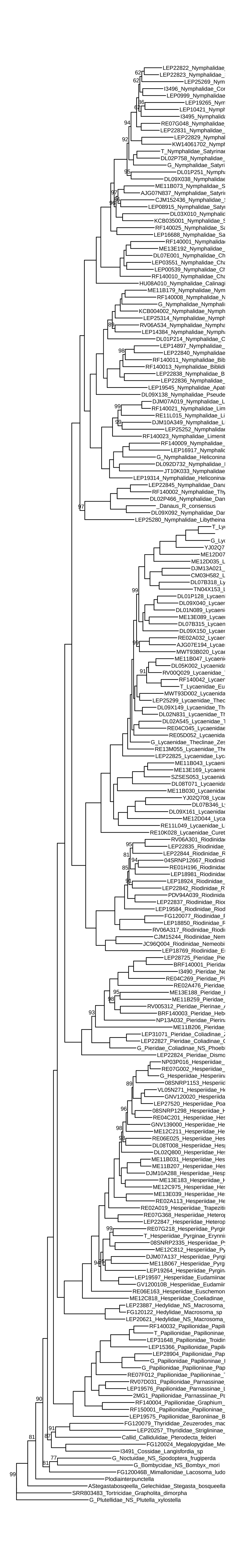

Butterfly_iqtree_run2_Concatenation_ML

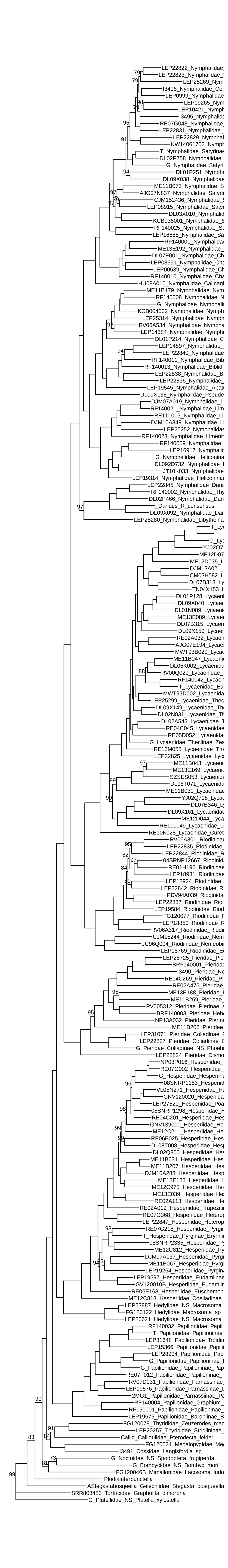

Butterfly_raxml_ng_run1_Concatenation_ML

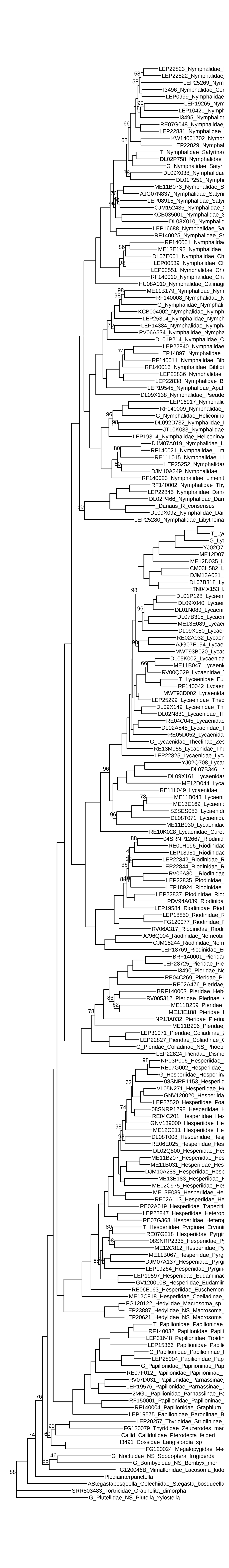

Butterfly_raxml_ng_run2_Concatenation_ML

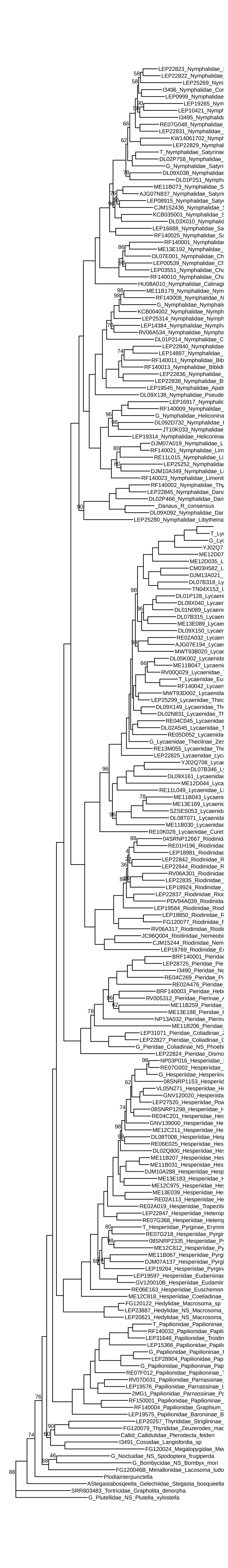

Lizard_iqtree_run1_Concatenation_ML

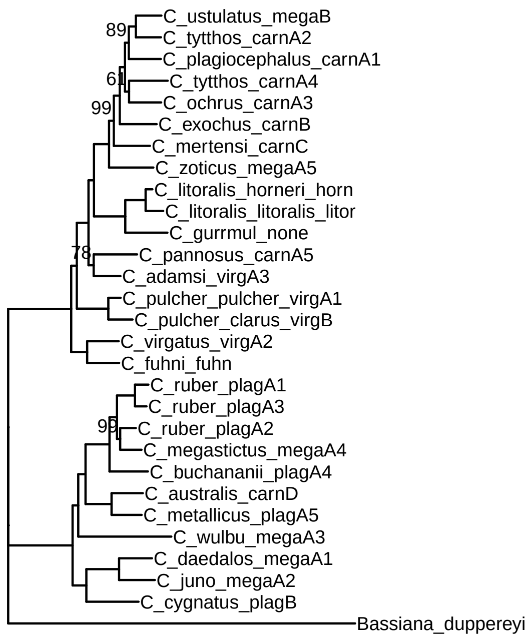

Lizard_iqtree_run2_Concatenation_ML

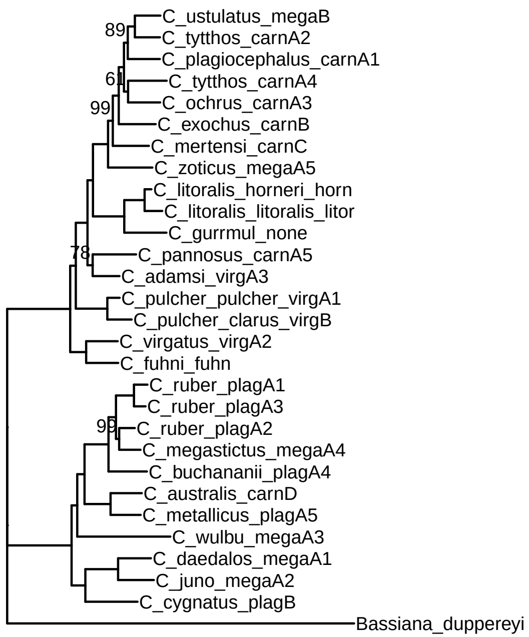

Lizard_raxml_ng_run1_Concatenation_ML

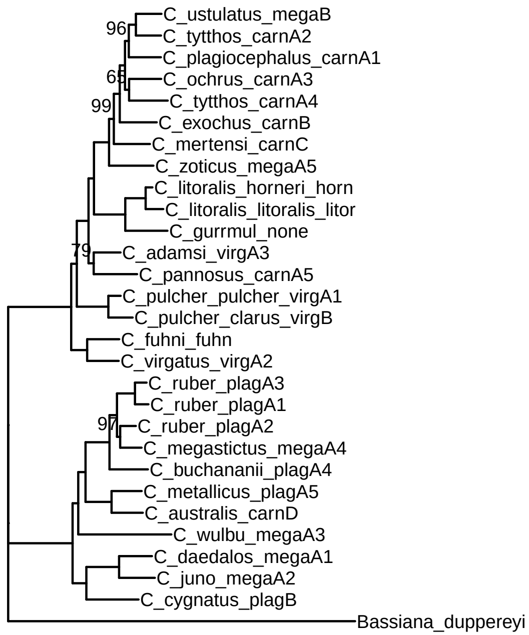

Lizard_raxml_ng_run2_Concatenation_ML

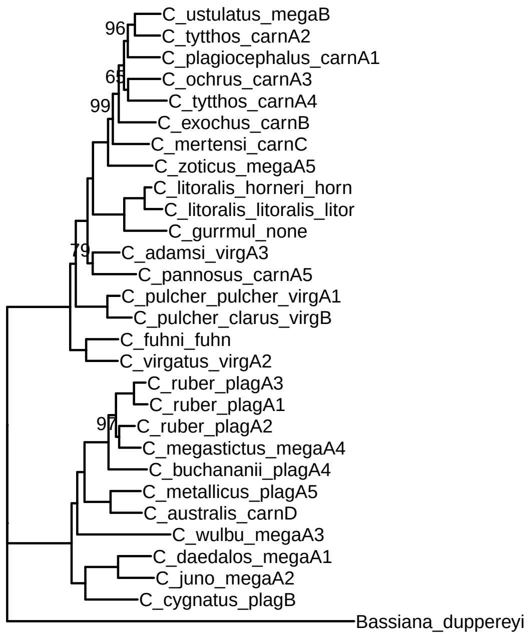

Marine_fish_iqtree_run1_Concatenation_ML

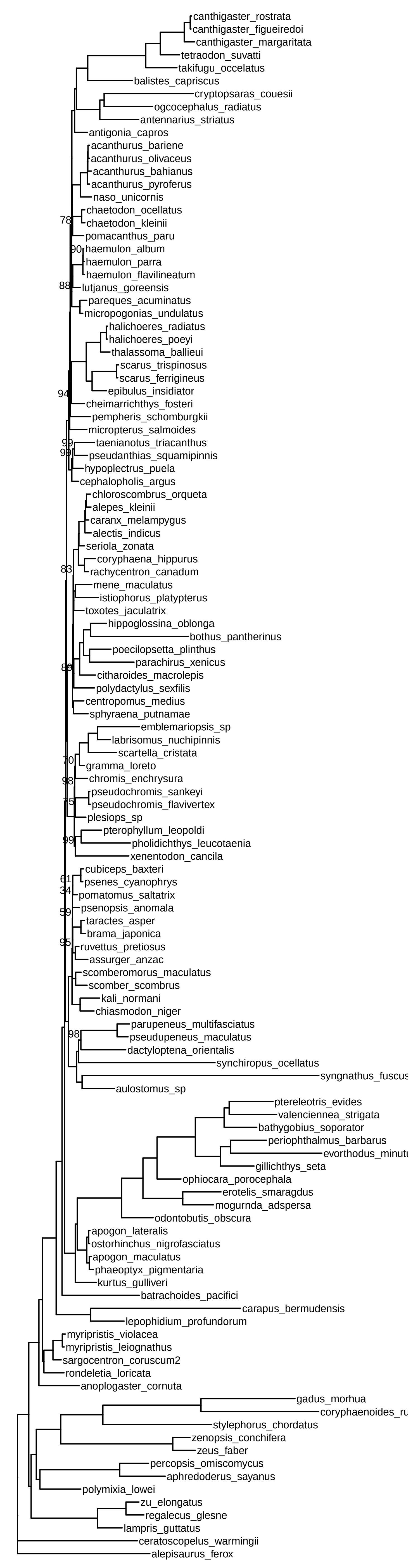

Marine_fish_iqtree_run2_Concatenation_ML

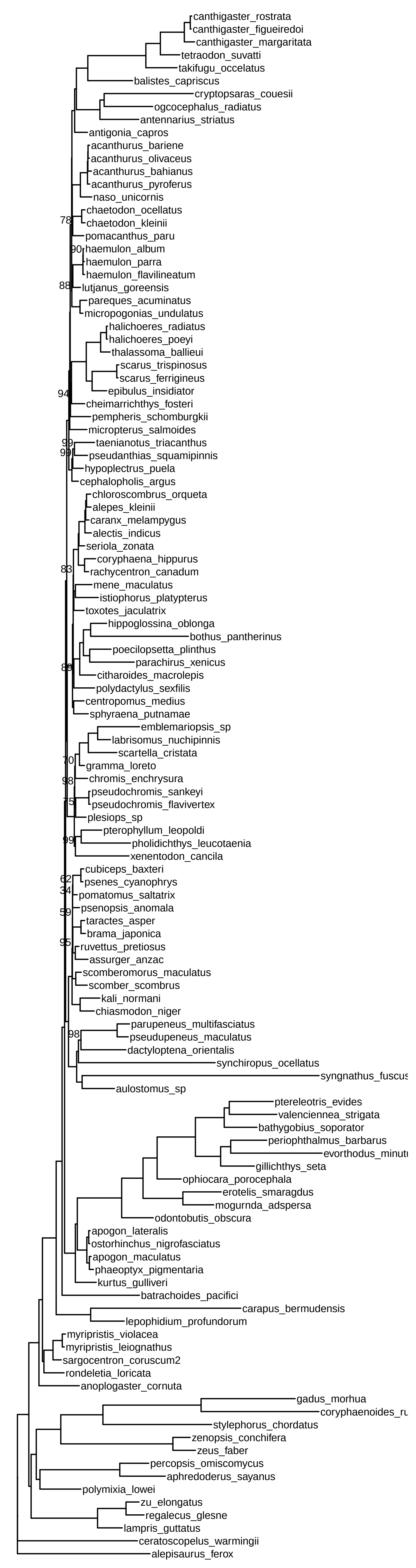

Marine_fish_raxml_ng_run1_Concatenation_ML

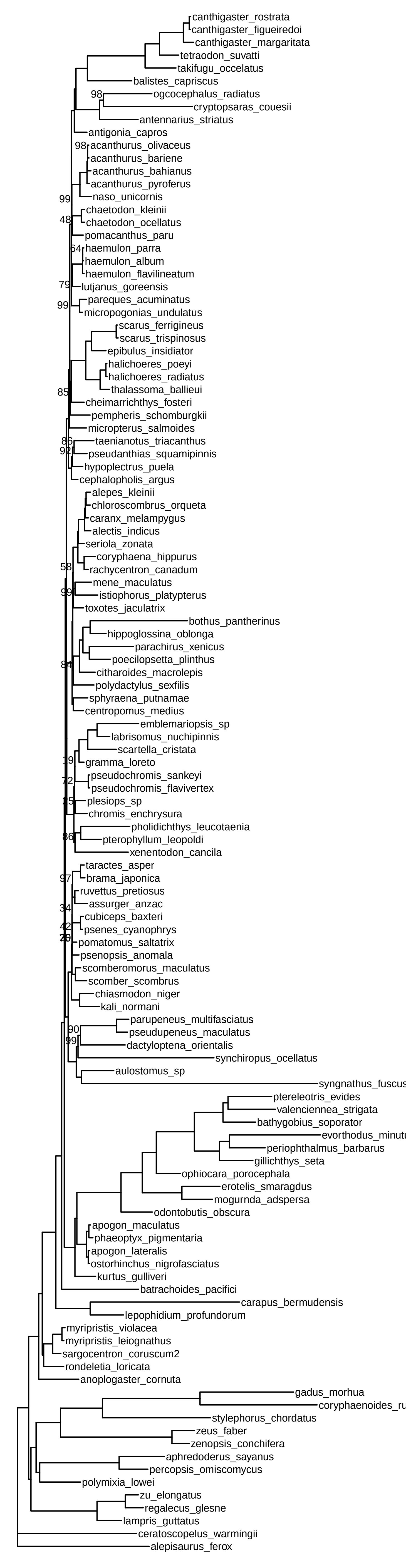

Marine_fish_raxml_ng_run2_Concatenation_ML

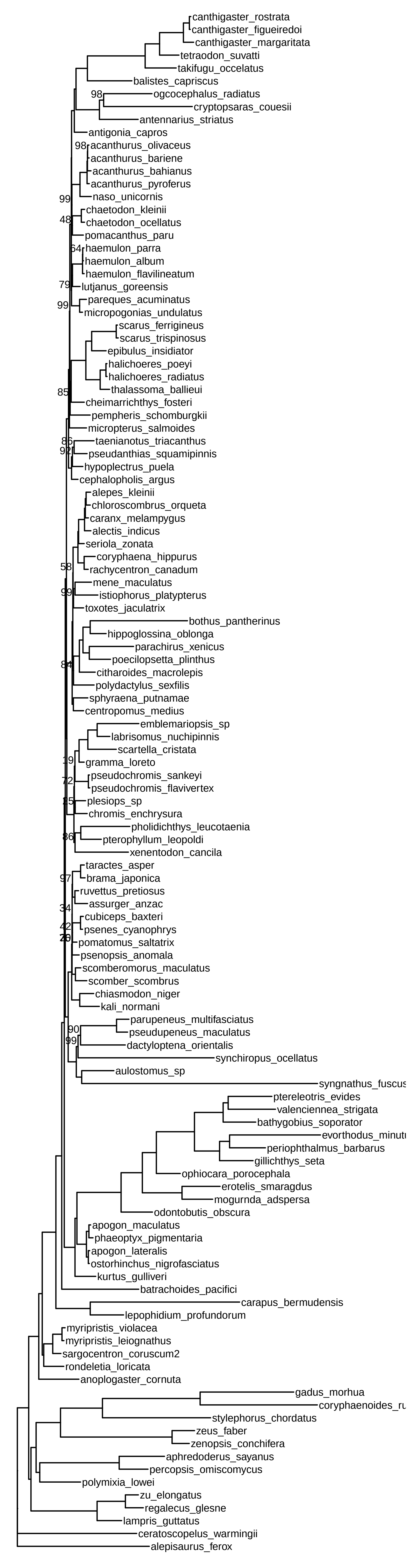

Rodents_iqtree_run1_Concatenation_ML

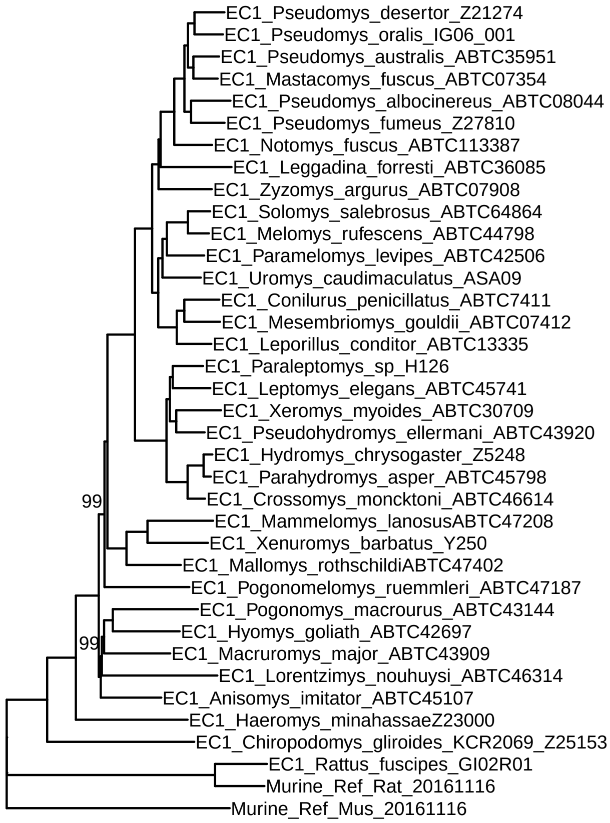

Rodents_iqtree_run2_Concatenation_ML

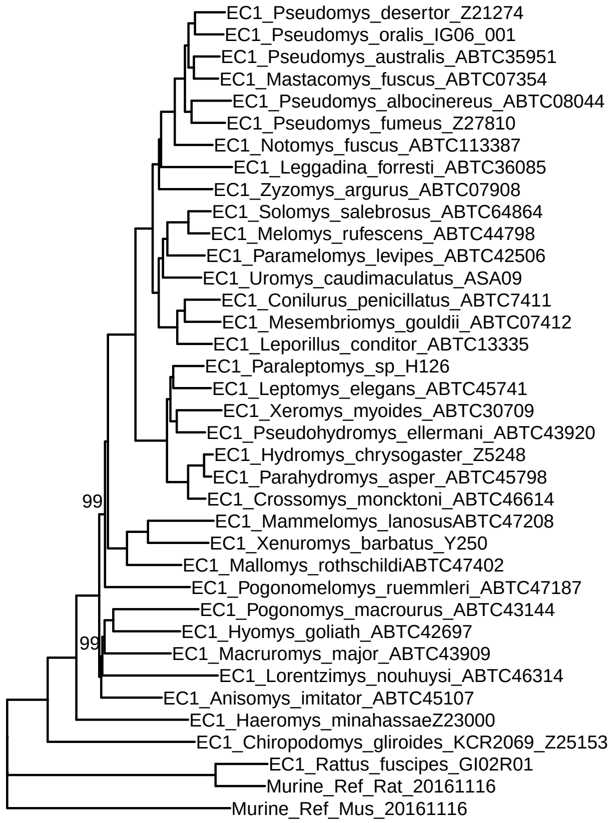

Rodents_raxml_ng_run1_Concatenation_ML

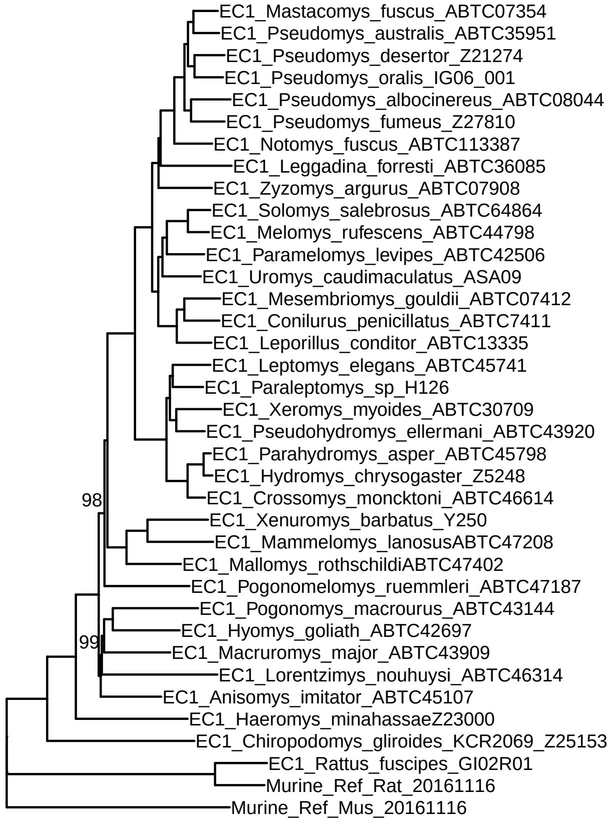

Rodents_raxml_ng_run2_Concatenation_ML

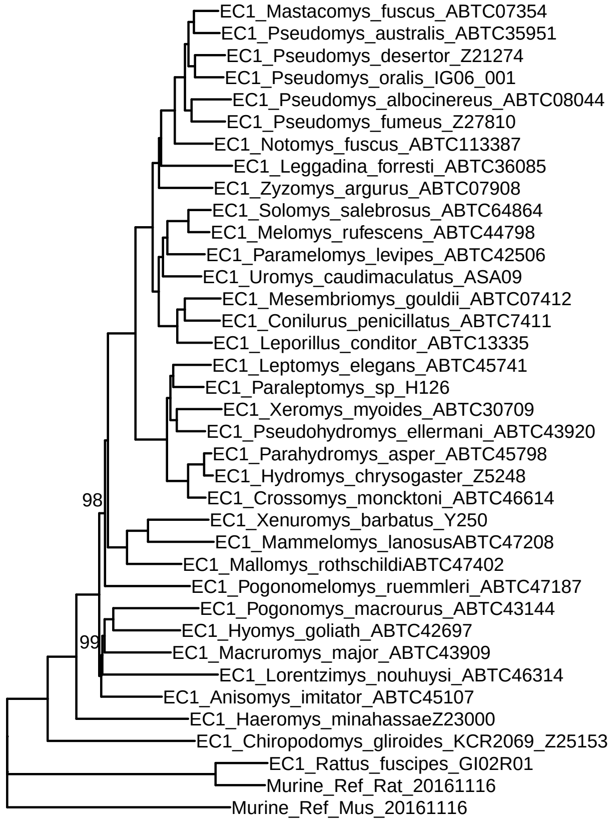

Cardueae_iqtree_run1_Concatenation_ML

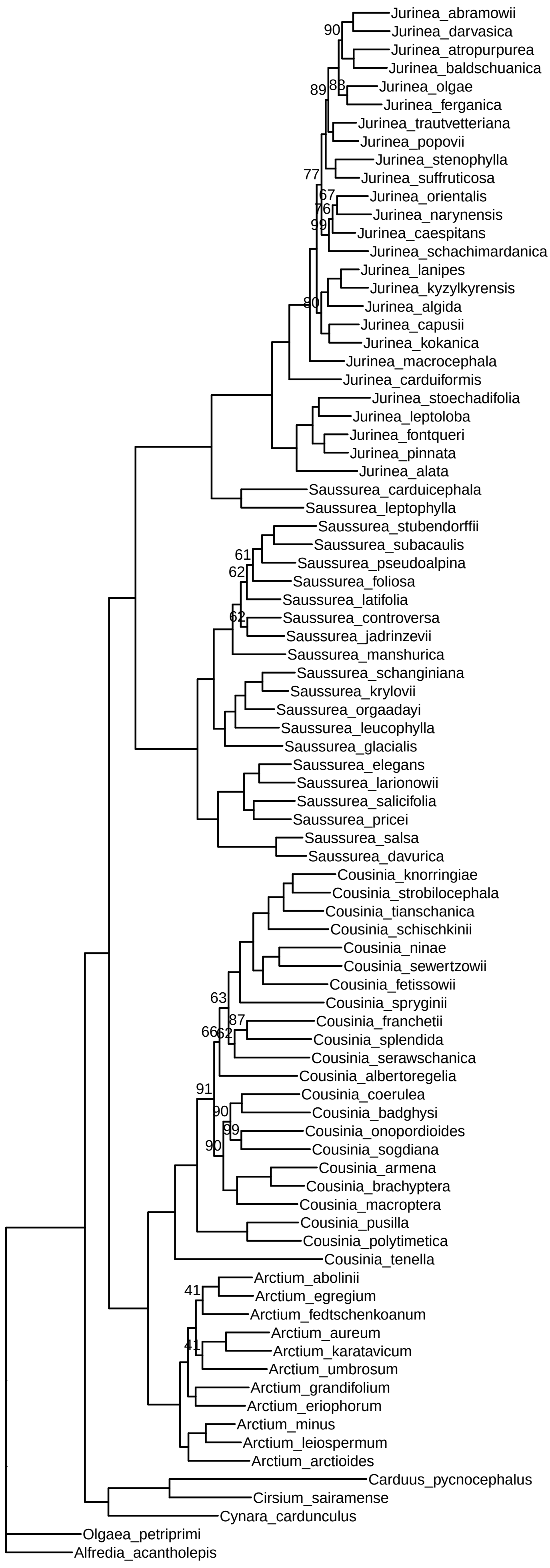

Cardueae_iqtree_run2_Concatenation_ML

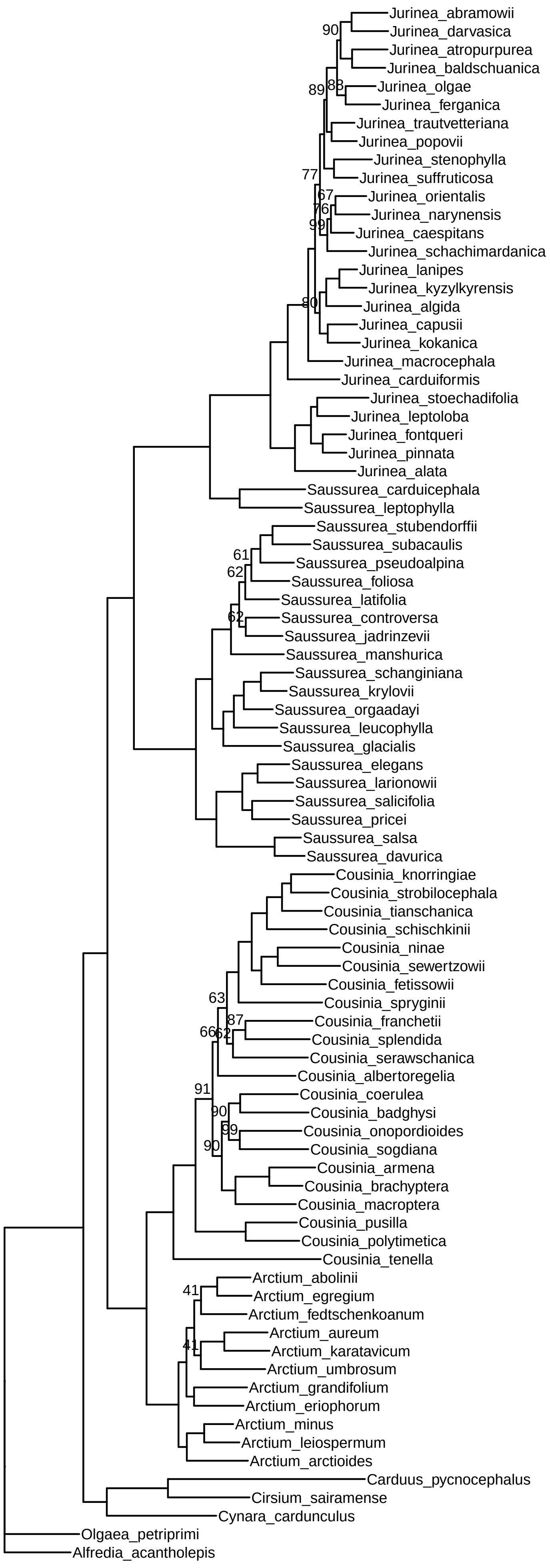

Cardueae_raxml_ng_run1_Concatenation_ML

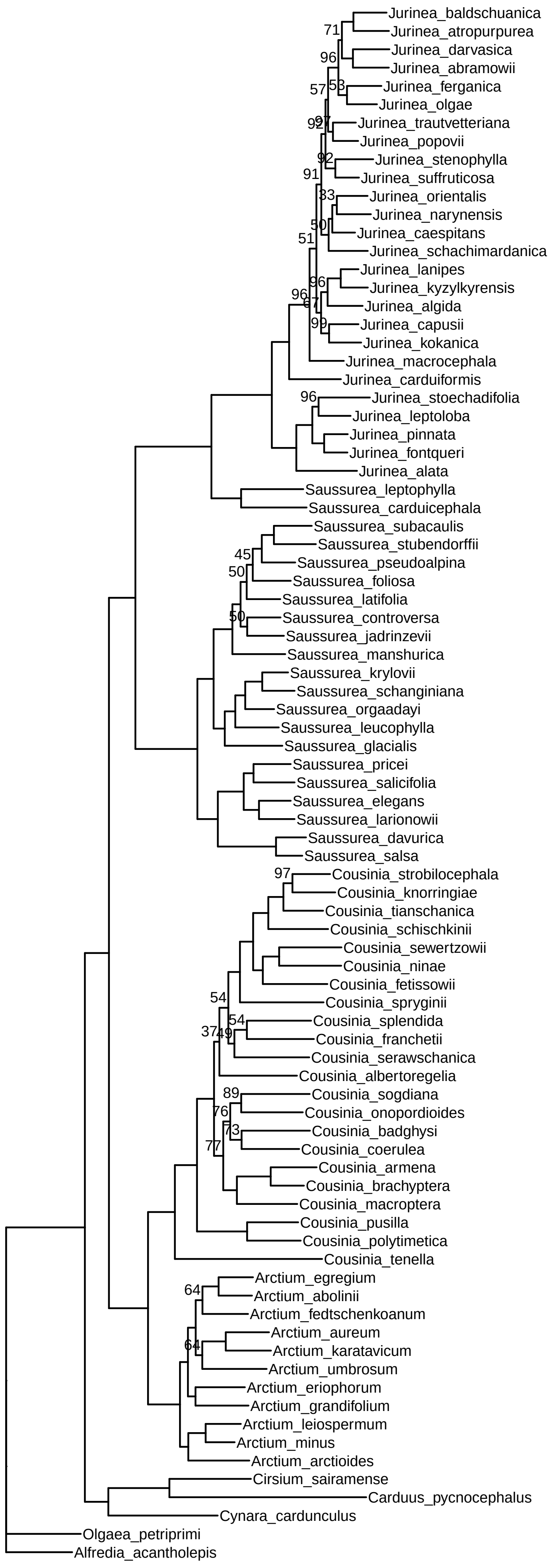

Cardueae_raxml_ng_run2_Concatenation_ML

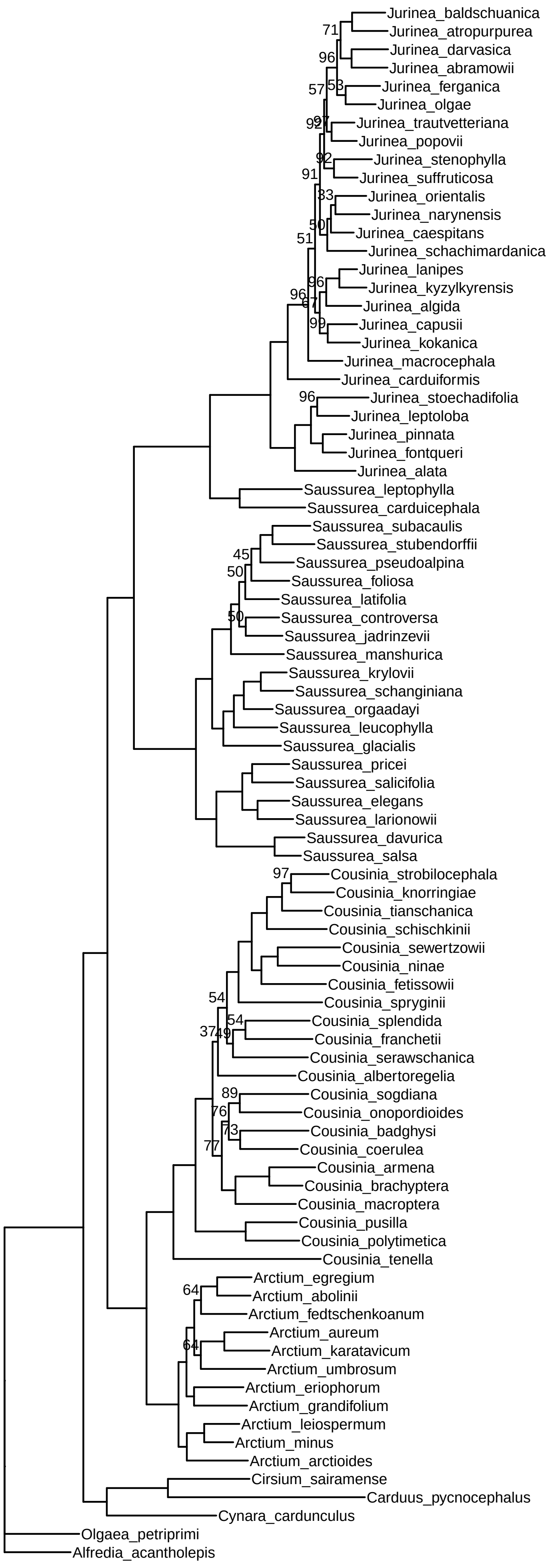

Caryophyllales_iqtree_run1_Concatenation_ML

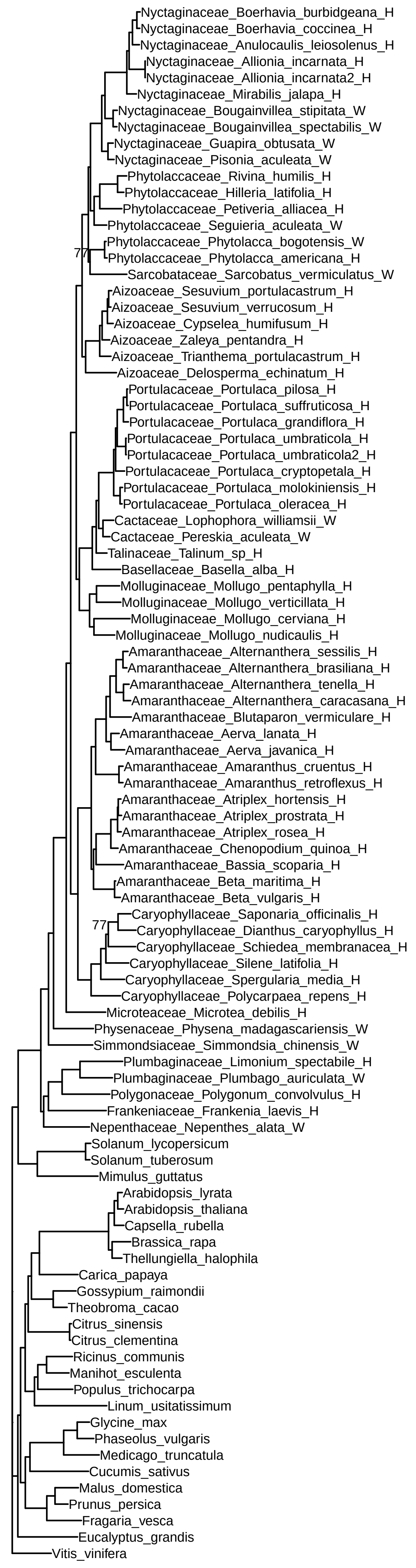

Caryophyllales_iqtree_run2_Concatenation_ML

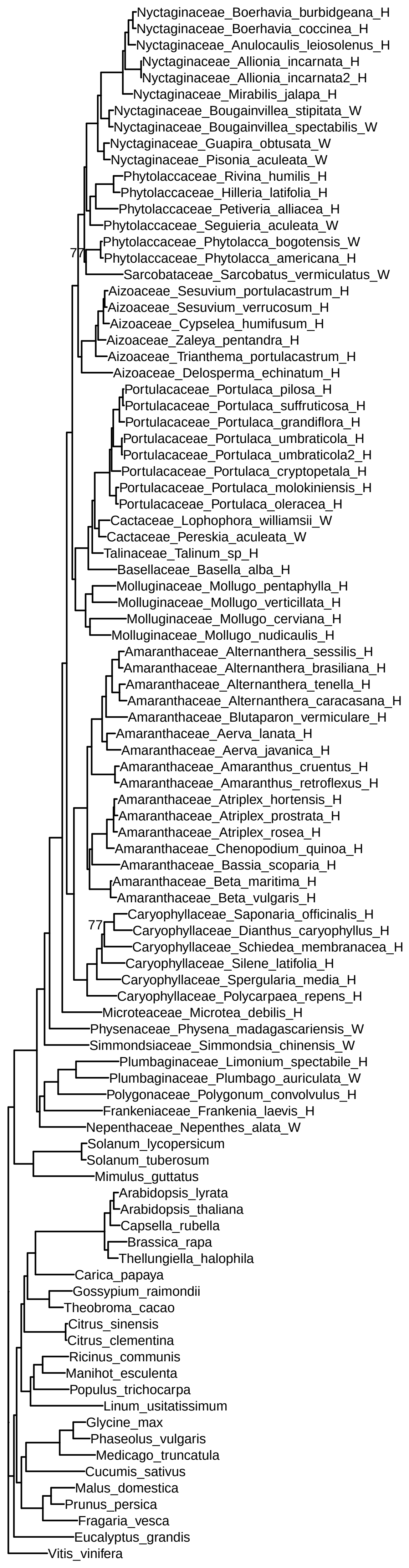

Caryophyllales_raxml_ng_run1_Concatenation_I

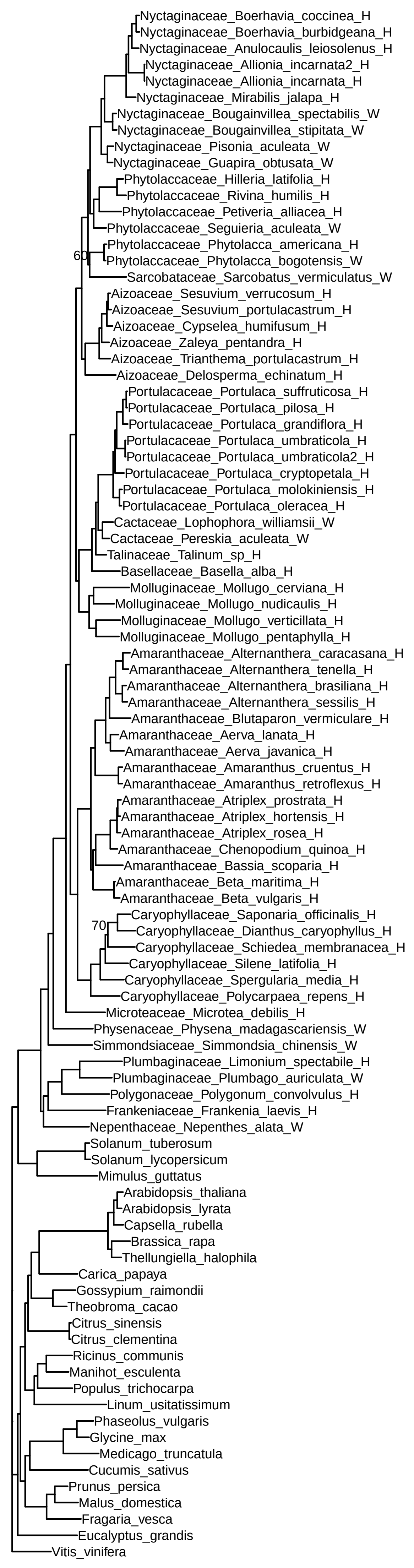

Caryophyllales_raxml_ng_run2_Concatenation_I

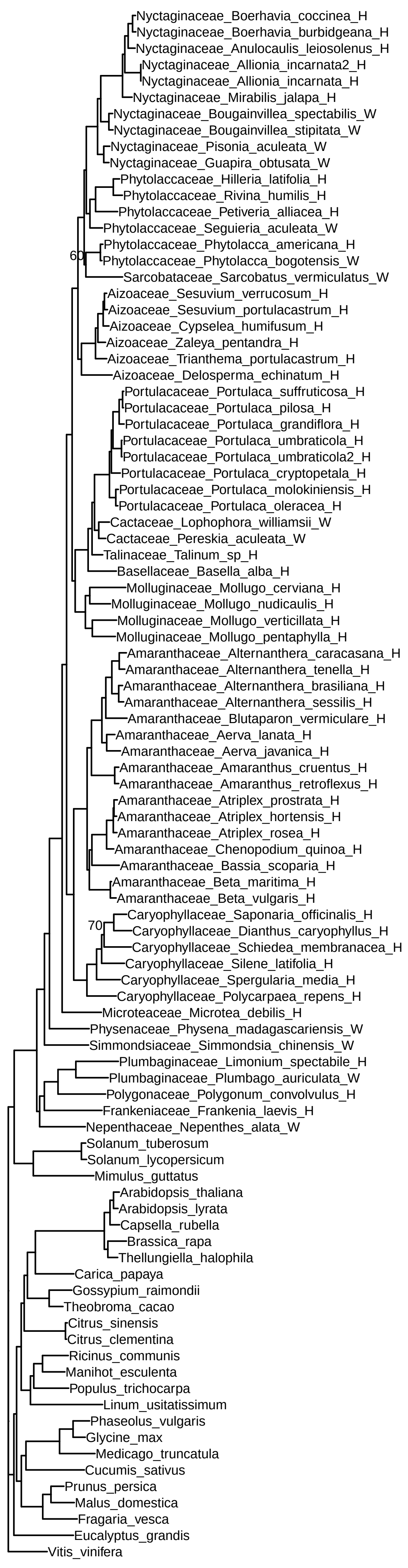

Green_plants_iqtree_run1_Concatenation_ML

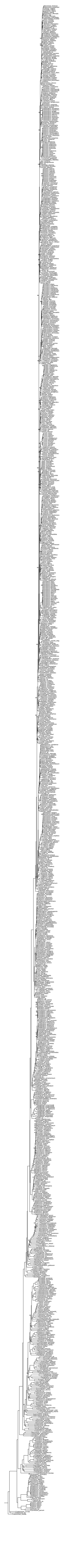

Green_plants_iqtree_run2_Concatenation_ML

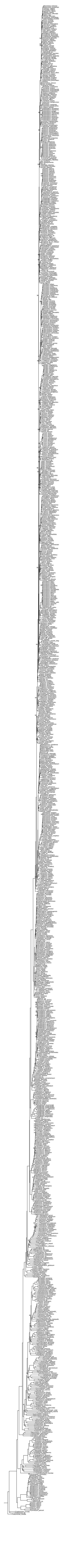

Green_plants_raxml_ng_run1_Concatenation_M Green_plants_raxml_ng_run2_Concatenation_M

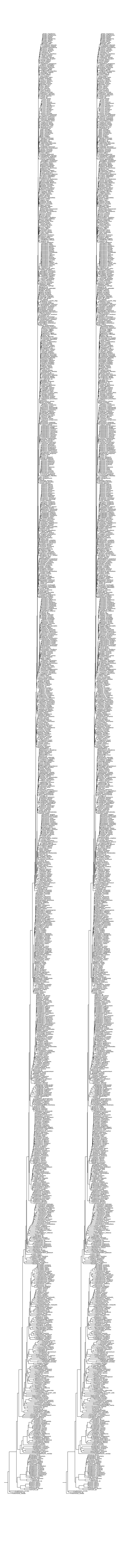

Jaltomata_iqtree_run1_Concatenation_ML

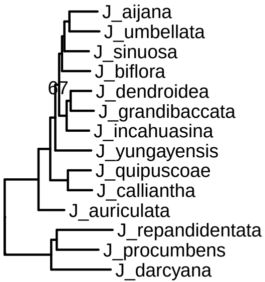

Jaltomata_iqtree_run2_Concatenation_ML

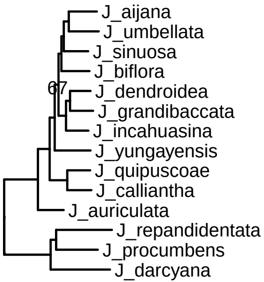

Jaltomata_raxml_ng_run1_Concatenation_ML

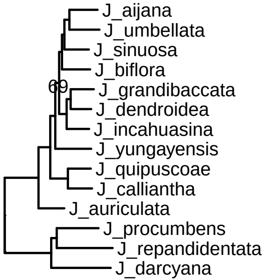

Jaltomata_raxml_ng_run2_Concatenation_ML

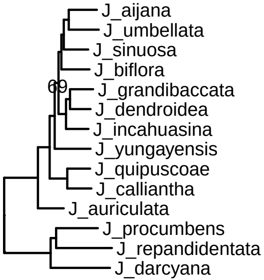

Protea_iqtree_run1_Concatenation_ML

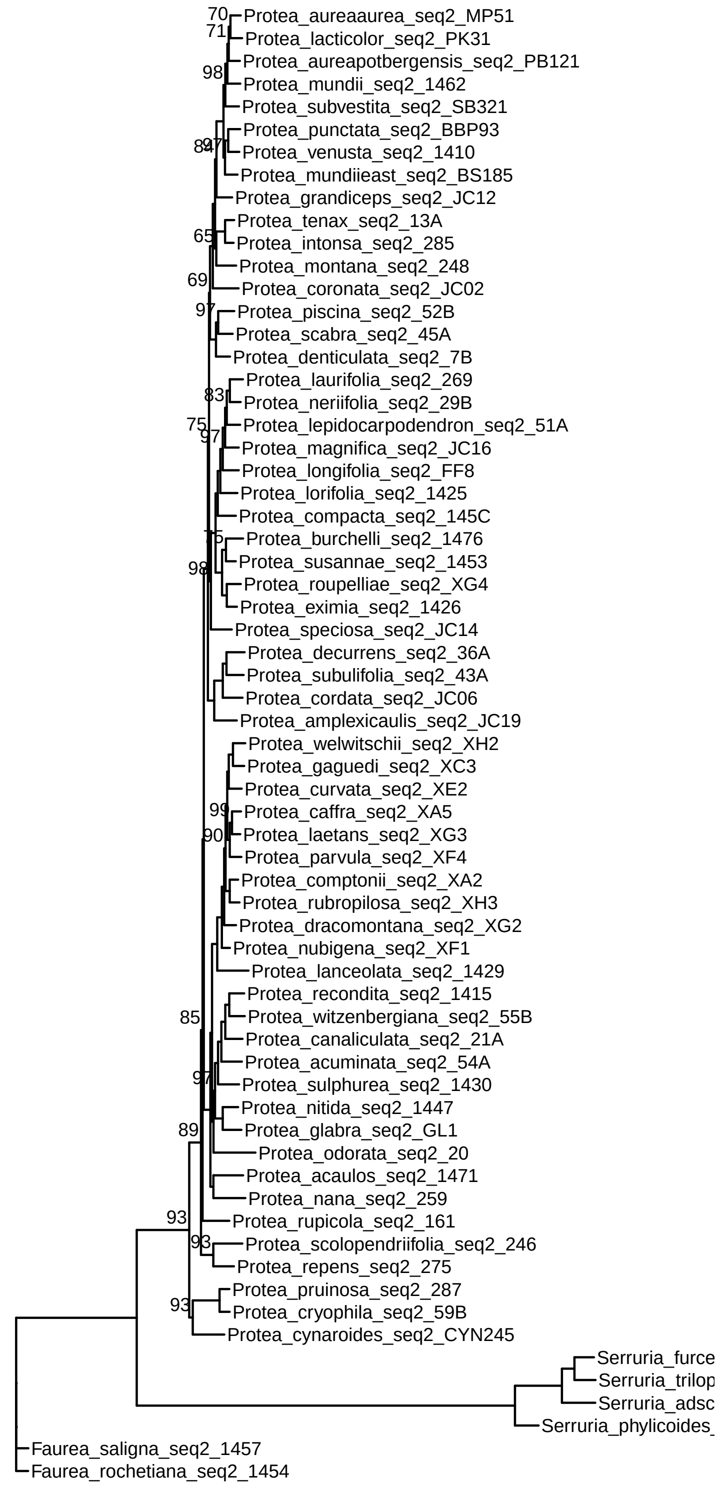

Protea_iqtree_run2_Concatenation_ML

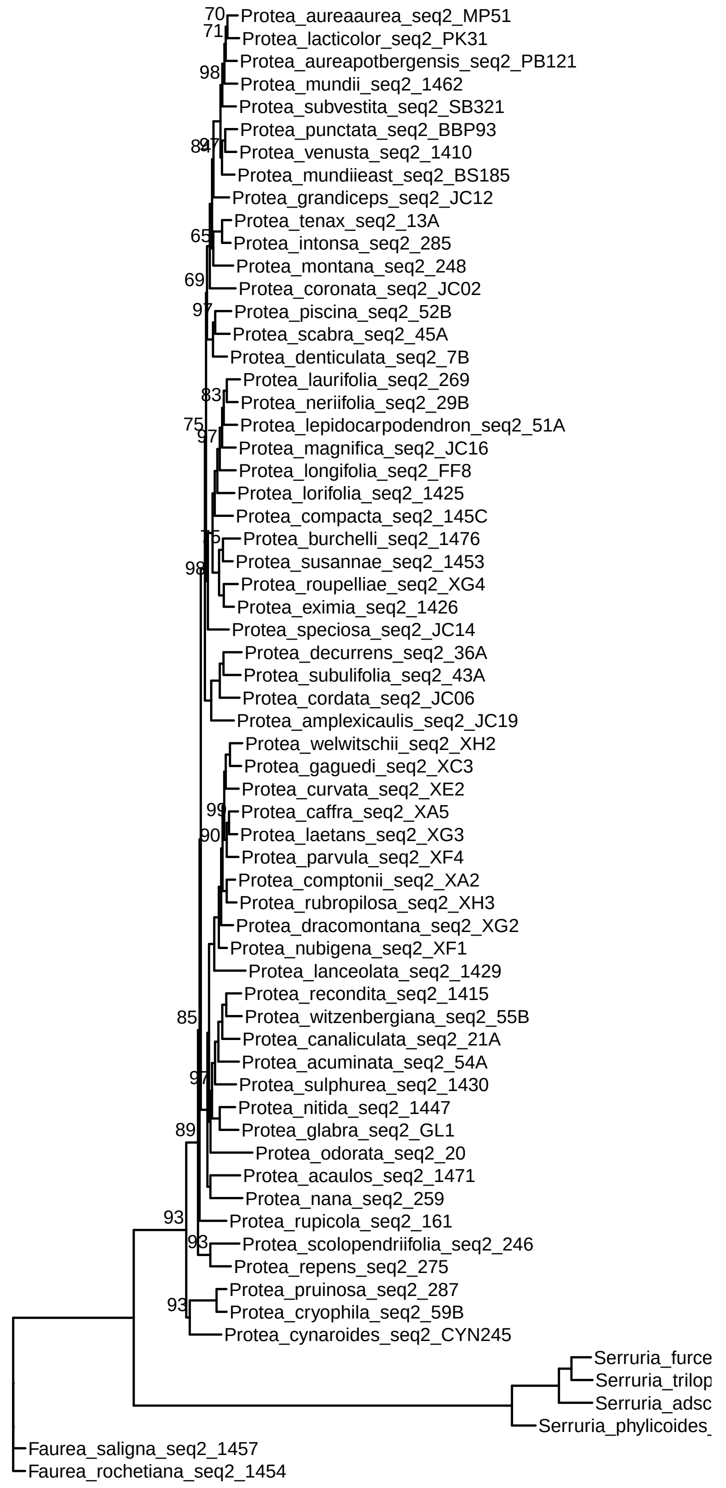

Protea_raxml_ng_run1_Concatenation_ML

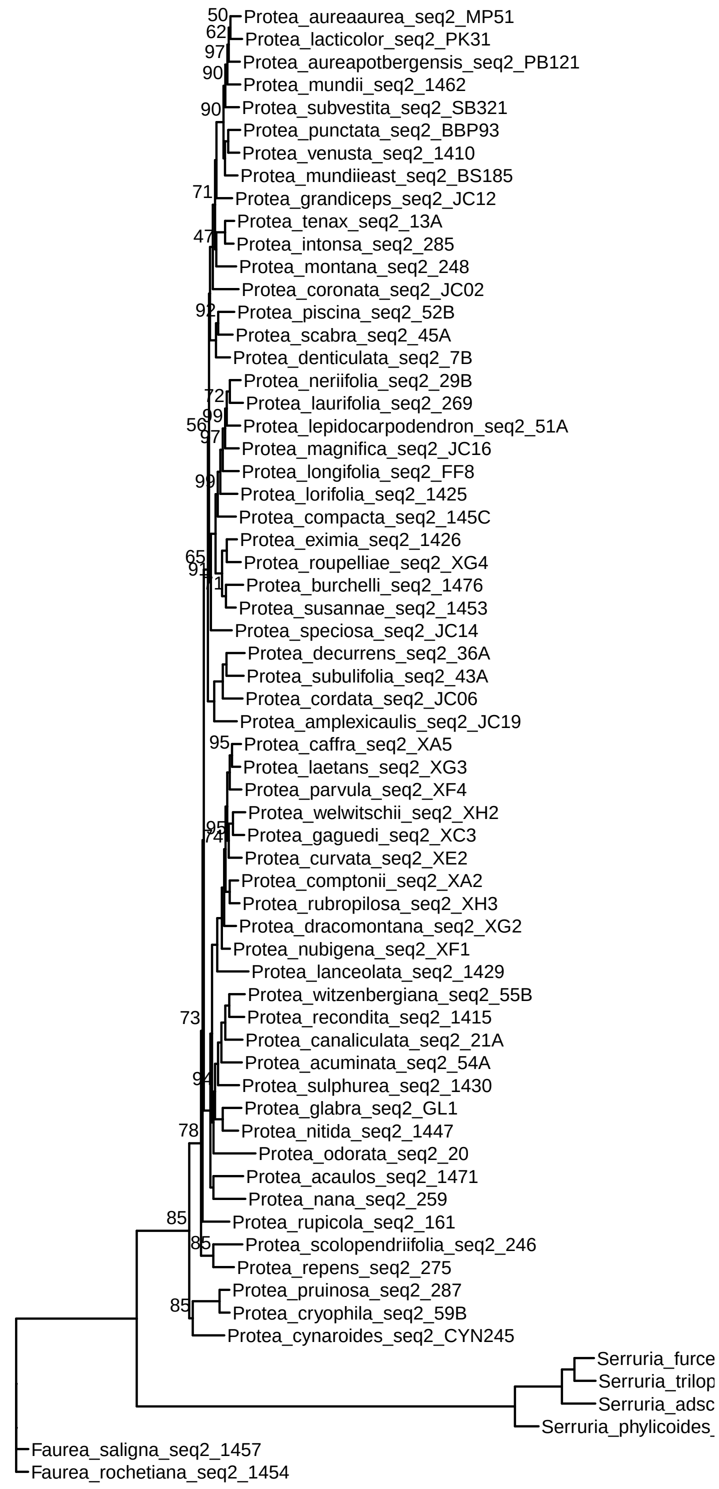

Protea_raxml_ng_run2_Concatenation_ML

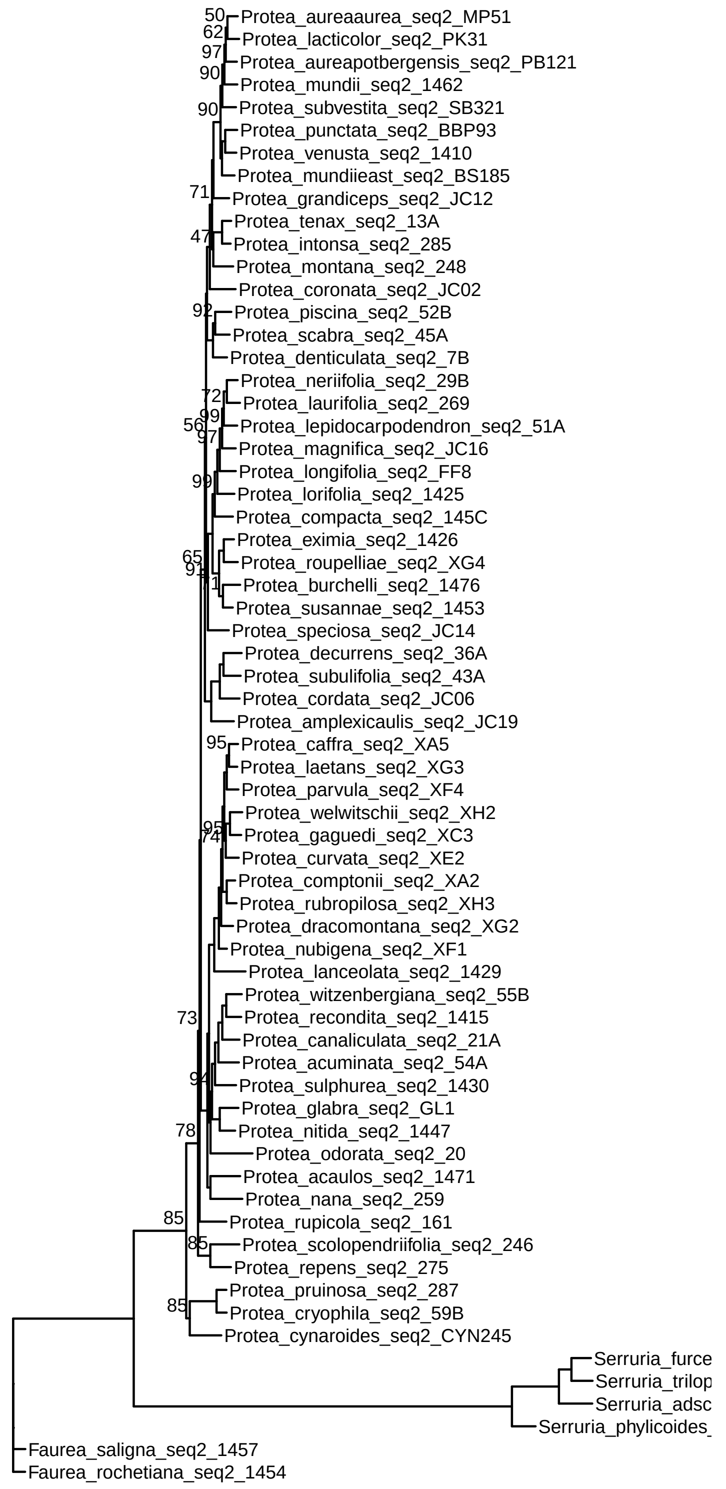

Aspergillaceae_iqtree_run1_Concatenation_ML

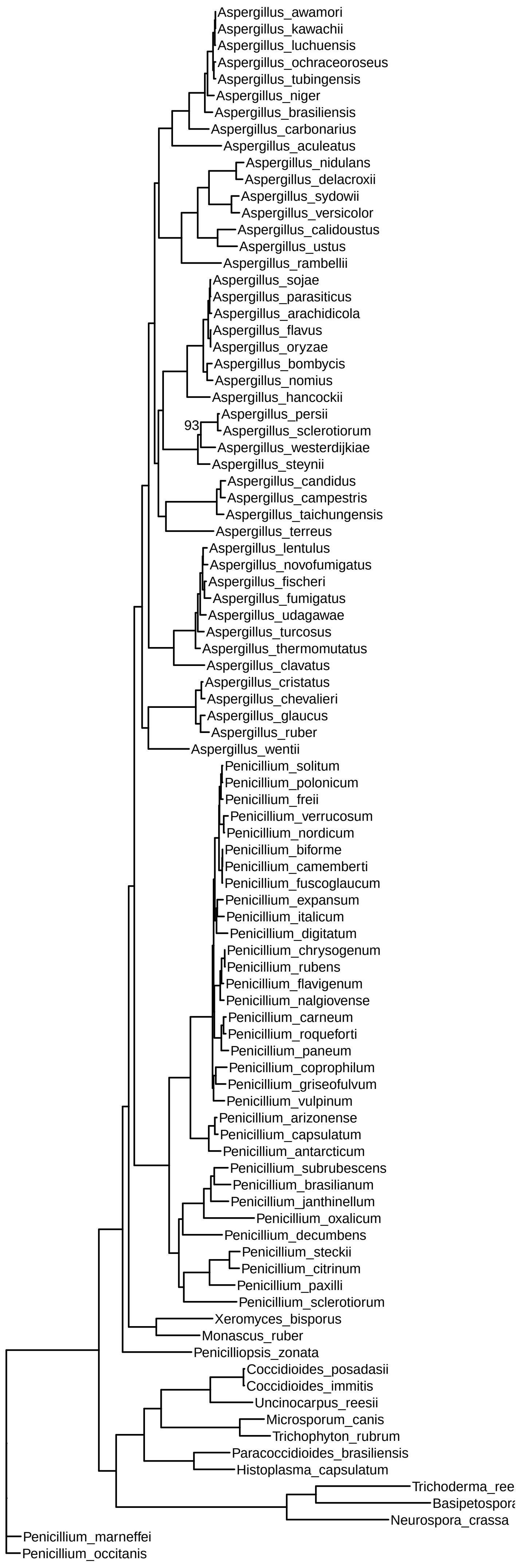

Aspergillaceae_iqtree_run2_Concatenation_ML

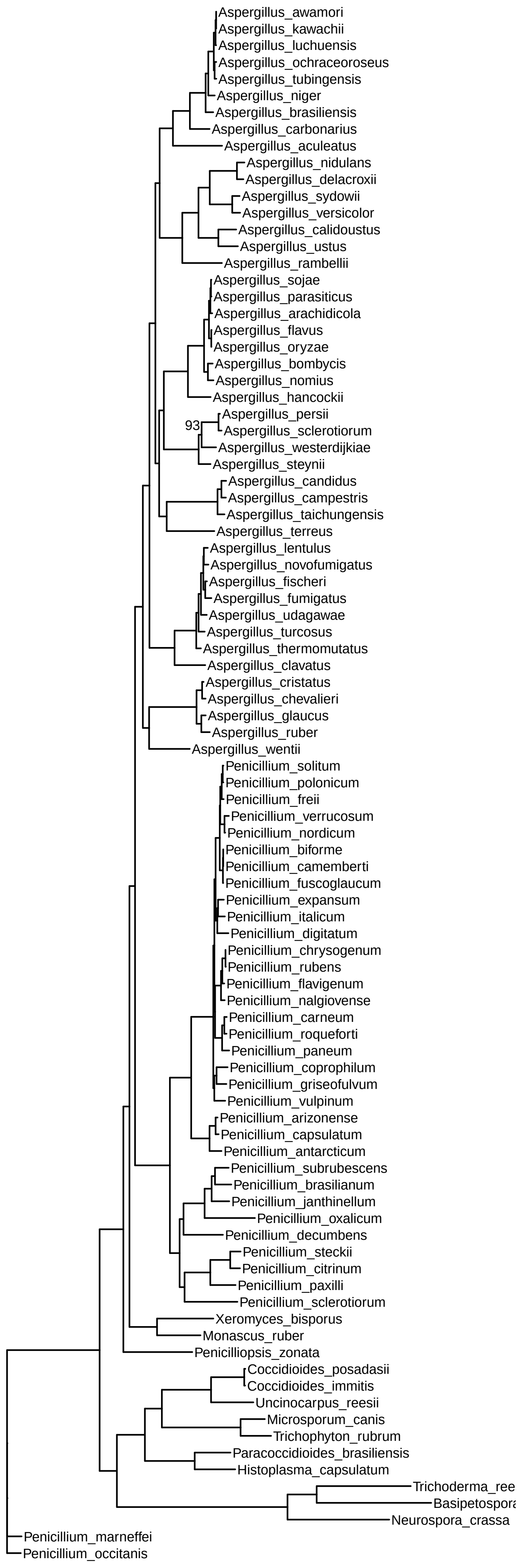

Aspergillaceae_raxml_ng_run1_Concatenation_I

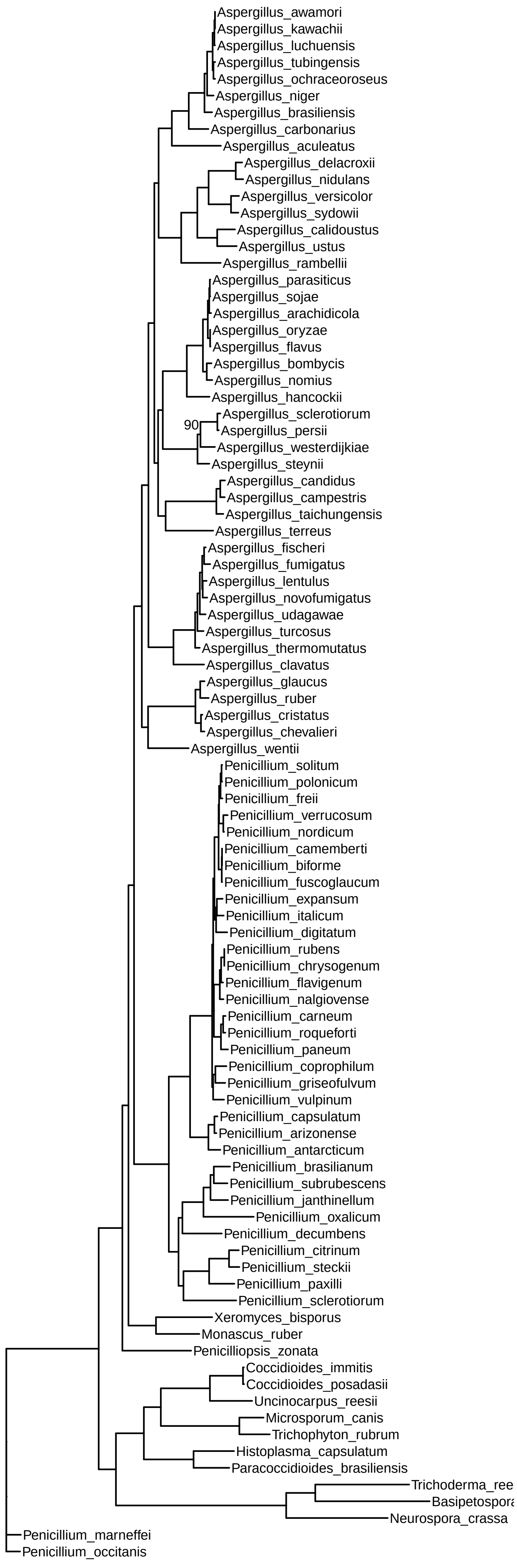

Aspergillaceae_raxml_ng_run2_Concatenation_I

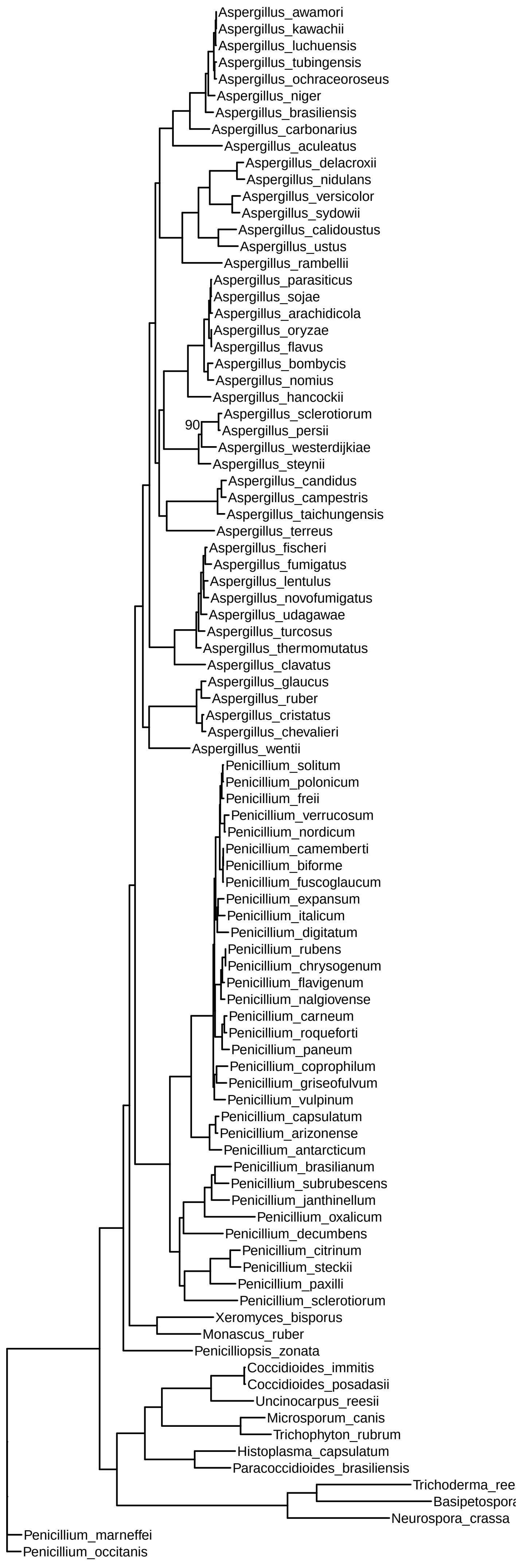

Saccharomycotina_Cell_iqtree_run1_Concatena

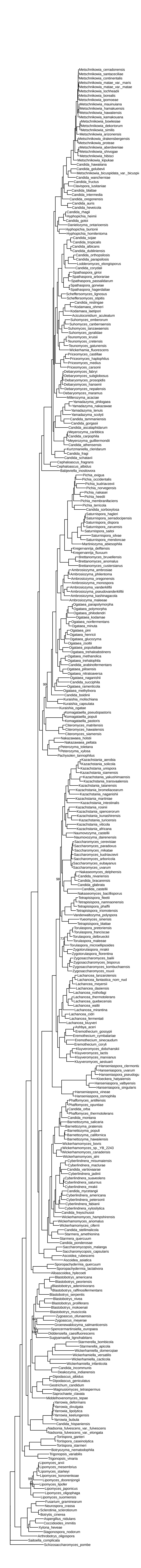

Saccharomycotina_Cell_iqtree_run2_Concatena

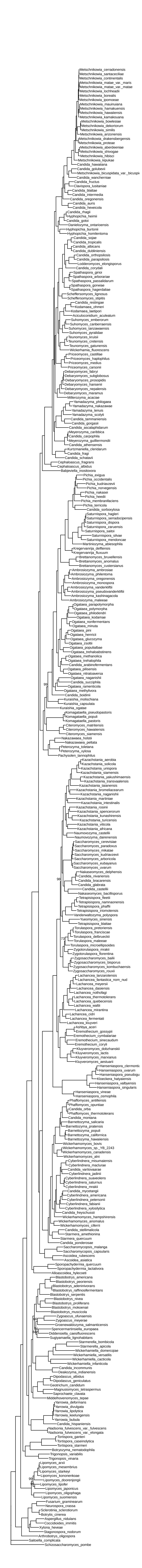

Saccharomycotina_Cell_raxml_ng_run1_Concat

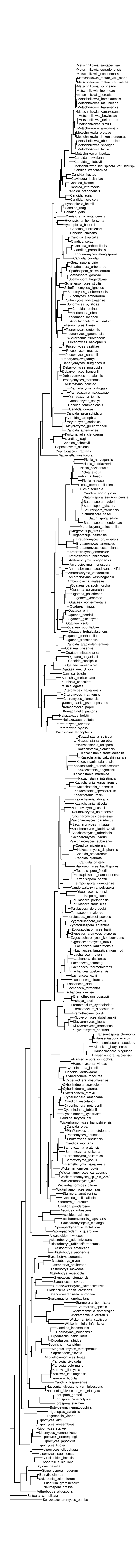

Saccharomycotina_Cell_raxml_ng_run2_Concat

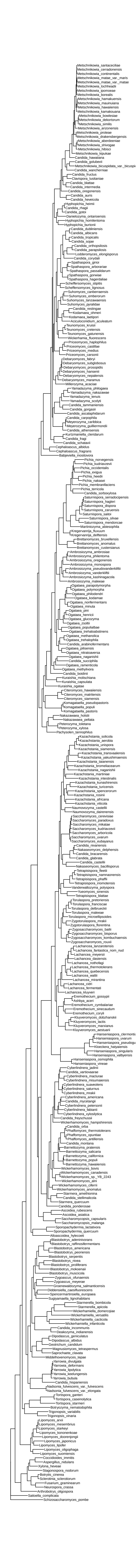

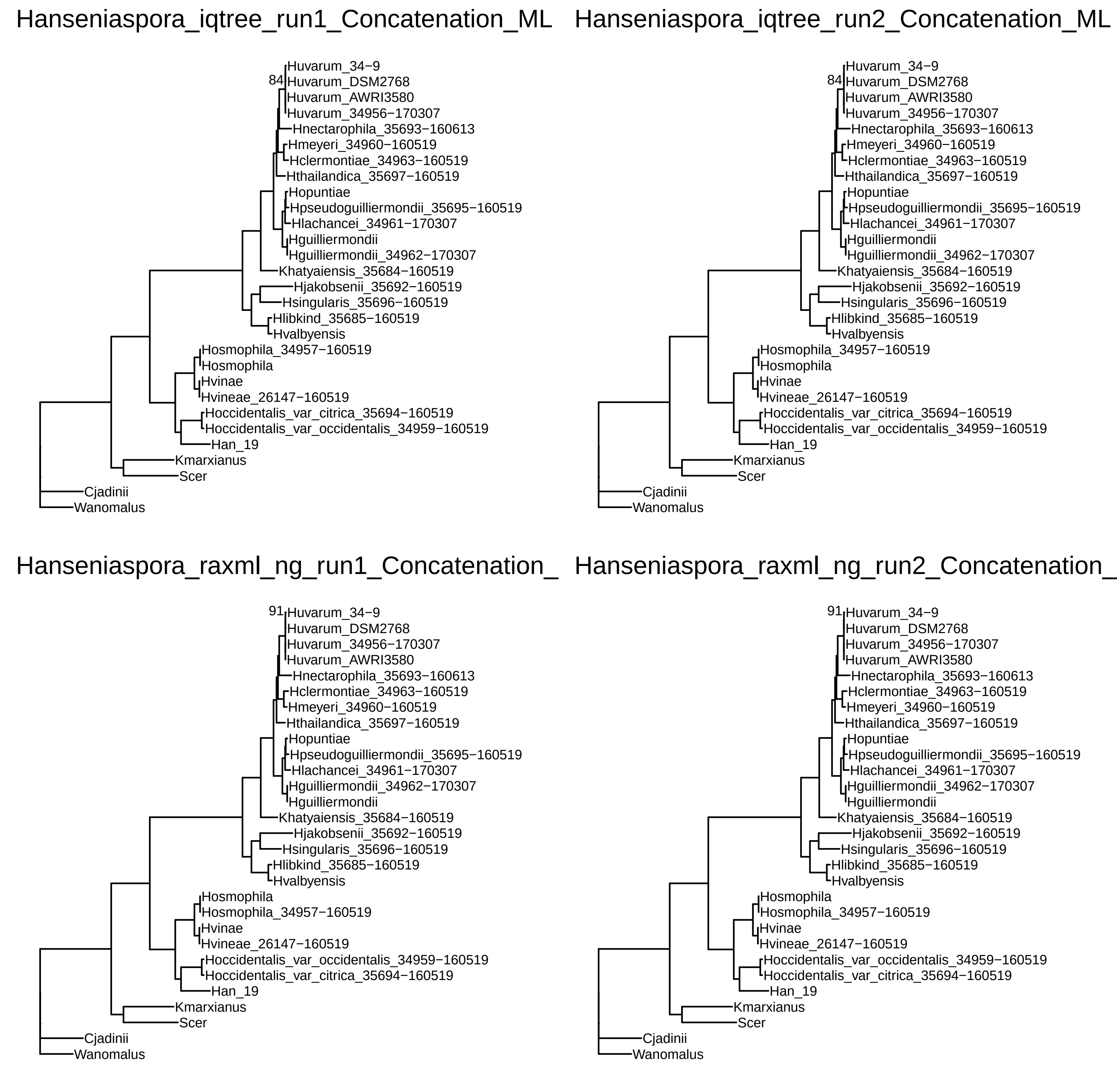
Hanseniaspora_iqtree_run1_Concatenation_ML
84
Huvarum_34−9
Huvarum_DSM2768
Huvarum_AWRI3580
Huvarum_34956−170307
Hnectarophila_35693−160613
Hmeyeri_34960−160519
Hclermontiae_34963−160519
Hthailandica_35697−160519
Hopuntiae
Hpseudoguilliermondii_35695−160519
Hlachancei_34961−170307
Hguilliermondii
Hguilliermondii_34962−170307
Khatyaiensis_35684−160519
Hjakobsenii_35692−160519
Hsingularis_35696−160519
Hlibkind_35685−160519
Hvalbyensis
Hosmophila_34957−160519
Hosmophila
Hvinae
Hvineae_26147−160519
Hoccidentalis_var_citrica_35694−160519
Hoccidentalis_var_occidentalis_34959−160519
Han_19
Kmarxianus
Scer
Cjadinii
Wanomalus
Hanseniaspora_iqtree_run2_Concatenation_ML
84
Huvarum_34−9
Huvarum_DSM2768
Huvarum_AWRI3580
Huvarum_34956−170307
Hnectarophila_35693−160613
Hmeyeri_34960−160519
Hclermontiae_34963−160519
Hthailandica_35697−160519
Hopuntiae
Hpseudoguilliermondii_35695−160519
Hlachancei_34961−170307
Hguilliermondii
Hguilliermondii_34962−170307
Khatyaiensis_35684−160519
Hjakobsenii_35692−160519
Hsingularis_35696−160519
Hlibkind_35685−160519
Hvalbyensis
Hosmophila_34957−160519
Hosmophila
Hvinae
Hvineae_26147−160519
Hoccidentalis_var_citrica_35694−160519
Hoccidentalis_var_occidentalis_34959−160519
Han_19
Kmarxianus
Scer
Cjadinii
Wanomalus
Hanseniaspora_raxml_ng_run1_Concatenation_
91
Huvarum_34−9
Huvarum_DSM2768
Huvarum_34956−170307
Huvarum_AWRI3580
Hnectarophila_35693−160613
Hclermontiae_34963−160519
Hmeyeri_34960−160519
Hthailandica_35697−160519
Hopuntiae
Hpseudoguilliermondii_35695−160519
Hlachancei_34961−170307
Hguilliermondii_34962−170307
Hguilliermondii
Khatyaiensis_35684−160519
Hjakobsenii_35692−160519
Hsingularis_35696−160519
Hlibkind_35685−160519
Hvalbyensis
Hosmophila
Hosmophila_34957−160519
Hvinae
Hvineae_26147−160519
Hoccidentalis_var_occidentalis_34959−160519
Hoccidentalis_var_citrica_35694−160519
Han_19
Kmarxianus
Scer
Cjadinii
Wanomalus
Hanseniaspora_raxml_ng_run2_Concatenation_
91
Huvarum_34−9
Huvarum_DSM2768
Huvarum_34956−170307
Huvarum_AWRI3580
Hnectarophila_35693−160613
Hclermontiae_34963−160519
Hmeyeri_34960−160519
Hthailandica_35697−160519
Hopuntiae
Hpseudoguilliermondii_35695−160519
Hlachancei_34961−170307
Hguilliermondii_34962−170307
Hguilliermondii
Khatyaiensis_35684−160519
Hjakobsenii_35692−160519
Hsingularis_35696−160519
Hlibkind_35685−160519
Hvalbyensis
Hosmophila
Hosmophila_34957−160519
Hvinae
Hvineae_26147−160519
Hoccidentalis_var_occidentalis_34959−160519
Hoccidentalis_var_citrica_35694−160519
Han_19
Kmarxianus
Scer
Cjadinii
Wanomalus

Rhizoplaca_iqtree_run1_Concatenation_ML

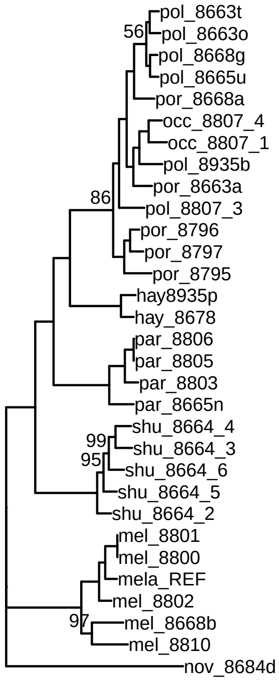

Rhizoplaca_iqtree_run2_Concatenation_ML

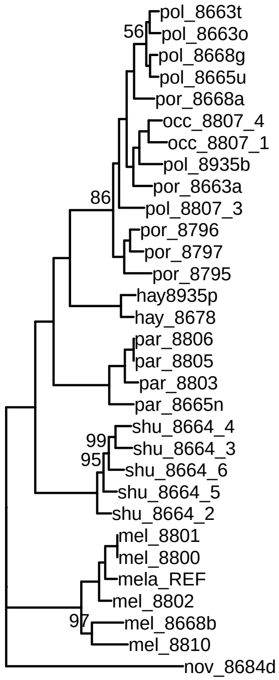

Rhizoplaca_raxml_ng_run1_Concatenation_ML

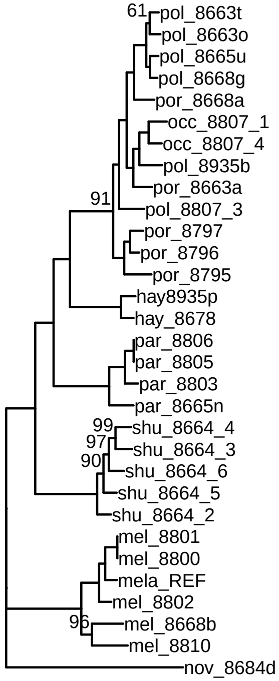

Rhizoplaca_raxml_ng_run2_Concatenation_ML

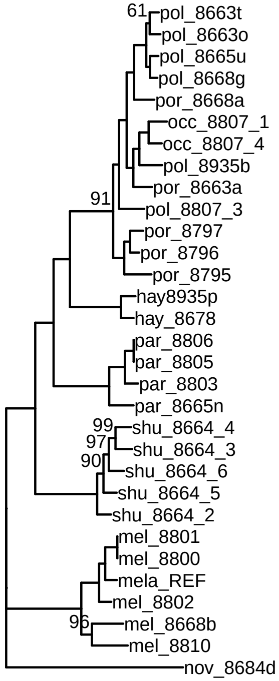

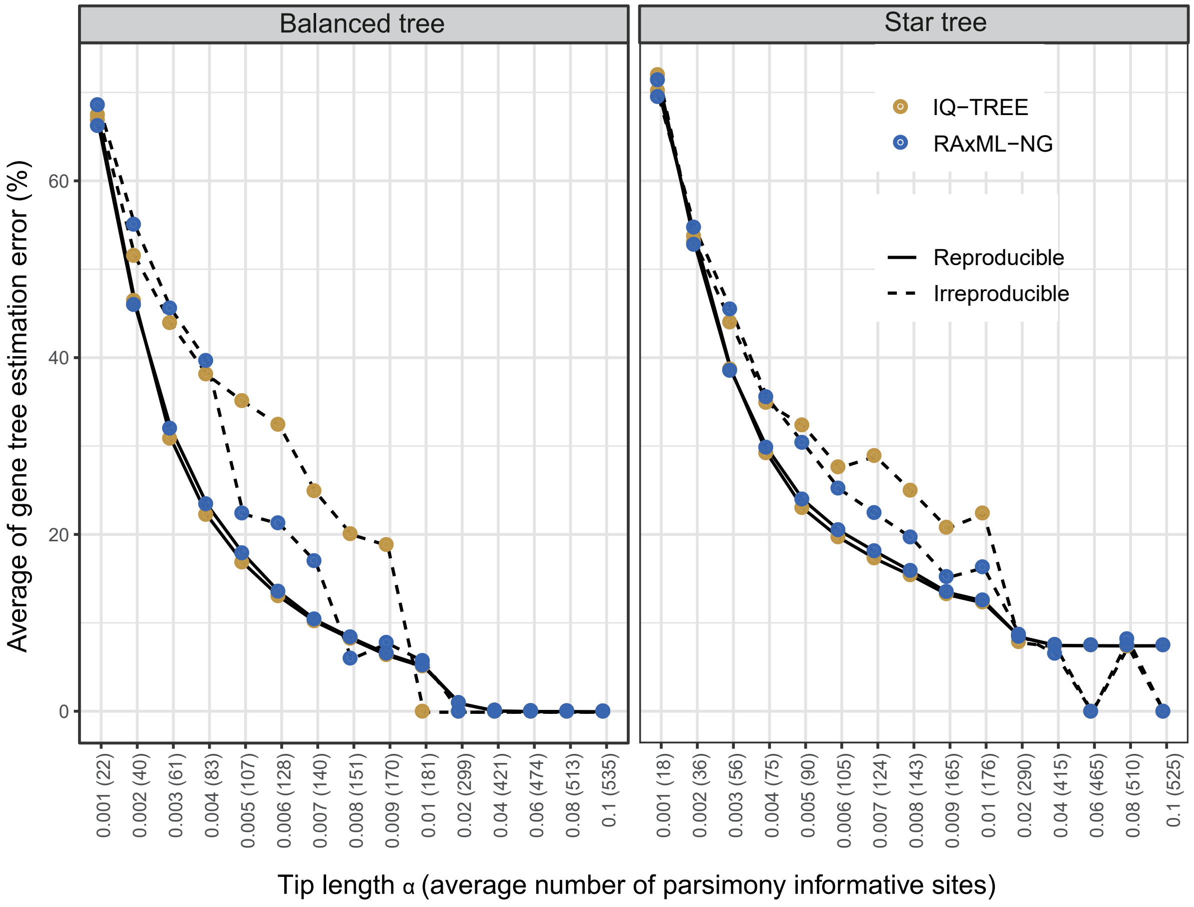

Supplementary Figure 14. Effect of parsimony-informative sites on gene tree estimation error. For each of 15 data points (α = 0.001, 0.002, 0.003, 0.004, 0.005, 0.006, 0.007, 0.008, 0.009, 0.01, 0.02, 0.04, 0.06, 0.08, or 0.1), we simulated 500 nucleotide gene alignments, varying randomly in length from 300 to 1,000 base pairs with balanced tree or star tree (see Fig. 5a and 5b). For each gene alignment, its reproducibility was assessed between two replicates (Run1 and Run2) (i.e., each analysis was run on a single node, but Run1 was executed on a different node than Run2) using 2 threads on the CHTC cluster when using IQ-TREE (in yellow) and RAxML-NG (in blue), respectively. Gene tree estimation error is a normalized Robinson–Foulds tree distance between inferred gene tree and the reference species tree used in simulation. Overall, the average of gene tree estimation errors decreased with increasing number of parsimony-informative sites, but irreproducible genes exhibited higher levels of gene tree estimation error than reproducible genes.

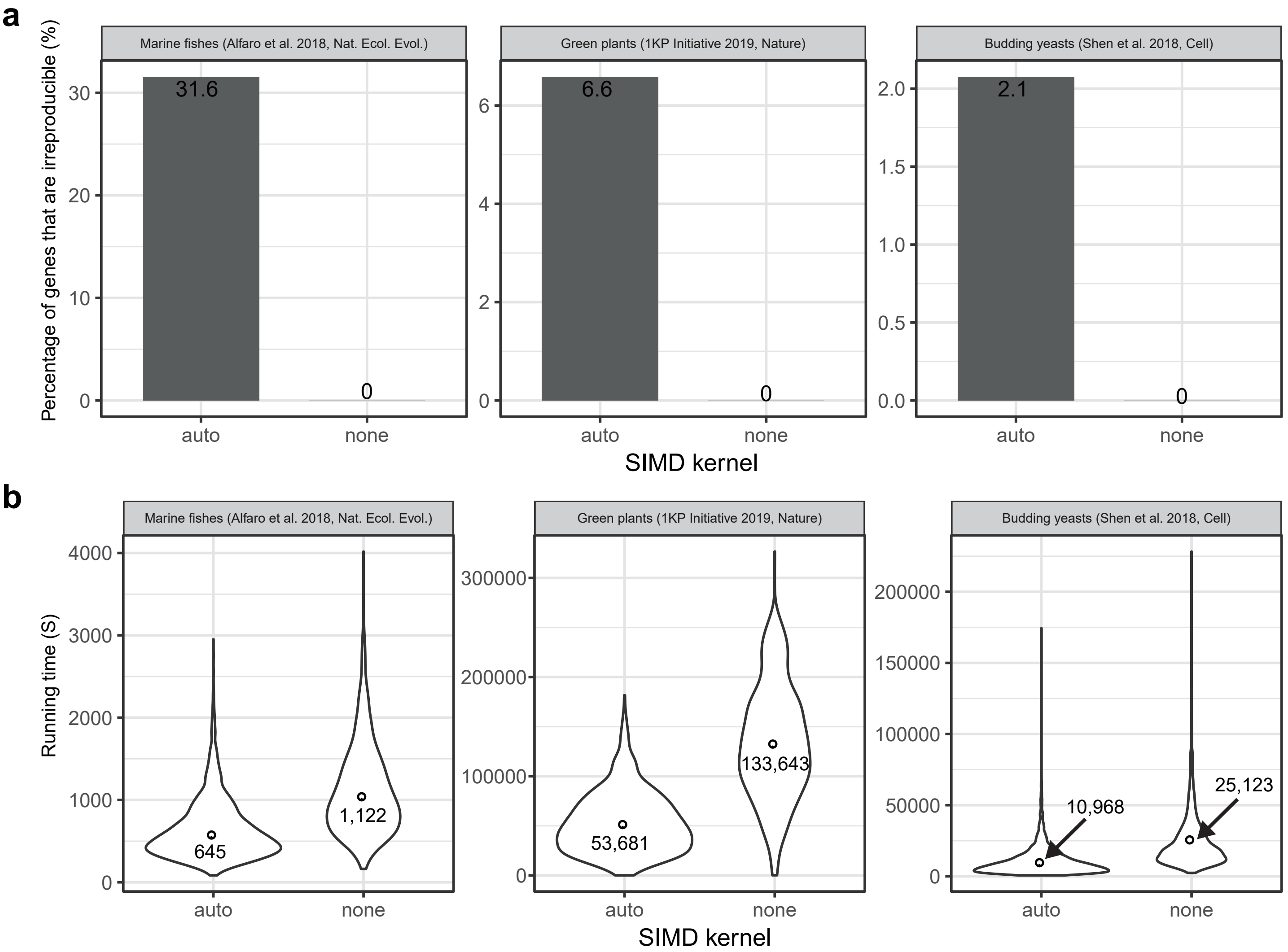

Supplementary Figure 15. Comparison of runtime performance of optimal vector kernel (auto) and non-vectorized kernel (none) using RAxML-NG. RAxML-NG can automatically detect the best set of vector instructions available on the processor architecture, and use the respective computational kernels to achieve optimal performance. For each of 3,819 genes from three representative studies (1,001 genes in the Marine fishes study, 410 genes in the Green plants study, 2408 genes in the Budding yeasts study), we ran two replicates (Run1 and Run2) jobs on two separate nodes (i.e., each analysis was run on a single node, but Run1 was executed on a different node than Run2) using RAxML-NG with the additional "—simd" option either set to "—simd auto" or to "—simd none". (a) Percentage of genes that yielded irreproducible phylogenies under the "—simd auto" and "—simd none" options, respectively. (b) Distribution of running time (seconds) under the two options. Black dots in violin plots denote mean values. All 15,276 jobs (3,819 genes * 2 replicates * 1 program * 2 experiments) were running using 2 threads per node on the Center for High Throughput Computing (CHTC) at the University of Wisconsin-Madison. Our results show all genes were reproducible under the "—simd none" option, but they required much longer runtimes.

Supplementary References

1. Blaimer, B. B., Mawdsley, J. R. & Brady, S. G. Multiple origins of sexual dichromatism and aposematism within large carpenter bees. *Evolution (N. Y).* **72**, 1874–1889 (2018).
2. Prum, R. O. *et al.* A comprehensive phylogeny of birds (Aves) using targeted next-generation DNA sequencing. *Nature* **526**, 569–573 (2015).
3. Espeland, M. *et al.* A Comprehensive and Dated Phylogenomic Analysis of Butterflies. *Curr. Biol.* **28**, 770-778.e5 (2018).
4. Blom, M. P. K., Bragg, J. G., Potter, S. & Moritz, C. Accounting for uncertainty in gene tree estimation: Summary-coalescent species tree inference in a challenging radiation of Australian lizards. *Syst. Biol.* **66**, 352–366 (2017).
5. Alfaro, M. E. *et al.* Explosive diversification of marine fishes at the Cretaceous–Palaeogene boundary. *Nat. Ecol. Evol.* **2**, 688–696 (2018).
6. Roycroft, E. J., Moussalli, A. & Rowe, K. C. Phylogenomics Uncovers Confidence and Conflict in the Rapid Radiation of Australo-Papuan Rodents. *Syst. Biol.* **0**, 1–14 (2019).
7. Herrando-Moraira, S. *et al.* Exploring data processing strategies in NGS target enrichment to disentangle radiations in the tribe Cardueae (Compositae). *Mol. Phylogenet. Evol.* **128**, 69–87 (2018).
8. Yang, Y. *et al.* Dissecting molecular evolution in the highly diverse plant clade caryophyllales using transcriptome sequencing. *Mol. Biol. Evol.* **32**, 2001–2014 (2015).
9. One Thousand Plant Transcriptomes Initiative. One thousand plant transcriptomes and the phylogenomics of green plants. *Nature* **574**, 679–685 (2019).
10. Wu, M., Kostyun, J. L., Hahn, M. W. & Moyle, L. C. Dissecting the basis of novel trait evolution in a radiation with widespread phylogenetic discordance. *Mol. Ecol.* **27**, 3301–3316 (2018).
11. Mitchell, N., Lewis, P. O., Lemmon, E. M., Lemmon, A. R. & Holsinger, K. E. Anchored phylogenomics improves the resolution of evolutionary relationships in the rapid radiation of *protea* L. *Am. J. Bot.* **104**, 102–115 (2017).
12. Steenwyk, J. L., Shen, X.-X., Lind, A. L., Goldman, G. H. & Rokas, A. A Robust Phylogenomic Time Tree for Biotechnologically and Medically Important Fungi in the Genera *Aspergillus* and *Penicillium*. *MBio* **10**, 1–25 (2019).
13. Shen, X.-X. *et al.* Tempo and Mode of Genome Evolution in the Budding Yeast Subphylum. *Cell* **175**, 1533–1545 (2018).
14. Steenwyk, J. L. *et al.* Extensive loss of cell-cycle and DNA repair genes in an ancient lineage of bipolar budding yeasts. *PLOS Biol.* **17**, e3000255 (2019).
15. Leavitt, S. D. *et al.* Resolving evolutionary relationships in lichen-forming fungi using diverse phylogenomic datasets and analytical approaches. *Sci. Rep.* **6**, 22262 (2016).
16. Stamatakis, A. RAxML version 8: A tool for phylogenetic analysis and post-analysis of large phylogenies. *Bioinformatics* **30**, 1312–1313 (2014).
17. Shimodaira, H. An approximately unbiased test of phylogenetic tree selection. *Syst. Biol.* **51**, 492–508 (2002).

18. Rambaut, A. & Grass, N. C. Seq-Gen: an application for the Monte Carlo simulation of DNA sequence evolution along phylogenetic trees. *Bioinformatics* **13**, 235–238 (1997).
19. Nguyen, L.-T., Schmidt, H. A., von Haeseler, A. & Minh, B. Q. IQ-TREE: A Fast and Effective Stochastic Algorithm for Estimating Maximum-Likelihood Phylogenies. *Mol. Biol. Evol.* **32**, 268–274 (2015).
20. Kozlov, A. M., Darriba, D., Flouri, T., Morel, B. & Stamatakis, A. RAxML-NG: a fast, scalable and user-friendly tool for maximum likelihood phylogenetic inference. *Bioinformatics* **35**, 4453–4455 (2019).
21. Biczok, R. *et al.* Two C++ libraries for counting trees on a phylogenetic terrace. *Bioinformatics* **34**, 3399–3401 (2018).
22. Sanderson, M. J., McMahon, M. M., Stamatakis, A., Zwickl, D. J. & Steel, M. Impacts of Terraces on Phylogenetic Inference. *Syst. Biol.* **64**, 709–726 (2015).
