# Supplementary material for: An investigation of irreproducibility in maximum likelihood phylogenetic inference
Source: Nat Commun. 2020 Nov 30;11:6096. doi: 10.1038/s41467-020-20005-6 (PMC7705714; doi:10.1038/s41467-020-20005-6)
Supplement: Supplementary file 3 — Description of Additional Supplementary Files [file 41467_2020_20005_MOESM3_ESM.pdf]

Description of Additional Supplementary Files

File name: Supplementary Data 1
Description: The list of 15 phylogenomic datasets examined in this study.

File name: Supplementary Data 2
Description: Summary of irreproducibility among 15 studies using 2 threads per node on CHTC cluster at the University of Wisconsin-Madison.

File name: Supplementary Data 3
Description: Summary of irreproducibility among 15 studies using 2 threads per node on ACCRE cluster at Vanderbilt University.

File name: Supplementary Data 4
Description: Summary of effects of computing resources on irreproducibility of phylogenies among three representative studies.

File name: Supplementary Data 5
Description: Summary of gene tree estimation errors between irreproducible phylogenies and reproducible phylogenies using simulated data.
